# Protein Kinase A Activation Promotes Cancer Cell Resistance to Glucose Starvation and *Anoikis*

**DOI:** 10.1371/journal.pgen.1005931

**Published:** 2016-03-15

**Authors:** Roberta Palorini, Giuseppina Votta, Yuri Pirola, Humberto De Vitto, Sara De Palma, Cristina Airoldi, Michele Vasso, Francesca Ricciardiello, Pietro Paolo Lombardi, Claudia Cirulli, Raffaella Rizzi, Francesco Nicotra, Karsten Hiller, Cecilia Gelfi, Lilia Alberghina, Ferdinando Chiaradonna

**Affiliations:** 1 Department of Biotechnology and Biosciences, University of Milano-Bicocca, Milan, Italy; 2 SYSBIO Center for Systems Biology, Department of Biotechnology and Biosciences, University of Milano-Bicocca, Milan, Italy; 3 Luxembourg Centre for Systems Biomedicine, Esch-sur-Alzette, Luxembourg; 4 Department of Informatics Systems and Communication, University of Milano-Bicocca, Milan, Italy; 5 Institute of Molecular Bioimaging and Physiology (IBFM), CNR, Segrate-Cefalù, Italy; 6 Department of Biomedical Sciences for Health, University of Milan, Segrate, Milan, Italy; University of Notre Dame, UNITED STATES

## Abstract

Cancer cells often rely on glycolysis to obtain energy and support anabolic growth. Several studies showed that glycolytic cells are susceptible to cell death when subjected to low glucose availability or to lack of glucose. However, some cancer cells, including glycolytic ones, can efficiently acquire higher tolerance to glucose depletion, leading to their survival and aggressiveness. Although increased resistance to glucose starvation has been shown to be a consequence of signaling pathways and compensatory metabolic routes activation, the full repertoire of the underlying molecular alterations remain elusive. Using omics and computational analyses, we found that cyclic adenosine monophosphate-Protein Kinase A (cAMP-PKA) axis activation is fundamental for cancer cell resistance to glucose starvation and *anoikis*. Notably, here we show that such a PKA-dependent survival is mediated by parallel activation of autophagy and glutamine utilization that in concert concur to attenuate the endoplasmic reticulum (ER) stress and to sustain cell anabolism. Indeed, the inhibition of PKA-mediated autophagy or glutamine metabolism increased the level of cell death, suggesting that the induction of autophagy and metabolic rewiring by PKA is important for cancer cellular survival under glucose starvation. Importantly, both processes actively participate to cancer cell survival mediated by suspension-activated PKA as well. In addition we identify also a PKA/Src mechanism capable to protect cancer cells from *anoikis*. Our results reveal for the first time the role of the versatile PKA in cancer cells survival under chronic glucose starvation and *anoikis* and may be a novel potential target for cancer treatment.

## Author Summary

Tumor heterogeneity exists in many human cancers, and it has been shown that it can play a role in tumor progression. Indeed, cell diversity may be critically important when tumors experience selective pressures, like nutrient deprivation, hypoxia, chemotherapy. PKA, through incompletely understood mechanisms, controls several cellular processes like cell growth, cell differentiation, cell metabolism, cell migration and, as more recently observed, also cancer progression. In this work, we show that activation of PKA induces the ability of a cancer cell sub-population to survive under strong stress conditions namely nutrient deprivation and cell detachment. Indeed, PKA activation in these cells results in autophagy induction, and at the same time, in activation of glutamine metabolism and Src kinase. Importantly, blocking directly the PKA pathway, as well as the autophagy, the glutamine metabolism or the Src pathway by inhibitory drugs, almost completely prevents cell growth of this sub-population of resistant cancer cells. These results suggest that drugs, targeting especially PKA pathway as well as downstream processes like autophagy, glutamine metabolism and Src signaling, may specifically inhibit cancer cells ability to survive under selective pressure favoring cancer resistance.

## Introduction

Transformed cells are often characterized by an enhanced use of glucose to support anabolic growth [1,2]. In this regard, different studies have shown that several cancer cells, grown either in low glucose availability or in free glucose, are strongly susceptible to cell death when compared to normal counterparts [3,4,5]. The molecular mechanisms that underlie this response are complex, cell-type specific and not yet fully clarified. Cell death has been associated with metabolic deficiencies, due likely to reduced ability to uptake glucose or to mitochondrial dysfunctions [6], with inactivation of controlling mechanisms, such as the one activated by AMP-activated protein kinase (AMPK) through p53 and hyperactivation of pro-survival mechanisms like mammalian target of rapamycin (mTOR) pathway [7,8,9,10], as well as with the induction of Endoplasmic Reticulum (ER) stress and cell detachment [11,12,13]. In particular, these latter processes, especially if triggered for a prolonged time, may lead either to cell death or to the selection of resistant tumor cells, sometimes characterized by distinct metabolic features and catabolic activities [14]. Accordingly, other works have shown that cancer cells, on acquiring higher tolerance to glucose depletion, activate compensatory signaling pathways and metabolic routes, for instance fatty acid oxidation [15,16,17]. Importantly, such resistant cancer cells, which are often more aggressive, may be selected after therapies exploiting synergism between chemotherapeutic treatments and anti-metabolic drugs [18] or after genetic and pharmacological ablation of oncogenic pathways which may lead to poor patient survival [19]. Altogether these findings suggest the ability of cancer cells to survive in glucose starvation by induction of adaptive processes. Exploitation of these processes as putative therapeutic targets may represent an important goal in cancer therapy.

The ubiquitous second messenger cyclic adenosine monophosphate (cAMP) is a key regulator of metabolic activity, survival, proliferation and differentiation in a wide variety of cell types. Accumulated evidence has indicated that cAMP controls all these complex cellular processes via changes in target gene transcription primarily through the activation of one downstream effector, the cAMP-dependent protein kinase or Protein Kinase A (PKA). Upon binding of cAMP to the regulatory subunits, the catalytic subunits of PKA phosphorylate and modulate the activity of a variety of cytosolic and nuclear substrates, including the transcription factor cAMP response element-binding protein (CREB) [20].

Our previous work showed that the exogenous activation of PKA pathway promotes cancer cell survival under glucose starvation, especially by modulating mitochondrial function. In fact we showed that PKA activation induces mitochondrial Complex I activity, mitochondrial fusion and decreases intracellular reactive oxygen species (ROS) levels [21]. Furthermore, we also showed that positive modulation of Hexosamine Biosynthetic Pathway (HBP), through its effects on ER homeostasis, is also an important road to control cancer cell survival in glucose starvation [12]. Prompted by this evidence, in this report we have explored the extent to which PKA pathway and HBP/ER axis are linked in inducing cancer cell survival in glucose starvation.

Using mouse and human cancer cells sensitive to glucose limitation (glucose concentrations are decreased to less than 1mM as within solid tumor masses [22]) and a combination of omics systems biology-oriented technologies, such as microarray expression profiles, 2-D difference gel electrophoresis (2D-DIGE) proteomic analysis, Nuclear Magnetic Resonance (NMR)-based metabolomics followed by analysis through different computational methods such as STRING 9.1 [23], PIANO [24] and MetaboAnalyst 3.0 [25], we identified novel mechanisms for the PKA signaling in the adaptive response to glucose deprivation and the resulting cell detachment. In particular we show that activation of PKA in cancer cells, upon glucose starvation, leads to the enhancement of autophagy, glutamine metabolism and Src activation that together concur to ER stress attenuation and *anoikis*-resistance. Conversely, inhibition of these pathways leads to a robust suppression of cancer cell survival, especially of detached *anoikis*-resistant cells, indicating that PKA is a potential chemotherapeutic target to prevent cancer cell metastasis.

## Results

### Protein processing in ER and catabolism are highly enriched pathways in Transformed cells upon PKA activation

**Fig 1:**
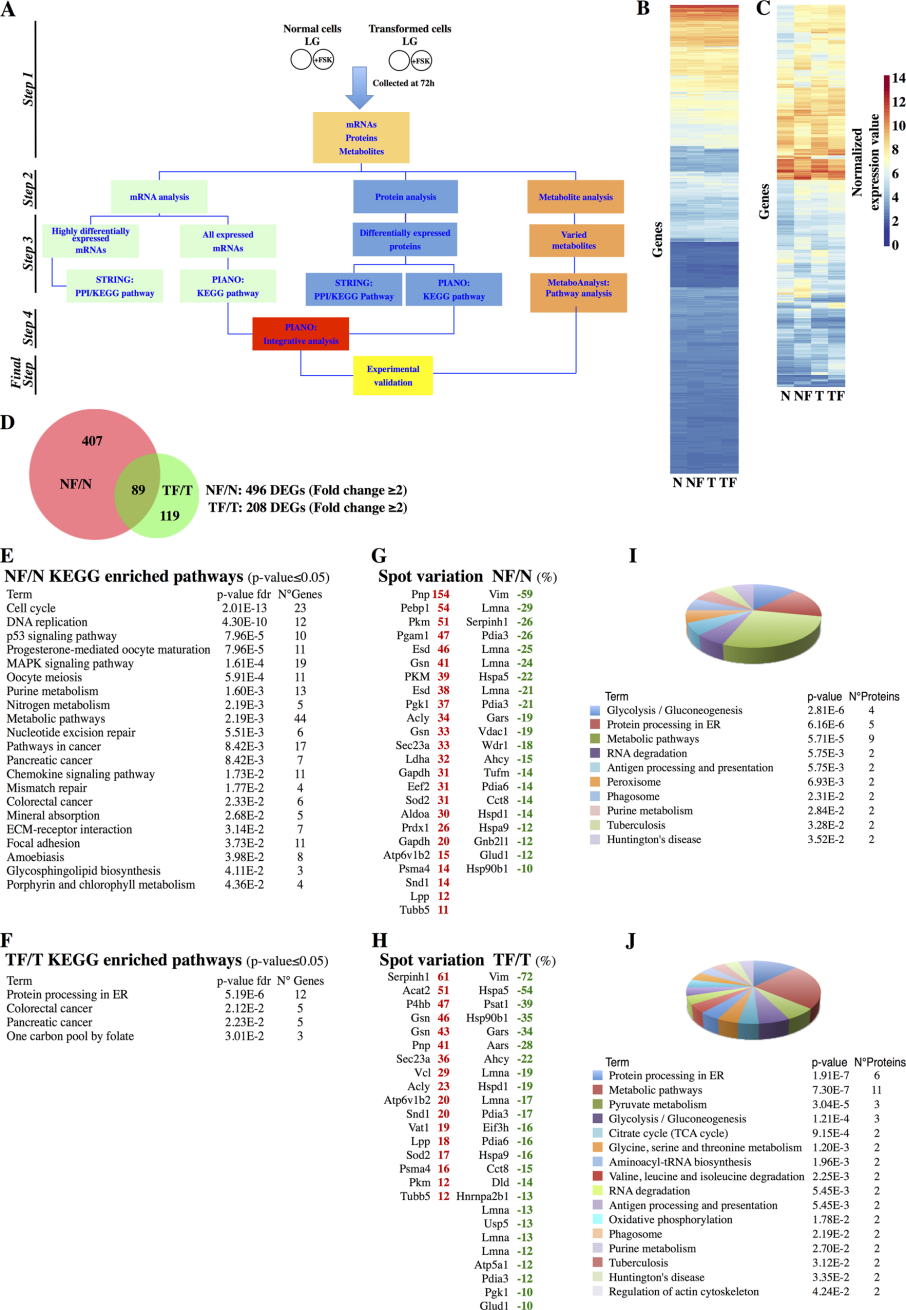
Transcriptomic and proteomic analysis of Normal and Transformed cells treated with FSK. (**A)** Workflow followed to analyze the transcriptomic, proteomic and metabolomic data. (**B-C)** Hierarchical clustering of 17669 differentially expressed genes of Normal cells and Transformed cells -/+ FSK (B) and 615 genes having fold change ≥2 in the two comparisons NF/N and TF/T (C). (**D)** Venn diagram representing the distribution of the 615 genes in the two comparisons. (**E-F)** Enriched KEGG pathways obtained by STRING analysis of the 496 and 208 DEGs in the comparisons NF/N (E) and TF/T (F) (fold change ≥2). (**G-H)** In red the up-regulated proteins and in green the down-regulated ones identified in proteomic analysis in the comparison NF/N (G) and TF/T (H). (**I-J)** Enriched KEGG pathways obtained by STRING analysis of the differentially expressed proteins in the two comparisons NF/N (I) and TF/T (J). The dimension of the pie slice is referred to the number of proteins associated to each pathway. The bottom list represents the identified pathways, depicted by using a color code, the number of proteins belonging to the pathway and the p-value (p-value <0.05).

As depicted in Fig 1A, to analyze the difference between normal and cancer cells under low glucose availability and upon activation of PKA pathway, we used NIH3T3 cells (Normal), a genetically well-characterized immortalized cell line that has long been established as a model of “normal” cells to study cell transformation, and NIH3T3-Kras cells (Transformed), an isogenic cell line expressing an oncogenic form of K-Ras able to induce cell transformation and tumor in immunocompromised mice [26]. Both cell lines were cultured for 72h in 1mM glucose (Low Glucose, LG) and upon daily treatment with 10μM Forskolin (FSK), a strong activator of PKA pathway, as shown in several reports [27]. In line with our previous studies, this time point was chosen because at 72h both cell lines showed a FSK-independent complete depletion of glucose from the culture medium [21]. The experimental scheme for the treatments, the specific effect of FSK on PKA activity, as measured by Western blot of phosphorylated substrates of PKA (p-PKAs) and of phospho-CREB Serine133 (pCREB S133) and by a PKA ELISA assay, and on cell survival are shown in panels A, B and C in S1 Fig. To gain insight into the global cellular processes altered by glucose depletion and by the PKA activation, we measured gene expression, protein and metabolite changes in both cell lines by using dedicated tools. Highly differentially expressed mRNAs (fold change ≥2 in the comparison of treated *vs*. untreated, DEGs) (Fig 1A, green boxes, and 1C) were analyzed by using STRING database to retrieve their protein-protein interaction information and to evaluate the enrichment in KEGG pathways [23]. Conversely, the complete list of regulated genes (Fig 1A, green boxes, and 1B) was analyzed by using PIANO program to perform Gene Set Enrichment Analyses (GSEA) [24]. Both approaches were used also for protein analysis (Fig 1A, blue boxes). Metabolites (Fig 1A, orange boxes) were analyzed by MetaboAnalyst program to identify the association between identified metabolites and specific metabolic pathways [25].

To determine the effect of PKA activation on gene expression of both cell lines, we performed genome-wide expression profiling using Affymetrix arrays. FSK-treated cells (from now indicated as NF and TF, denoting, respectively Normal and Transformed treated cells) displayed clearly different gene expression pattern compared to untreated ones (from now indicated as N and T, denoting, respectively Normal and Transformed cells) (Fig 1B), and such a difference was more evident by analysis of the DEGs in which strong differences were observed also between the NF/N and TF/T comparisons (Fig 1C and 1D). DEGs were also used for protein-protein interaction identification and functional annotation. NF/N DEGs identified a large network formed by 3569 protein-protein interactions, suggesting a high degree of connections among the nodes in the network (S2 Fig). Their functional analysis, by using KEGG pathways, revealed an enrichment of repressed genes involved in the regulation of cell cycle as well as of DNA-related pathways (Fig 1E). These findings were in line with our expectations regarding Normal cell responses to FSK, which involve cell cycle inhibition and proliferation arrest [21]. However for TF/T DEGs, as shown in S3 Fig, the network included a much smaller number of protein-protein interactions, 141, suggesting a low degree of connections and indicating that their functions were unrelated or unknown. In fact, only four pathways were specifically enriched, among which only two were associated with a clear recognizable cellular process namely *Protein processing in ER* and *One carbon pool by folate* (Fig 1F). To assess proteomic changes, 2D-DIGE analyses of the protein extracts from the two above described comparisons were performed. Overall, 120 spots were differentially expressed in the two comparisons (S2 Table). In Fig 1G and 1H the up-regulated (red color) and down-regulated (green color) proteins for each comparison have been indicated. Functional analysis revealed cellular metabolism and *Protein processing in ER* amongst the most significant pathways enriched in both comparisons (Fig 1I and 1J). However several metabolic enzymes, for instance the ones related to *Glycolysis/Gluconeogenesis* (*PGAM1*, *PGK1*, *GAPDH*, *ALDOA*, *PKM* and *LDHA*), were up-regulated in NF/N only, pointing the opposite regulation of the metabolic pathways in the two comparisons. Conversely, upon FSK stimulation both cell types showed a decreased expression of proteins identifying *Protein processing in ER* (Fig 1I and 1J). Protein-protein interaction identification and functional annotation by using STRING, almost confirmed the above results, since in the NF/N and TF/T comparisons a major modulation of metabolic processes and the *Protein processing in ER* were respectively identified (Panels A and B in S4 Fig).

To further identify differences between the two cell lines upon FSK treatment, we performed a GSEA by using all expressed mRNAs, comprising a set of 17669 transcripts (S3 Table), the proteomic data (S2 Table), alone and in combination, and by applying PIANO method [24]. This tool provides not only GSEA but also additional information about directionality of the enrichment (namely up-regulated, down-regulated or mixed direction of genes or proteins identifying the pathway).

**Fig 2:**
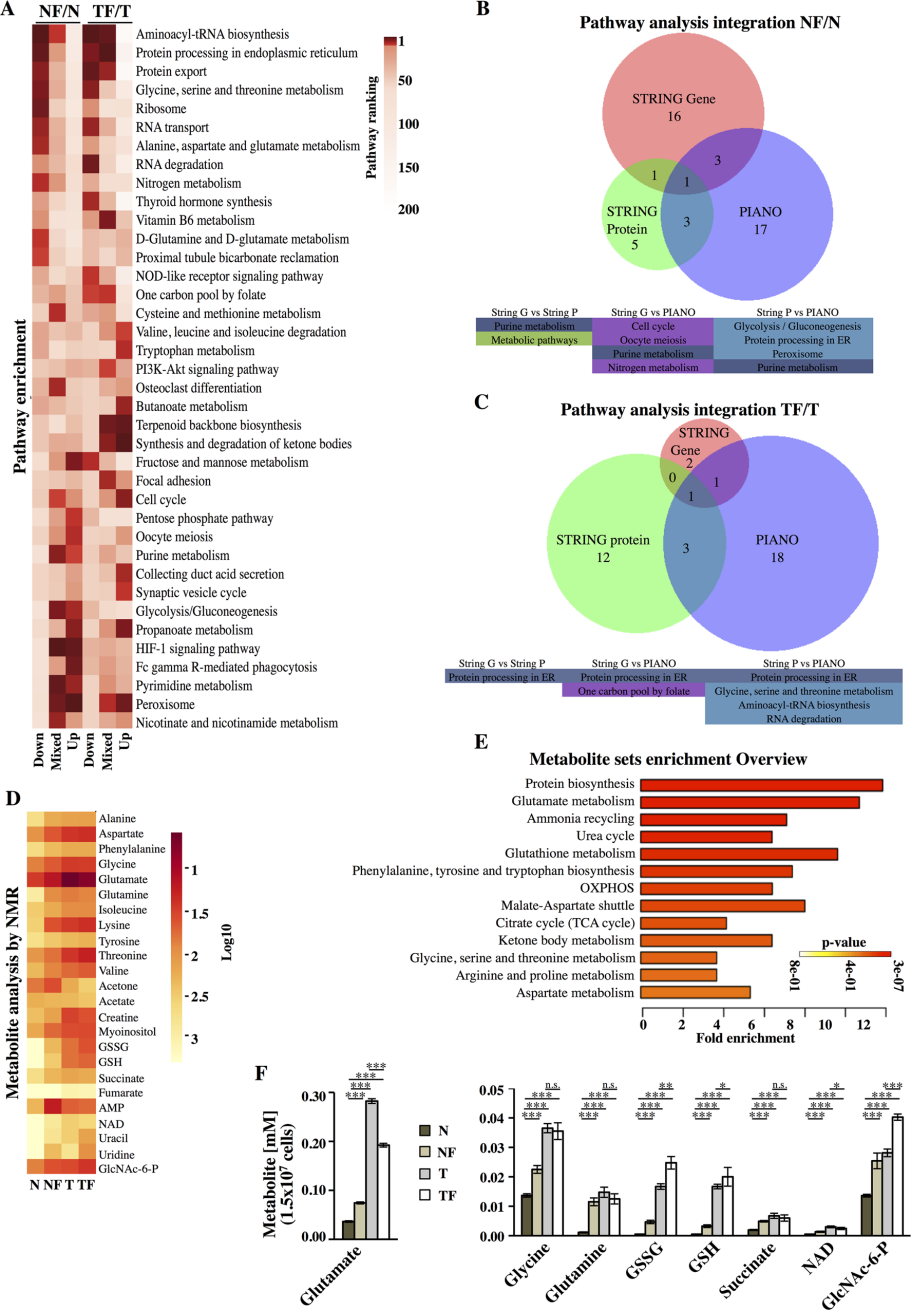
Transcriptomic/proteomic integrative analysis and NMR-based metabolomics of Normal and Transformed cells treated with FSK. (**A)** Heatmap of the top 10-ranked pathways obtained by PIANO tool applied to gene and protein datasets. (**B-C)** Venn diagrams indicate the number of shared pathways among the different methods used to identify the enriched pathways. (**D)** Heatmap of the metabolites identified by NMR. (**E)** Metabolic pathway enrichment by using the NMR identified metabolites and MetaboAnalyst 3.0 program. (**F)** The concentration of some glutamine metabolism-related metabolites in the different samples is represented. Six to nine independent samples from different cell cultures (n = 3) were obtained per sample condition.

The analysis was first performed for each comparison, NF/N and TF/T (S5 Fig), and then on the combination of the two datasets (Fig 2A). The data have been displayed graphically by heatmaps in which the directionality classes are represented as columns and the position of each gene set (ranking) in the aggregated rank is represented as color variation (pathway rankings from 1 to 200). For full list of PIANO aggregate analysis see S5 Table. A pathway has been included in the heatmap if it ranked in the first 10 (for gene dataset and gene + protein datasets combination) or the first 5 (protein data set) positions in at least one directionality class. Comparative analysis between the results represented in Fig 1E, 1F, 1I and 1J and panels A and B in S4 Fig indicated a low percentage of overlapping pathways amongst different approaches. In fact as shown in Fig 2B and 2C, the pathways identified with the three sets of data (highly expressed genes, all expressed genes and proteins, S1, S2 and S3 Tables) overlapped in few cases (all pathways used for Venn diagram are listed in S6 Table). Importantly, among these, both comparisons identified one pathway common to all three types of analysis, namely *Purine metabolism* for NF/N and *Protein processing in ER* for TF/T.

A more detailed analysis of the pathways identified by GSEA (Fig 2A) further underlined the different response between the two cell lines to FSK stimulation. Regarding NF/N down-regulated pathways, several terms were associated with a decrease of protein translation machinery (*Aminoacyl-tRNA biosynthesis*, *Protein export*, *RNA transport* and *Ribosome)* as well as with a general reduction of amino-acid metabolic pathways. Regarding up-regulated pathways, PKA stimulation induced several pathways involved in hexose metabolism (*Glycolysis/Gluconeogenesis*, *Fructose and Mannose metabolism* and *Pentose phosphate pathways)* and nucleotide metabolism (*Purine and Pyrimidine metabolism)*, suggesting the activation of alternative metabolic pathways to avoid glucose starvation effects. As regards TF/T down-regulated pathways as compared to NF/N, we found an equal decrease of protein translation machinery and an opposite regulation of the pathways involved in hexose metabolism. Notably, among the most up-regulated pathways (Fig 2A and panel B in S5 Fig) lipid metabolism (*Synthesis and degradation of ketone bodies*, *Terpenoid backbone biosynthesis* and *Butanoate metabolism*, *Fatty acid degradation* and *Fat digestion and absorption)* appeared highly enriched. Further analysis of PIANO results, either as single data (panels A and B in S5 Fig) or as aggregate data (Fig 2A), showed for both comparisons and especially for TF/T sample a marked enrichment of the catabolic pathways, namely *Proteosome*, *Phagosome*, *Peroxisome* and *Lysosome*, pointing toward the activation in TF cells of these pathways in glucose starvation, probably as temporary survival mechanisms, providing alternative energy sources and general anabolic cell constituents [28,29].

### PKA activation in Transformed cells controls amino acid metabolism and glutamate-linked pathways

To evaluate the effect of PKA activation on intracellular metabolism in Normal and Transformed cells, we performed an NMR-based metabolomics analysis. First, the cells were treated with FSK, as described in Fig 1A, and then a set of ^1^H NMR spectra were acquired from cell extracts of FSK-treated or untreated cells. Analysis of NMR data indicated that Transformed untreated cells had a higher level than Normal cells of almost all the metabolites identified, apart from acetate and acetone (Fig 2D and S7 Table). In particular several amino acids and glutathione (GSH/GSSG) were more concentrated in Transformed cells. Notably, FSK treatment induced an overall increase, especially in Normal cells, of all tested metabolites (see for instance amino-acid levels), hence partially reducing the differences observed in untreated samples. Nevertheless, the NMR data indicated a significant reduction in cellular glutamate level only in Transformed cells, suggesting a change in glutamine/glutamate metabolism. FSK treatment also elevated myo-inositol, uracil and uridine. Of note, the decrease of alternative energy sources such as acetate, acetone and creatine, specifically in glucose-depleted cancer cells (S7 Table), suggests alternative metabolic routes activation to avoid the effect of glucose starvation. To further interpret the biological significance of the metabolite changes in both cell lines, we used MetaboAnalyst tools to connect the metabolites to metabolic pathways. Fig 2E shows the most over-represented metabolic pathways obtained using the 24 metabolites. The results are consistent with a role of PKA activation in metabolic remodeling especially of Transformed cells, since analysis of some metabolites mapping in three pathways strictly interconnected, namely glutamate metabolism, glutathione metabolism, ammonia recycling, indicated several differences between Normal and Transformed cells. Indeed, we observed a strong decrease of glutamate (Fig 2F, left panel) linked to a slight decrease of nicotinamide adenine dinucleotide (NAD), as well as to an increase of glutathione (so more the oxidized form GSSG) and *N*-acetylglucosamine-6-phosphate (GlcNAc-6P) (Fig 2F, right panel). No variations for glycine, glutamine and succinate were observed. Conversely, all these metabolites were significantly increased in NF cells (Fig 2F, left and right panels), further suggesting a different metabolic response between the two cells lines upon PKA activation.

### PKA-mediated decrease of unfolded protein response (UPR) is associated with an increase of protein N-glycosylation levels

**Fig 3:**
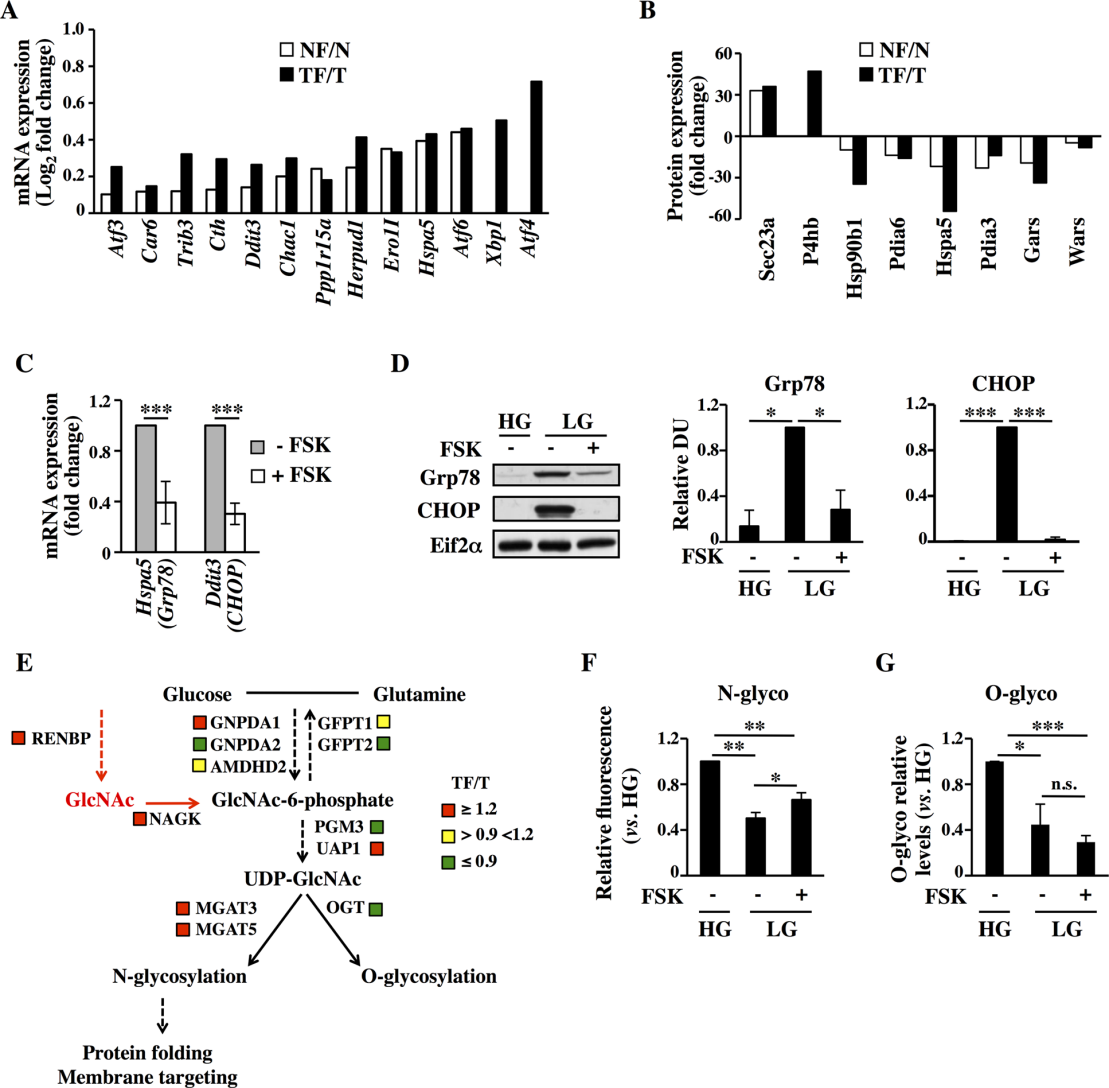
FSK-mediated attenuation of UPR is accompanied by an increase of membrane protein glycosylation. (**A-B)** UPR-related transcriptional data from microarray (A) and proteomic (B) data. (**C-G)** All the analyses are referred to Transformed cells and were performed at 72h (C-E) or 96h (F-G) of culture. (**C)** qPCR analysis of UPR mRNA levels in FSK-treated cells with respect to untreated cells. (**D)** Western blot analysis of UPR-related proteins. (**E)** Schematic representation of the HBP in which the expression levels of some HBP-related mRNAs have been indicated. The data are the ratio TF/T and are represented in color code (red up-regulated; yellow unchanged; green down-regulated). (**F)** FACS analysis of live cells stained with fluorochrome-conjugated ConcanavalinA. (**G)** Western blot analysis using a specific anti O-GlcNAc antibody and densitometric analysis of the film. All data represent the average of different experiments (n>3).

The combination of multi-omics technologies and the data analysis with different enrichment tools indicated the *Protein processing in ER* as common regulated pathway for both Normal and Transformed cell lines upon FSK treatment. In particular, detailed analysis of either the transcriptional and proteomic data enriching this pathway indicated a significant decrease of genes (Fig 3A) and proteins (Fig 3B) involved in a mechanism termed UPR, whose induction has been reported in various cell models following a decrease in energy sources, like glucose, leading either to survival or, if prolonged, to cell death [11,30,31]. Strikingly, almost all the FSK-repressed genes characterizing UPR were previously identified as an UPR gene signature activated under glucose deprivation conditions and cell death [12]. To validate the transcriptional repression of the UPR genes upon FSK treatment, we selected six UPR-related genes (*HSPA5*, *DDIT3*, *XBP1*, *ATF4*, *TRIB3*, *PPP1R15A*) in our transcriptional data, to assess their expression at 72h both in Normal and Transformed cells grown in LG -/+ FSK. Both cell lines showed a decreased expression of almost all these genes (Fig 3C–Transformed cells- and panels A and B in S6 Fig), excluding ATF4 that remained unchanged in Transformed cells (Fig 3A–array data only in Transformed cells- and panels A and B in S6 Fig). Consistently, the ER stress-dependent splicing of X-box binding protein 1 (XBP1) was detectable only in the untreated cells, further confirming the PKA role in the repression of UPR cascade activation (panel C in S6 Fig). Since Normal cells are slightly sensitive to glucose starvation, both in untreated and FSK-treated samples [21], and since we were more interested in cancer cell survival mechanisms regulated by PKA activation, we tested only in Transformed cells UPR activation by Western blot analysis of two UPR hallmarks, the glucose-regulated protein 78 (Grp78 or Hspa5) and the C/EBP-homologous protein (CHOP or Ddit3). As expected, the expression of both proteins was elevated in LG at 72 h when compared to cells grown in 25mM glucose (High Glucose, HG) and was almost completely repressed by FSK treatment (Fig 3D). Collectively, these results indicate that exogenous PKA activation by inhibiting the UPR transcription program could protect specifically Transformed cells from the deleterious effects of glucose starvation.

Glucose starvation also results in decreased flux through the HBP that can lead to diminished protein glycosylation, protein misfolding and ER stress [12,32,33]. Protein glycosylation depends in part on *N*-acetylglucosamine (GlcNAc), a precursor for both *N*- and *O*-linked glycosylation. Indeed, previous findings have shown that exogenous supplementation of GlcNAc induces prolonged survival of glucose-deprived cancer cells by inhibiting UPR activation [12]. Since our metabolic data indicated an increase of the intracellular levels of GlcNAc-6P in FSK-treated samples (Fig 2F, right panel), we first sought to determine if such increase was associated with mRNA expression level changes of HBP genes. We depicted schematically (Fig 3E) the HBP *de novo* protein *N*- and *O*-glycosylation and possible salvage routes for the GlcNAc based on the KEGG amino sugar pathway. As shown in Fig 3E, for Transformed cells the expression levels of genes directly involved in *de novo* synthesis of UDP-GlcNAc did not show a distinct signature, because they were up- and down-regulated or unchanged. However, both *NAGK*, which phosphorylates GlcNAc salvaged from lysosomally degraded glycoconjugate [34], and *RENBP*, which catalyzes the conversion of *N*-Acetyl-D-mannosamine (ManNAc) into GlcNAc participating in the catabolism of sialic acid [35], were up-regulated in FSK-treated cells. Moreover, we observed also the up-regulation of *N*-acetylglucosaminyltransferase, *MGAT3* and *MGAT5*, both of which are able to promote N-glycan protein branching [34]. Conversely, *OGT*, involved in protein *O*-glycosylation, was down-regulated. To validate transcriptional and metabolic data, we assessed Transformed cells at 96h of growth for protein *N*- and *O*-glycosylation, in response to glucose starvation and FSK treatment. As shown in Fig 3F and 3G both modifications, analyzed by Fluorescein isothiocyanate (FITC)-conjugated Concanavalin A and the RL-2 specific antibody respectively, were strongly affected by glucose starvation, since they were almost reduced by 60% as compared to HG optimal growth condition. Notably, PKA activation was associated with a significant increase of protein *N*-glycosylation (Fig 3F). Conversely, no changes in protein *O*-glycosylation were observed upon PKA activation (Fig 3G). Importantly, the higher level of *N*-glycosylation in FSK-treated cells as compared with untreated ones is also suggested by their tight adhesion to cell culture plate and by the strong increase of their spreading area (panel C in S1 Fig). In fact, an increasing body of evidence suggests that N-glycosylation is also important for the cell adhesion [36]. Altogether our data indicated that protein glycosylation levels, strongly dependent on glucose availability, are partially restored by PKA activation through its positive effect on expression of genes encoding proteins involved in GlcNAc salvage and on intracellular level of GlcNAc-6P. This results in higher levels of protein *N*-glycosylation that promote UPR attenuation.

### PKA induces cancer cell survival by activating autophagy under glucose starvation

Nutrient deprivation is one of the severe conditions that an organism as well as a cell may face. To sustain cellular metabolism, lipids, proteins, polysaccharides have to be degraded to give rise for instance to fatty acids, amino acids and oligosaccharides that can in turn generate building blocks and adenosine triphosphate (ATP) to sustain biosynthesis [37]. Prolonged starvation, indeed, may stimulate several catabolic processes like proteasomal degradation, lysosome activation and autophagy. Previous pathways analysis revealed enrichment for genes and proteins involved in *Peroxisome*, *Lysosome*, *Phagosome* and *Proteasome*, suggesting that PKA activation enhanced catabolic processes under glucose starvation (Fig 2 and S5 Fig). In line with this notion, for instance, it has been shown that cAMP is a key effector in the receptor-mediated acidification of lysosomes [38], and that PKA-mediated phosphorylation of proteasome subunits or ubiquitin-ligases may enhance Ubiquitin/Proteasome Pathway activity [39]. Contrasting results have been obtained regarding the potential role of PKA in autophagy activation. In fact, previous studies have indicated that cAMP increase upon glucagon stimulation may induce hepatocytic autophagy *in vivo* and in perfused livers. However, in freshly isolated hepatocytes, autophagy may be stimulated, inhibited or unaffected by cAMP, depending on the external metabolic conditions [40].

**Fig 4:**
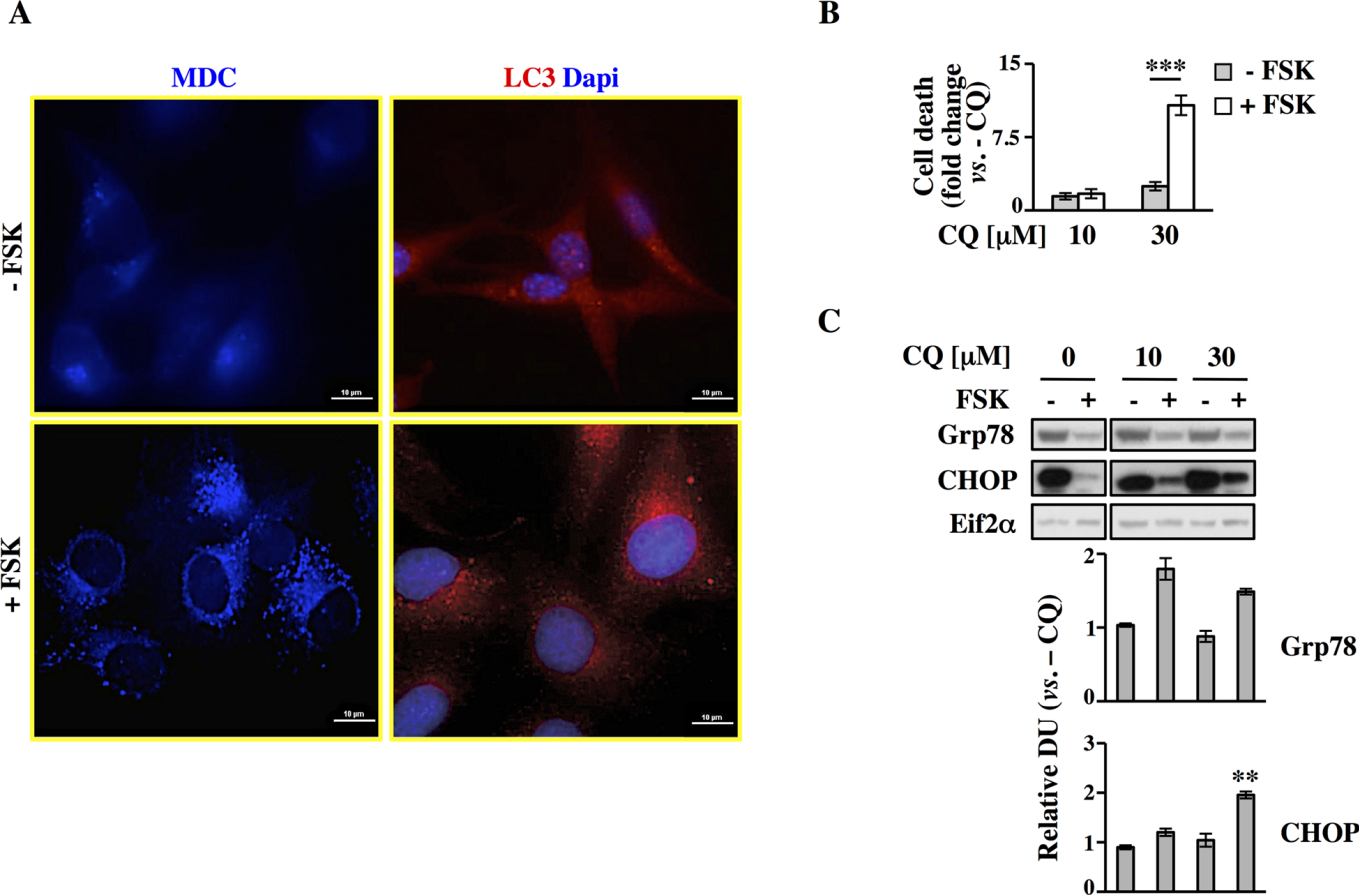
PKA pathway induction results in autophagy activation in Transformed cells. All analyses were performed in Transformed cells cultured for 96h in LG, -/+ FSK. (**A)** MDC staining was performed in viable cells, while fixed cells were stained with a specific antibody against LC3. The images were visualized at 60X magnification. Scale bar 10μm. (**B)** Trypan blue exclusion assay was performed in cells -/+ FSK, treated or not with CQ at the indicated concentration for the last 24h of culture. Data are plotted as fold change over the equivalent control sample (- CQ). (**C)** Expression level of Grp78 and CHOP proteins was analyzed by Western blot in cells -/+ FSK and -/+ CQ with densitometric values. Eif2α expression was used as normalization. All data represent the average of different experiments (n>3).

To determine autophagy induction, we monitored the level of autophagosome accumulation in Transformed cells at 96h, consisting of 24h of glucose lack, by using the autofluorescent monodansylcadaverine (MDC) and following subcellular distribution of LC3 protein, both capable of staining autophagy vacuoles *in vivo* [41,42]. By fluorescence microscopy we observed a diffuse cytosolic staining in untreated cells for both markers (Fig 4A, upper panels). Conversely, upon FSK treatment, the number of dotted positive cells for MDC and LC3 increased, indicating autophagosomes formation and LC3 association with them (Fig 4A, lower panels). Autophagy activation was also monitored by Western blot analysis of Beclin1, another autophagy marker, and LC3 protein cleavage. As shown in the panels C and D of S7 Fig both Beclin1 and LC3 cleavage, measured as LC3-II level, increased significantly in FSK-treated cells.

To test our hypothesis that PKA activation leading to autophagy response could help cells to survive under glucose starvation by attenuating UPR activation, we next investigated the autophagy role in FSK-elicited survival of Transformed cells. Both untreated and FSK-treated cells were treated for 24h (panel A in S1 Fig, experimental scheme) with two dosages of the autophagy inhibitor chloroquine (CQ), 10 and 30μM. Fig 4B shows cell death as trypan blue staining. Treatment with 30μM CQ induced an almost tenfold increase of cellular death in the FSK-treated sample. In contrast, the untreated cells, under these conditions, experienced only around a threefold increase in cell death (Fig 4B). These findings suggest that the treated Transformed cells depend more on autophagy than the untreated ones. On the contrary the latter observed result indicated that untreated cells under glucose starvation do not show a relevant reliance on autophagy, since they are less sensitive to CQ treatment, which in part confirmed previous results indicating that glucose deprivation promotes a low autophagic flux in several cell lines [43].

ER stress and autophagy are strictly correlated since UPR activation induces a series of events that mitigate this burden among which the extreme ER expansion, through the use of ER-derived membranes for the formation of autophagosomes [44] and the excessive accumulation of unfolded proteins [45]. In addition, UPR-induced activation of activating transcription factor 4 (ATF4) has been shown to up-regulate the autophagy genes to promote cell survival during acute hypoxic stress or under 2-Deoxy-Glucose growth [46]. Additionally, autophagy has been shown to be required for the maintenance and survival of oncogenic K-Ras expressing cancer cells and tumors [47,48]. In order to identify a causal relation between FSK-dependent cell survival upon glucose starvation with autophagy activation and UPR attenuation, we measured UPR activation by Western blot analysis of Grp78 and CHOP proteins upon co-treatment with CQ. Inhibition of autophagy by increasing dose of CQ abolished the repressive effect of FSK especially on CHOP protein expression (Fig 4C). To further test whether PKA was definitely required to activate autophagy, we added 10μM H89 (N-[2-p-bromocinnamylamino)ethyl]-5-isoquinolinesulfonamide dihydrochloride), the most effective selective inhibitor of PKA [49,50] together with FSK. As shown in panel A in S7 Fig, daily co-treatment with H89 decreased PKA activation, as measured by Western blot analysis of PKA specific substrate and phosho-CREB levels, and the survival of FSK-treated and untreated cells as well (panel B in S7 Fig). To avoid H89-dependent excessive cell death, autophagy experiments were performed upon a single treatment of 9h with 10μM H89, starting from 72h of cell culture. Interestingly, after H89 treatment, LC3-II level decreased at almost the same value as observed in untreated cells (panel D right in S7 Fig). In addition, analysis of MDC staining at the same time-point (panel E in S7 Fig) indicated that PKA inhibition strongly affected autophagic vacuole formation since the staining appeared cytoplasmic diffuse like in untreated cells. These results demonstrate that PKA induces autophagy, leading to UPR attenuation, since it could be abolished by co-treatment of cells with the selective PKA inhibitor H89. Moreover, they suggest that endogenous PKA activation has a role in untreated Transformed cell survival under chronic glucose starvation as well, since H89 alone increased basal cell death (panel B in S7 Fig).

### PKA activation stimulates glutamine metabolism: an essential response to survive under glucose starvation

Metabolic analysis and specific enriched pathways obtained by using the identified metabolites indicated an association between glutamate metabolism and PKA activation (Fig 2E). In addition, as previously observed in other cancer cells, Transformed cells showed at least an eightfold higher intracellular glutamate concentration when compared to Normal cells (S7 Table). However upon PKA activation, while in Normal cells we observed a two-fold increase of glutamate concentration, in Transformed cells such a value decreased by around 30%. Normally, variation of intracellular glutamate concentration is derived primarily from high uptake of glutamine and an enhanced glutaminolysis [51]. Then, intracellular glutamate may be converted into trycarboxylic acid (TCA) cycle intermediates, used for glutathione biosynthesis or as exchange substrate for L-cystine [51]. These three metabolic routes participate in the glutamate changes. Otherwise, several authors have shown that cancer cells upon glucose withdrawal may become much more glutamine-dependent for their survival [52,53].

**Fig 5:**
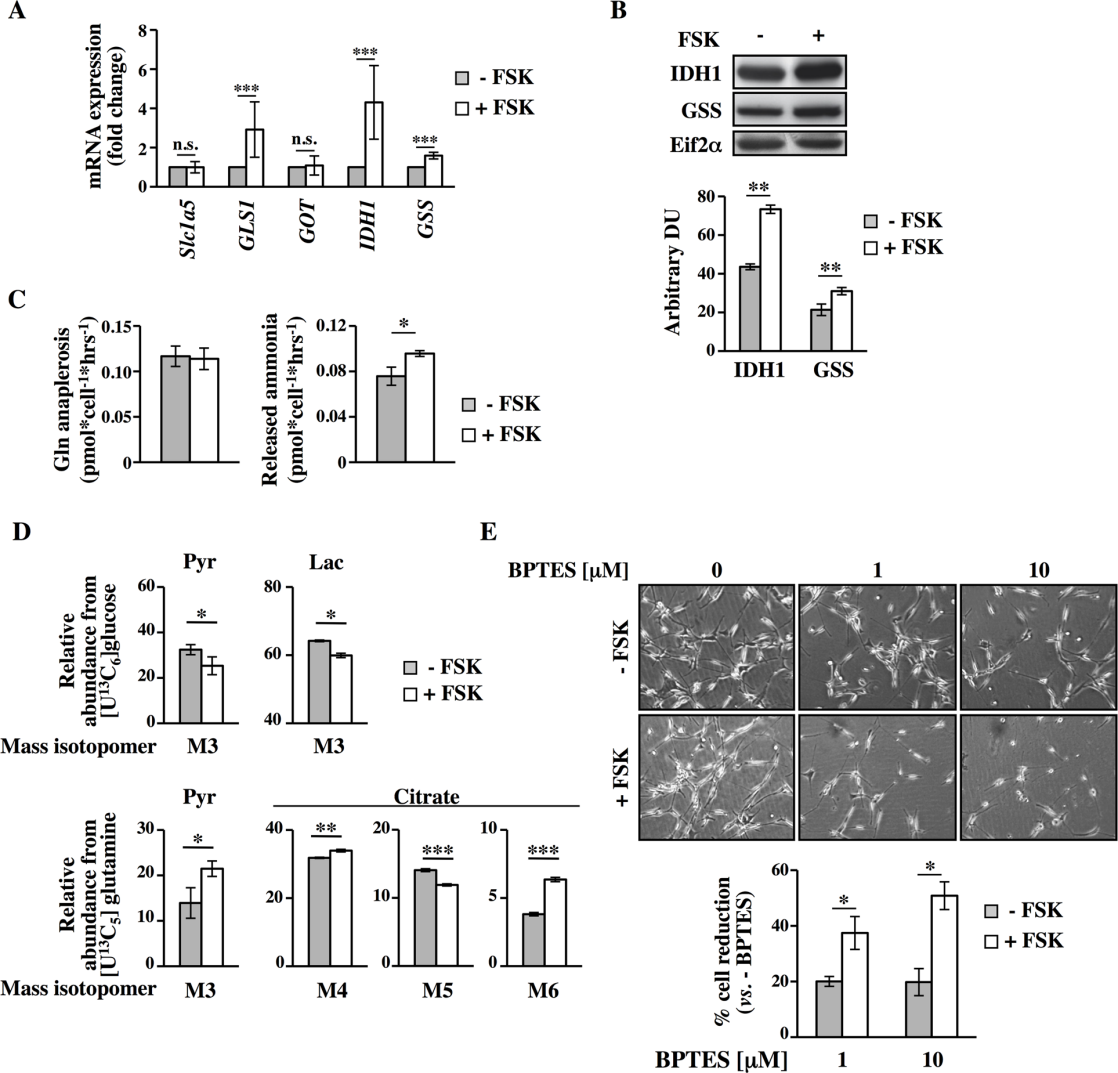
FSK-mediated survival in glucose depletion is also dependent on glutamine metabolism. (**A-B)** qPCR (A) and Western blot (B) analyses were performed in Transformed cells -/+ FSK at 72h of culture. (**C)** Glutamine, glutamate and ammonia levels were analyzed in culture media of Transformed cells. Glutamine anaplerosis was determined based on glutamine uptake and glutamate secretion. Both glutamine anaplerosis and the released ammonia were calculated as pmol*cell^-1^*h^-1^. (**D**) MIDs of target metabolites from [U^13^C_6_]glucose and [U^13^C_5_]glutamine were evaluated in Transformed cells at 80h of culture. (**E)** -/+ FSK cells co-treated or not with BPTES at the indicated concentrations for 24h, were counted at 96h of culture. Also representative images of different conditions are presented. All data represent the average of different experiments (n≥3).

In order to investigate whether PKA could modulate the main glutamine/glutamate metabolic routes, we analyzed the changes of some mRNA and proteins directly involved in these pathways. First of all we searched in *Affymetrix* data the genes involved in these metabolic pathways. As shown in S8 Fig, we identified several differentially expressed genes involved in glutamine metabolism. In order to better specify the transcriptional data we divided the genes into four different functional classes namely glutamine/glutamate transport, glutaminolysis, reductive carboxylation and glutathione biosynthesis. This analysis indicated a strong repression of the genes encoding for the transporters (indicated as glutamine/glutamate transport). Regarding glutaminolysis, we observed an increase of *Glutaminase 1* (*GLS1*), and the tricarboxylic acid cycle enzymes downstream of α–Ketoglutarate formation, namely *OGDH*, *SDHA*, *SDHB*, *SDHC* and *SDHD*. Transaminases appeared generally more repressed since two out of three identified were strongly decreased. With regard to reductive carboxylation, the two main enzymes involved in this process, namely *IDH1* and *ACLY*, were up-regulated. Importantly *ACLY*, central also for fatty acid synthesis, was found up-regulated also in proteomic data (Fig 1I). Finally an up-regulation was observed also for *GSS* and *GSR* directly involved in glutathione biosynthesis and recycling, respectively. To validate the expression data, we analyzed the changes in some of them in terms of mRNA and protein expression at 72h. As shown in Fig 5A, quantitative real time PCR (qPCR) analysis confirmed the FSK-dependent increase of *GLS1*, *IDH1* and *GSS*. No significant changes in *Slc1a5* and *GOT* expression were observed. For *GSS* and *IDH1*, such an increase was also confirmed by Western blot analysis (Fig 5B).

To test whether the modulating effect of PKA on genes/proteins of glutamine metabolism could also change the rate of glutamine uptake and consumption and its metabolism, we measured glutamine uptake as well as glutamate and ammonia secretion into the culture medium. While glutamine anaplerosis into the TCA cycle was unchanged in FSK-treated cells as compared to untreated, ammonia secretion was slightly but significantly higher in FSK-treated cells. This suggests an increased TCA-independent glutamate utilization and may account for the increased GSH/GSSG synthesis in FSK treated cells (Fig 2F). To confirm and further delineate the FSK-mediated change of glutamine metabolism, we performed stable isotope assisted metabolic profiling upon supply of [U^13^C_6_]glucose and [U^13^C_5_]glutamine as tracers to the cells. Mass isotopomer distributions (MIDs) of intracellular metabolites were analyzed at 80h of culture, when both–FSK and +FSK cells have been in a condition of glucose lack for several hours [21]. Worthy of note, while the contribution of glucose derived carbon to pyruvate and lactate decreased in +FSK cells as compared to untreated cells, it was substituted by glutamine carbon derived via the cataplerotic flux of glutaminolysis. In agreement, we observed an increased contribution of glutamine carbon in pyruvate and citrate. Increased fraction of M3 pyruvate indicates an increased flux through malic enzyme. In agreement, we observed an increased activity of oxidative TCA metabolism (M4 and M6 citrate), while reductive carboxylation was decreased (M5 citrate) (Fig 5D). Altogether these data support the hypothesis that FSK-treated cells, as compared to untreated controls, adjust their metabolism to increase glutamine oxidation into TCA cycle as well as an increased glutamine utilization for glutathione synthesis. Next, we validated the importance of this metabolic route in the survival effect induced by FSK in glucose starvation by using a specific glutaminase inhibitor namely bis-2-(5-phenylacetamido-1,2,4-thiadiazol-2-yl)ethylsulfide (BPTES) [54] (panel A in S1 Fig for the experimental scheme). BPTES treatment, at 1 and 10μM for 24h, first induced a slower growth and then cell death preferentially in FSK-treated cells (Fig 5E). Collectively, these data indicate that PKA activation regulates Transformed cell survival under glucose starvation by activating autophagy and regulating glutamine metabolism that concurrently lead to cell survival in absence of glucose.

### PKA specific regulation of UPR and autophagy protects human MDA-MB-231 cancer cells from the cell death induced by glucose starvation

**Fig 6:**
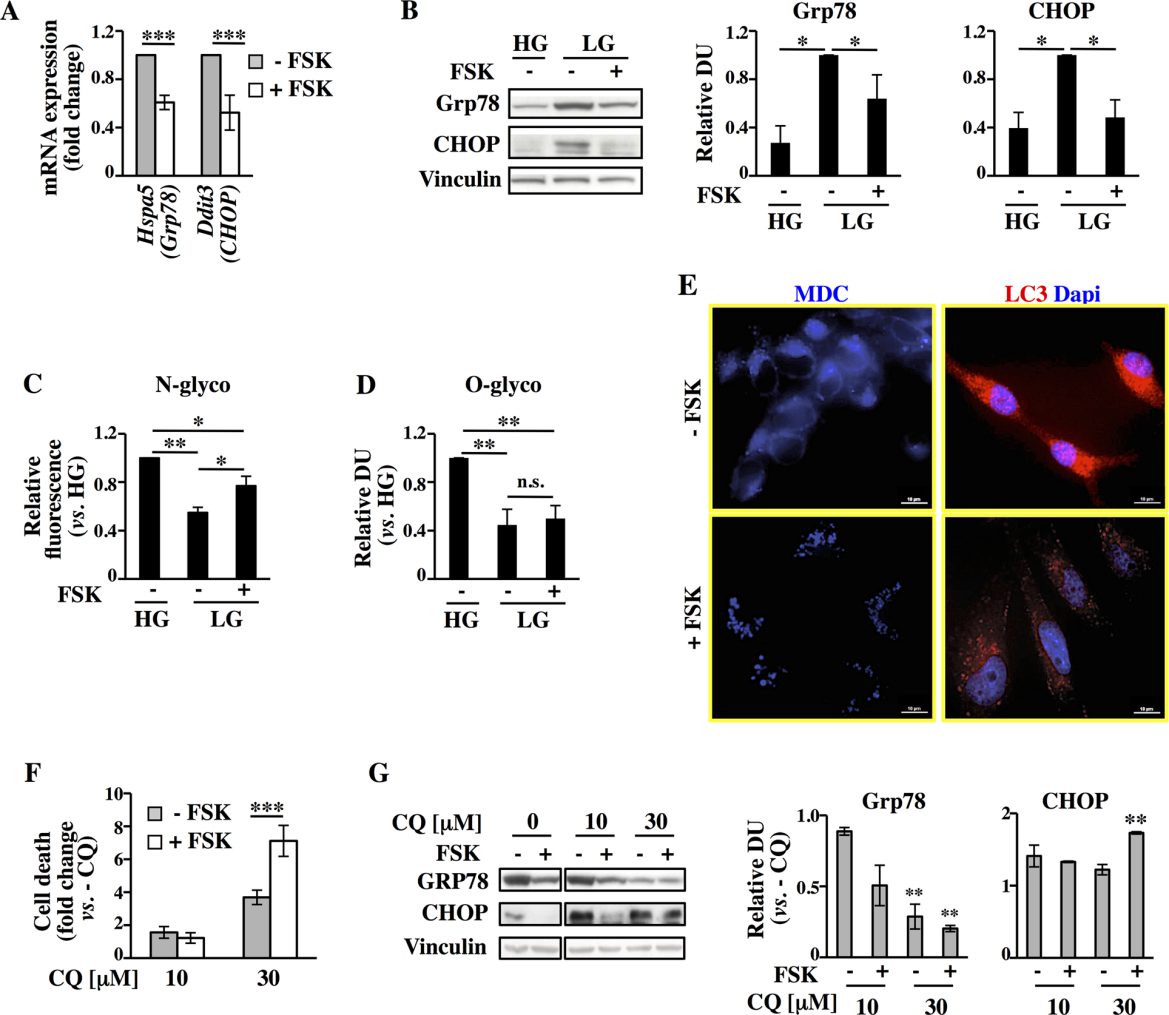
PKA pathway activation in MDA-MB-231 cells promotes UPR attenuation and autophagy enhancement. All the analyses were performed in MDA-MB-231 cells cultured in LG -/+ FSK. (**A-B)** mRNA and protein levels of Grp78 and CHOP were analyzed by qPCR (A) and Western blot (B) at 48h of culture. (**C-D)** N-glycosylation level (C) and O-glycosylation level (D) were analyzed at 72h of culture (see Fig 3F–3G). (**E)** MDC labeling and LC3B antibody staining at 72h of culture. (**F)** Trypan blue exclusion assay (see Fig 4B). (**G)** Grp78 and CHOP proteins expression level of cells -/+ FSK and -/+ CQ. Vinculin expression was used as normalization.

In order to evaluate whether the mechanisms previously described for Transformed cells were also functioning in glucose-addicted human cancer cell lines, we evaluated UPR and autophagy activation in the breast cancer cells MDA-MB-231. According to our published results this cell line is strongly sensitive to glucose deprivation-induced apoptosis and responsive to FSK treatment, which leads to their survival [21]. In fact, as shown in panels E and F in S1 Fig, in association with FSK-dependent enhancement of PKA activation (panel E in S1 Fig), as determined by Western blot (p-PKAs and pCREB S133) and by ELISA assay, an enhanced cell survival was observed (panel F in S1 Fig). Next, in order to determine the effect of FSK on UPR also in these human cancer cells, we evaluated the mRNA and protein expression levels of Grp78 and CHOP after 48h of growth, when cells are in complete absence of glucose [21]. As shown in Fig 6A and 6B, a decrease in mRNAs and protein expression was observed for both, especially for CHOP, following FSK treatment when compared to untreated cells. In addition, analysis of *N*- and *O*-protein glycosylation at later time point (72h) (Fig 6C and 6D) indicated for both modifications a glucose-dependent decrease. Indeed both values obtained for the LG were reduced by around 60% when compared to HG growth. Worthy of attention is the fact that also in MDA-MB-231 cells, PKA activation was associated with a significant increase of protein *N*-glycosylation (Fig 6C).

Consistent with the results obtained for the Transformed cells, the analysis of autophagy in MDA-MB-231 breast cancer cells by MDC labeling (Fig 6E, left panels) and LC3 localization (Fig 6E, right panels) indicated a FSK-dependent appearance of punctate vacuoles resembling autophagosomes. Such activation was also confirmed by Western blot analysis of LC3 protein cleavage (panel D left in S9 Fig). To validate also in MDA-MB-231 cells the protective effect of PKA activation through autophagy, we next measured the outcome of 24h treatment with CQ (panel D in S1 Fig, experimental scheme) in terms of survival in untreated and FSK-treated cells. CQ induced a significant increase of cell death especially in treated samples (Fig 6F). Accordingly, the pro-death UPR marker CHOP showed higher expression in CQ treated cells (Fig 6G). Notably, the same samples showed a decreased expression of the pro-survival Grp78 protein (Fig 6G) further confirming a link between autophagy activation and UPR inhibition in FSK-treated sample. Next, we sought to determine whether H89, in co-treatment with FSK, could reduce cell survival and autophagy also in MDA-MB-231 cells. As shown in S9 Fig, in MDA-MB-231 cells, H89 co-treatment, in association with its inhibitory effect on PKA activity (panel A in S9 Fig, p-PKAs and pCREB S133) increased either FSK-dependent or FSK-independent cell death (panel B in S9 Fig). Notably such a reduction of cell survival was associated to higher expression of UPR markers, Grp78 and CHOP (panel C in S9 Fig), to a reduced expression and conversion of LC3B-I protein (panel D in S9 Fig) and to a strong reduction of autophagy vacuoles (panel E in S9 Fig). Importantly, cells treated with only H89 showed similar phenotypes, suggesting that also in non-stimulated cancer cells PKA has a role in survival under chronic glucose starvation (panels B, C, D and E in S9 Fig). To strengthen the conclusion that a cAMP-PKA signal mediates the described results, we examined the effect of the knockdown of the gene encoding PKA catalytic α subunit (PKAcat α or PKAc) (panel F in S9 Fig). Similar results to H89 treatment were observed following targeted knockdown of the PKAc. In fact, knockdown of PKAc led to a reversal of FSK-stimulated survival almost back to control levels (panel G in S9 Fig). Importantly, such a reversion was also associated to a significant effect on the basal activation and on the FSK-dependent increase of the autophagy marker LC3B (panel H in S9 Fig).

Since cAMP stimulates different downstream effectors, including PKA and Exchange Protein directly Activated by cAMP (Epac)-1/2 [55], which both may be involved in the observed FSK-dependent effects, we decided to determine the role of Epac by studying cell survival, autophagy and UPR in the presence of the newly reported inhibitor of Epac activation, ESI-09 (panel A in S10 Fig) [56,57]. As shown in S10 Fig, the daily co-treatment of the cells with FSK and ESI-09 had little impact on FSK-dependent cell survival (panel B in S10 Fig), autophagy vacuoles formation (panel C in S10 Fig) and UPR inhibition (panel D in S10 Fig). Together these results provide direct support for an important role of cAMP-PKA signal to promote the cancer cell survival under glucose starvation.

### Glutamine metabolism participates to MDA-MB-231 cell survival upon PKA activation

Previous data indicated that the activation of the PKA pathway was linked to glutamine dependency of glucose starved Transformed fibroblasts. To investigate this effect also in MDA-MB-231 cancer cells, we first evaluated the expression of some genes related to glutamine utilization. As shown in S11 Fig, qPCR analysis performed at two different time points, 48h and 72h, indicated an up-regulation of *Slc1a5*, *GOT1* and *IDH1* at 72h in FSK treated samples, hence confirming also in human breast cancer cells a role of PKA activation in glutamine metabolism.

**Fig 7:**
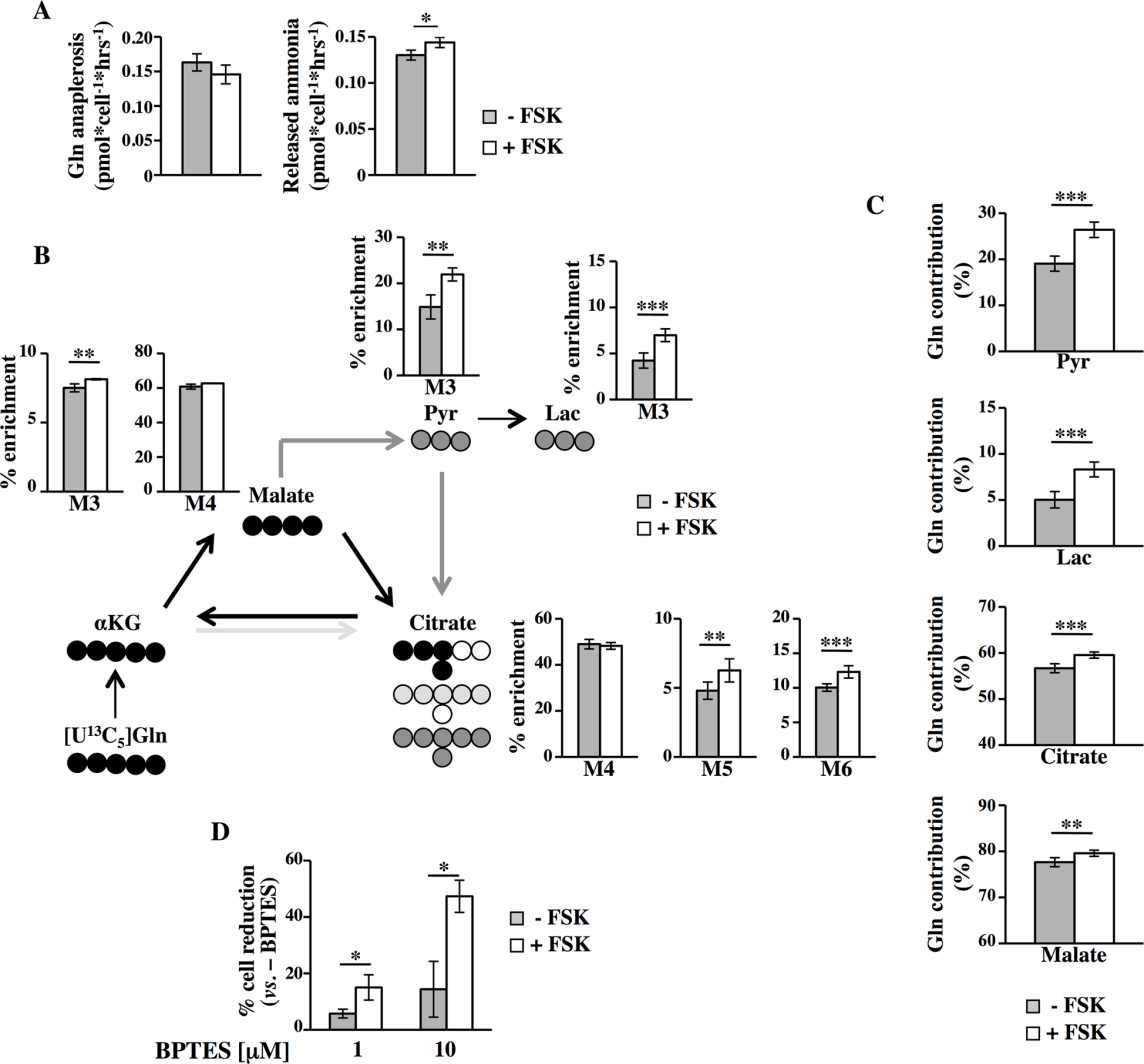
FSK mediates a change in glutamine metabolism in human MDA-MB-231 cancer cells. (**A)** Glutamine, glutamate and ammonia levels were analyzed in culture media of MDA-MB-231 cells. Glutamine anaplerosis was determined based on glutamine uptake and glutamate secretion. Both glutamine anaplerosis and the released ammonia were calculated as pmol*cell^-1^*h^-1^. (**B-C**) MIDs of target metabolites from [U^13^C_5_]glutamine were evaluated in MDA-MB-231 cells after 56h of culture. Fraction of glutamine derived isotopologues (B) and contribution of [U^13^C_5_]glutamine to target metabolites (C) were determined. In panel B, x-axis represents the mass isotopomer, while the y-axis indicates the relative abundance from [U^13^C_5_]glutamine. (**D**) -/+ FSK cells were treated with BPTES for 24h and counted at 72h of culture. Percentage of reduction after the treatment is shown. All data represent the average of different experiments (n≥3).

To further support gene expression data, we determined the effect of PKA activity on glutamine anaplerosis and ammonia release (Fig 7A). FSK-treated cells, similarly to transformed mouse fibroblasts, showed an unchanged glutamine anaplerosis into the TCA cycle (Fig 7A, left panel) associated to a slight but significant higher level of ammonia release (Fig 7A, right panel). To determine the metabolic transformation of glutamine carbon in MDA-MB-231 cells, we incubated the cells in the presence of [U^13^C_5_]glutamine (Fig 7B). Similar to Transformed fibroblasts, FSK-treated cells showed higher levels of M3 pyruvate and M3 lactate as well as M6 labeled citrate, indicating again an increased glutaminolysis. In contrast to Transformed fibroblasts, however, FSK treatment induced in MDA-MB-231 cells a significant increase of M5 citrate as well as M3 malate isotopologues, both indicative for reductive glutamine carboxylation by IDH. As shown in Fig 7C, the fractional contribution of glutamine carbon to pyruvate and lactate as well as to the TCA metabolites citrate and malate increased in MDA-MB-231 FSK-treated cells. In light of these results, we investigated whether glutamine was also in these cells involved in FSK-dependent survival and proliferation by BPTES (panel D in S1 Fig, experimental scheme). As shown in Fig 7D, BPTES treatment, consistent with the results in Transformed fibroblasts, resulted in a significant dose-dependent decrease of proliferation especially in FSK-treated cells. Altogether these findings demonstrate that PKA activation plays a major role in MDA-MB-231 cell survival upon glucose starvation through autophagy activation and glutamine metabolism regulation.

### Endogenous PKA activation under glucose starvation protects cancer cells from *anoikis*

**Fig 8:**
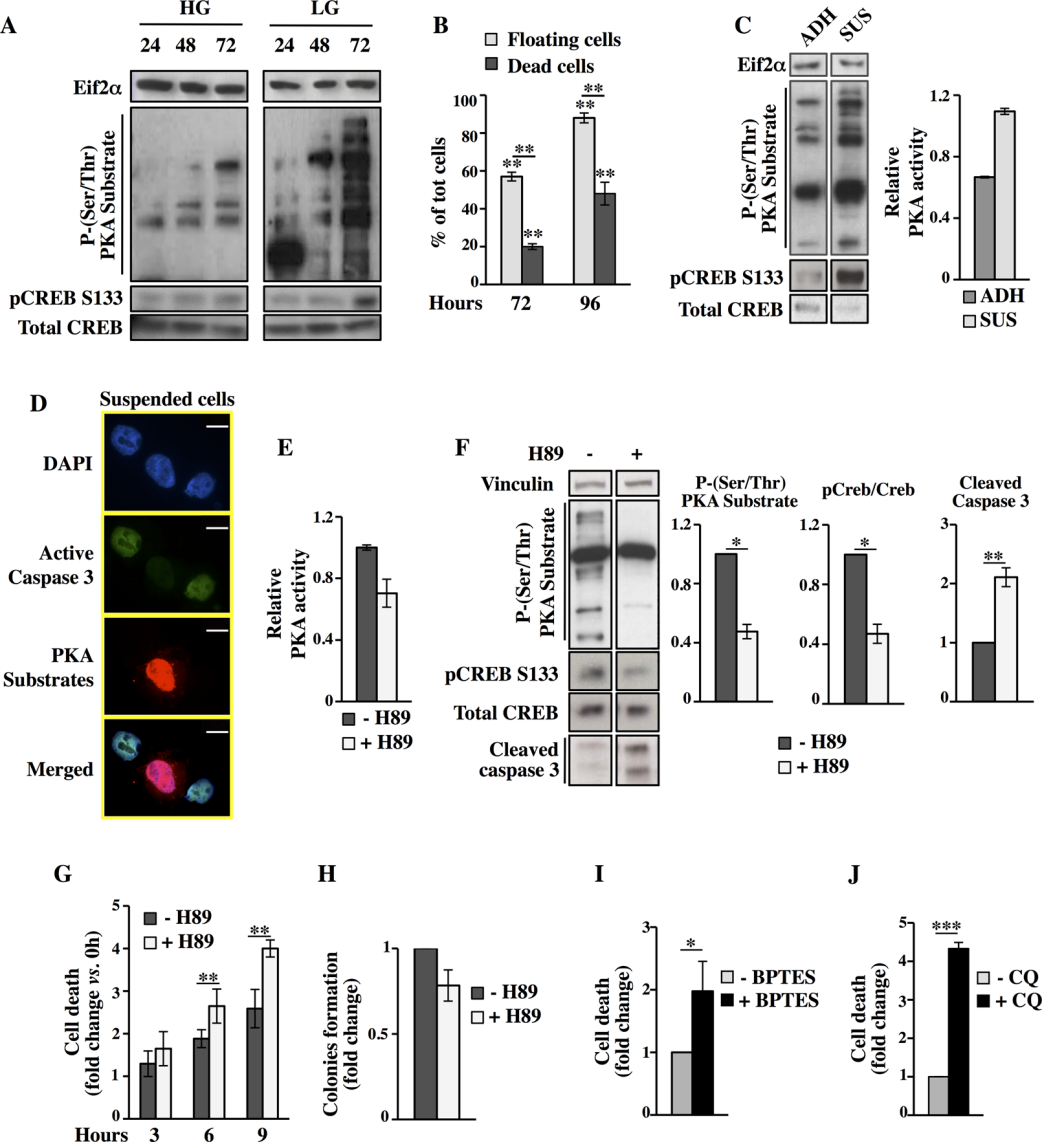
MDA-MB-231 cells cultivation in LG results in a strong endogenous activation of PKA pathway with pro-survival effects. (**A)** PKA time-dependent activation in HG and LG was evaluated by Western blot analysis of p-(Ser/Thr) PKA substrates and pCREB S133. (**B)** Percentage of floating and dead cells at indicated time points of culture in LG. (**C)** At 72h of culture in LG suspended and adherent cells were separately collected to analyze PKA activity by Western blot as well as by ELISA assay. (**D-J)** All analyses were performed on floating cells collected at 72h of culture in LG. **(D)** Co-staining with a fluorescent substrate for active caspase 3, a p-PKAs antibody and DAPI. **(E)** PKA activity was determined by ELISA assay in cells treated or not treated with 10μM H89 for 6h. (**F)** Western blot analysis of pCREB S133 and p-(Ser/Thr) PKA substrates as well as of active caspase 3 in cells treated or not with H89. (**G**) Trypan blue exclusion assay was performed in cells treated or not treated with 10μM H89. (**H)** Colony formation assay was performed with cells treated or not with H89 for 6h. The colony formation was evaluated after 14 days of culture in HG as crystal violet absorbance. (**I-J)** Viable count by trypan blue was performed in cells treated with 10μM BPTES (I) or 10μM CQ (J) for 6h. All data represent the average of different experiments (n = 3).

Cancer cells have developed different mechanisms to survive in stress conditions. Among these stresses, glucose deprivation has been widely studied but not yet well characterized in terms of survival response. Given the critical role of PKA pathway, upon exogenous stimulation, in causing cell survival under glucose starvation, we sought to determine if this pathway could be endogenously activated in MDA-MB-231 cancer cells to promote survival under glucose starvation. To monitor PKA activation, we used the antibodies able to detect p-PKAs and pCREB S133. As shown in Fig 8A, glucose depletion resulted in a very significant induction of p-PKAs and pCREB S133. Indeed at 72h, time corresponding to 24h of growth in complete absence of glucose we observed a two and fourfold increase of PKA activity as compared to 48h in LG and 72h in HG, respectively. Interestingly, microscopic morphological analysis performed at the same time point (72h), indicated that around 60% of the cell population in the plate was floating, becoming around 90% at 96h (Fig 8B and panel F in S1 Fig). However, trypan blue staining of these floating cells indicated that they were in part alive, in particular for 65% at 72h and for 45% at 96h (Fig 8B), suggesting an ability of a significant part of MDA-MB-231 cells to survive up to 48h to glucose starvation and matrix detachment.

Previous data have shown that glucose deprivation or matrix detachment may accelerate cancer cell death activating apoptosis, necrosis and *anoikis* [3]. Moreover, a protective role of some signaling pathways, among which AMPK, in both stress conditions, by saving intracellular ATP and Nicotinamide adenine dinucleotide phosphate (NADPH) levels as well as inhibiting protein synthesis and favoring autophagy, has been also shown [17,58,59]. Since our results indicated that PKA activation had an effect on autophagy and cell metabolism and since such activation was strongly dependent on glucose availability, we wondered if PKA could act as a novel pathway involved in cancer cell survival under conditions of energy stress and cell detachment, avoiding *anoikis*. First, we evaluated the PKA activation by Western blot using p-PKAs antibody and pCREB Serine 133 in both adherent and suspended cells. Strikingly, PKA activation was stronger in suspended cells when compared to adherent ones (Fig 8C). Similar results were obtained also in Transformed cells. In fact, as shown in panel C of S1 Fig and in panel A of S13 Fig, also in these cells was observed the appearance of a mixed population of floating cells of which almost 50% were alive as well. Notably, also suspended Transformed cells were characterized by a higher level of PKA activation (panel B in S13 Fig). Since our previous results proposed a protective role of PKA and given that suspended cells were a mixed population of alive and death cells, we sought to determine if there was a relation between PKA activation and alive cells resisting to *anoikis*. Accordingly, we evaluated the co-localization of p-PKAs signal and active caspase 3 by fluorescence microscopy at 96h in suspended MDA-MB-231 cells. As shown in Fig 8D, PKA activation strongly correlated with *anoikis*-resistance, as demonstrated by the fact that highly positive active caspase 3 cells (green) had low level of PKA activation (red); vice versa highly positive PKA activation cells (red) had low level of active caspase-3. To exclude whether caspase-3 might be simply a consequence of the labeling procedure, we evaluated this staining by using FSK-treated cells grown in LG, first detached and then treated as suspended cells. As shown in panel A in S14 Fig, FSK strongly stimulated PKA signaling, as expected, with no induction of active caspase 3. Deregulation of *anoikis*, such as *anoikis*-resistance, is a critical mechanism in tumor metastasis. Oncogenes including *Ras*, phosphatidylinositol 3-kinase (PI3K)/ protein kinase B (Akt), integrin-linked kinase, and focal adhesion kinase are important regulators of *anoikis*-resistance. More recently, cancer cells metabolic deregulations are becoming an important determinant in *anoikis*-resistance [60]. However, the molecular mechanisms by which tumor cells escape *anoikis* are still poorly understood. To further examine and confirm the role of PKA in *anoikis-*resistance, the suspended cells were collected at 72h and treated for 3, 6 and 9h with 10μM H89 (see panel A in S12 Fig for the experimental scheme). The inhibitory effect of H89 on PKA was evaluated upon 6h of treatment by measuring the PKA activity (Fig 8E), p-PKAs and pCREB S133 (Fig 8F). At the same time point was also evaluated the *anoikis* activation, through the measurement of caspase 3 (Fig 8F). As shown in Fig 8F, H89 treatment significantly affected PKA activity and enhanced caspase 3 activation. The specificity of the effect was also confirmed by the time-dependent effect on cell viability of suspended breast cancer cells as demonstrated by trypan blue staining (Fig 8G). PKA-dependent cell death were confirmed also by colony formation assay and by measuring autophagy activation using suspended cells treated for 6h with H89. In fact treated cells exhibited a reduced colony formation (Fig 8H) and a reduced LC3 expression (panel B in S14 Fig). To further reinforce the PKA function in cancer cell resistance to *anoikis*, we evaluated cell survival by trypan blue staining and colony assay upon treatment with the PKA inhibitor (14–22) amide (PKI amide, 150μM) that, like the R subunits, mimics the protein substrate providing a pseudophosphorylation site and functions as a competitive inhibitor by binding to the catalytic site of PKA [61] as well as upon silencing of PKAc or using the Epac inhibitor ESI-09 (S12A Fig experimental scheme). As shown in panels B and C of S12 Fig, the PKI amide treatment, as observed upon H89 treatment, increased cell death and reduced the ability to form colonies. Strikingly, the lesser effect of PKI amide as compared to H89 was strictly associated to its poorer ability to inhibit PKA activity (panel D in S12 Fig). In fact, the PKAc silenced cells, displaying a stronger reduction of PKA activity (panel F in S9 Fig), showed a more reduced ability to overcome *anoikis* induced by glucose depletion (panel E in S12 Fig). Importantly, when suspended cells were treated with 1μM ESI-09 (Epac inhibitor), there was no effect on basal cell death, indicating that PKA activity plays the major role in *anoikis*-resistance (panel F in S12 Fig). In order to define also the role of glutamine metabolism as well as autophagy in such PKA-dependent survival of suspended cells, we treated suspended cells with 10μM BPTES or CQ for 6h. As shown in Fig 8I and 8J, both treatments reduced cell survival, suggesting a role of either glutamine metabolism or autophagy in avoiding *anoikis*. To further support our hypothesis, suspended Transformed cells were analyzed upon H89 treatment in terms of cell death, colony formation and autophagy activation (see panel A in S12 Fig for the experimental scheme). As shown in panels C, D and E in S13 Fig, the assays confirmed the capacity of the PKA inhibitor to increase *anoikis*, to reduce colony formation ability as well as LC3B-I protein conversion. In addition, 6h of BPTES or CQ treatments in suspended cells strongly increased cell death (panels F and G in S13 Fig). Altogether these findings suggest that PKA activation contributes to selection of glucose depletion- and *anoikis*-resistant mouse and human cancer cells.

### PKA activation under glucose starvation protects also pancreatic cancer cells from cell death

To reinforce and validate our findings regarding the main role of PKA pathway in cancer cell survival under chronic glucose starvation, we decided to extend our analysis to a pancreatic cancer cell line, MIA PaCa-2. According to our published results and literature data also this cell line was sensitive to glucose deprivation-induced cell death [21,62] and responsive to FSK treatment, which led to their survival (panel A in S15 Fig and [21]) in association with PKA activation (panel B in S15 Fig). In addition, PKA activation induced UPR attenuation (panel C in S15 Fig) and autophagy (panel D in S15 Fig). Inhibition of the latter, by using CQ, strongly affected both FSK-dependent survival (panel E in S15 Fig) and UPR modulation (panel F in S15 Fig). To investigate also the role of glutamine metabolism in MIA PaCa-2 cells, we first evaluated the mRNA expression of GLS1. As shown in panel G of S15 Fig, qPCR analysis performed at 72h indicated its up-regulation in FSK treated sample as compared to untreated. Importantly also these cells were more sensitive to BPTES treatment upon FSK treatment, hence indicating an important role of glutamine metabolism activation in PKA-dependent survival upon glucose starvation (panel H of S15 Fig). Finally, we evaluated also the effect of PKA inhibition on their resistance to *anoikis* under glucose depletion. While for MIA PaCa-2 cells have been shown that loss of cell-extracellular matrix (ECM) attachment induced limited *anoikis* [63,64], in our experimental condition, characterized by complete glucose depletion from 72h of cell growth (panel A of S16 Fig), basal and glucose dependent cell detachment (panel B in S16 Fig) was associated with almost full population cell death (panel B in S16 Fig). Remarkably, MIA PaCa-2 cells showed an adequate ability to avoid the glucose-dependent *anoikis* when were cultured from the beginning of the growth in 1mM glucose on an anti-adhesive polymer polyHEMA-coated surface [65,66]. Importantly, also in this culturing condition, glucose was completely exhausted at 72h, time at which all the following experiments were performed. As shown in panels I and J of S15 Fig, H89 treatment reduced cell survival in strict association with its inhibitory activity on PKA. In addition, BPTES and CQ treatments increased cell death as previous observed in Transformed and MDA-MB-231 cells (panels K and L in S15 Fig). These results designate the PKA pathway role as a common feature of different cancer cell models to survive in chronic glucose deprivation and to avoid *anoikis* cell death.

### PKA increases *anoikis*-resistance by activating Src and by modulating mitochondrial metabolism

**Fig 9:**
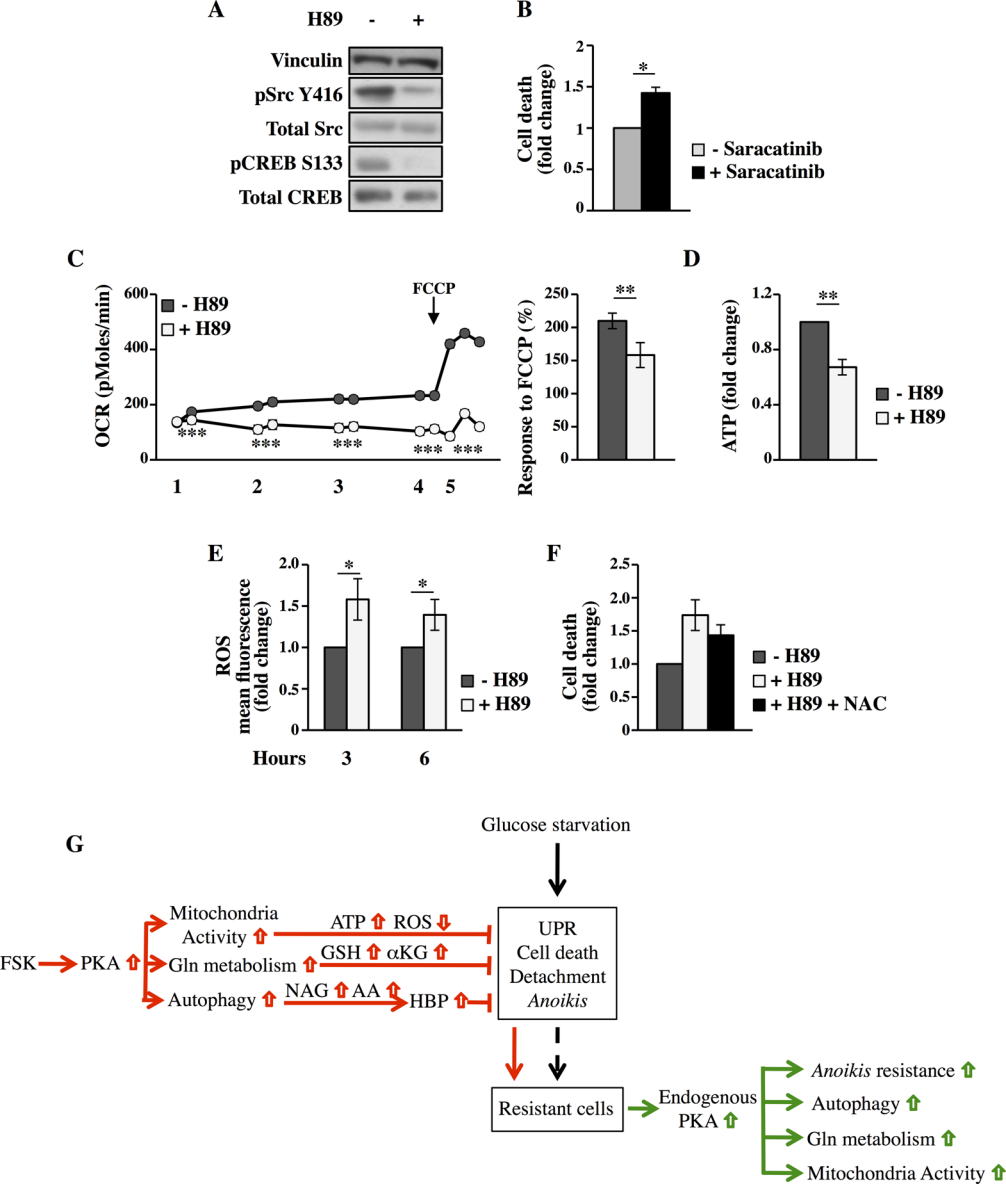
The inhibition of PKA impacts on c-Src phosphorylation, mitochondrial respiration and intracellular ROS levels in MDA-MB-231 suspended cells. All analyses were performed in floating cells collected at 72h of culture in LG. **(A)** After 9h of treatment with DMSO or 10μM H89, Src and CREB phosphorylation was analyzed by Western blot. **(B)** After treatment with 1μM Saracatinib for 9h, trypan blue exclusion assay was performed. **(C)** OCR was measured with the Seahorse instrument. After cell treatment with DMSO or 10μM H89, the OCR of -/+H89 cells was measured every 30 minutes (steps 1–4, two measurements at each step). OCR was then measured after addition of 1μM FCCP (step 5, the last three measurements) to evaluate the mitochondrial reserve capacity (right histogram). The total duration of the analysis was 3–3.5h. The data are represented as mean±SEM. **(D)** Intracellular ATP level was measured in floating cells treated with DMSO or H89 for 3h. **(E)** Intracellular ROS were measured by using H_2_DCFDA in floating cells treated with DMSO or H89 for 3–6h. **(F)** Viable count by trypan blue was performed in floating cells treated with 10μM H89 and 5mM NAC for 6h. All data represent the average of at least three independent experiments. *p<0.05, **p<0.01, ***p<0.001 Student’s t-test. **(G)** Schematic representation of the PKA-mediated resistance to glucose deprivation and *anoikis* in cancer cells.

The loss of attachment to the ECM can cause diverse cellular and molecular changes that eventually contribute to the appearance of *anoikis*-resistant cells [60,67]. Activation or overexpression of growth factor receptors as well as activation of other signaling molecules has been shown to interfere with *anoikis*. Src activity is correlated with *anoikis*-resistance [68,69]. PKA can phosphorylate Src on serine-17 (S17) to regulate its activity and enhance tumor metastasis both in breast and ovarian tumors [70,71]. Since phosphorylation of Src by PKA in S17 directs its activation through auto-phosphorylation of Tyr416 (Y416), we examined whether this occurs in glucose depleted MDA-MB-231 suspended cells. MDA-MB-231 cells displayed consistently elevated Src phosphorylation in Y416 (Fig 9A). Additionally, Src activation was strongly inhibited by PKA inhibitor H89, since upon 9h of treatment the pSrc Y416 was hardly detectable as well as the control pCREB S133. Inhibitors targeting Src kinase have been shown to induce *anoikis*-resistance reversal. Therefore, we used the Src specific inhibitor Saracatinib (AZD0530) [72,73], to test whether we could alter such resistance. As shown in Fig 9B, treatment with the Src-kinase inhibitor induced an increased cell death, similarly to what observed with H89. These data suggest that PKA activation is required for Src activity leading to *anoikis*-resistance.

Recently it has been highlighted that in cancer cells the modulation of metabolic pathways contributes to achieve the *anoikis*-resistant phenotype, for instance by mitigating ROS accumulation and maintaining ATP content upon detachment or by activating autophagy [60,74]. Previously, we have shown that PKA-positive *anoikis*-resistant cancer cells are dependent on glutamine catabolism for their survival. Therefore we hypothesized that glutamine was increasing TCA cycle flux and oxidative phosphorylation in detached cells in order to prevent the ATP drop due to glucose depletion. Oxygen consumption rate (OCR) analysis, performed by using Seahorse instrument, was performed in MDA-MB-231 suspended cells treated or not with PKA inhibitor H89. Significantly higher OCR was observed in untreated cells as compared to treated ones, in all the time points analyzed along the duration of the experiment. To further examine mitochondrial function, we examined also the respiratory reserve capacity by treating the cells with the uncoupler agent carbonyl cyanide 4-(trifluoromethoxy)phenylhydrazone (FCCP) (Fig 9C). We found that the untreated cancer cells had significantly higher respiratory reserve capacity than the H89-treated cancer cells. Interestingly, H89-treatment reduced also the total level of intracellular ATP (Fig 9D), suggesting that PKA activation prevents, by stimulating OXPHOS activity, ATP drop and hence favoring cell survival. Since cell detachment has been shown to induce an increase of intracellular ROS levels [74,75], we sought to measure intracellular ROS levels in MDA-MB-231 suspended cells treated or not with PKA inhibitor H89. The treatment induced a significant increase of ROS as compared to untreated cells (Fig 9E). Notable, treatment of MDA-MB-231 suspended cells with the antioxidants N-acetyl-L-cysteine (NAC) decreased cell death (Fig 9F), underlining a role of ROS in *anoikis*. Collectively these data suggest that PKA activity increasing mitochondrial activity may lead to higher intracellular ATP levels and reduction of ROS, which together to autophagy and Src activation concur to *anoikis*-resistance.

## Discussion

Limited glucose availability is one of the distinguishing features of the tumor microenvironment. It is caused by the poor blood supply and by the high consumption rate of tumor cells [22,76]. However, such energy-depleting condition can often hamper cells by reducing cellular energy and other metabolites below the minimal level required to run bioenergetics and anabolic demand. Therefore, cancer cells acquire adaptive responses, including activation of signaling pathways and metabolic rewiring, to survive in such limiting condition that ultimately may lead to resistant cell population selection.

Here, we show that PKA pathway is a critical signaling involved in cellular response to glucose starvation since it has the capability to activate different cellular processes that together concur in the acquisition by the cancer cells of a resistant phenotype, characteristic of more aggressive cells.

As summarized in Fig 9G, our previous and current results indicate that glucose deprivation induces different responses in cancer cells among which UPR and cell detachment (Fig 9G, black arrows and [12]). However, both processes may lead to cell death, as we have previously shown and further demonstrated throughout this paper, as well as to selection of resistant cells often acquiring different features as compared to non-resistant ones [77,78].

In order to define the mechanisms involved in such cell resistance, in this study we decided to further evaluate the protective role of the PKA pathway upon glucose starvation. In fact, our previous findings indicated that exogenous activation of PKA in glucose starved cancer cells is able to sustain cell survival by increasing mitochondrial activity, intracellular ATP levels and mitochondrial interconnection as well as by decreasing intracellular ROS ([21] and Fig 9G, red color). Otherwise a direct role of PKA in the control of mitochondrial function, glucose homeostasis, insulin secretion and inhibition of cell death has been also shown in hepatocytes and beta cells through its ability to phosphorylate in serine 155 the proapoptotic protein Bcl-2-associated death promoter (BAD). In fact such a phosphorylation is required and sufficient to promote the metabolic function of BAD protein [79,80]. These effects mediated by PKA could be particularly important for survival in absence of glucose; for instance, the cell lines most sensitive to low glucose are unable to up-regulate the oxidative phosphorylation or to make mitochondrial interconnections [6,81]. On the other hand, cells experiencing UPR, as a result of a reduced flux through HBP upon glucose withdrawal [12,32,82], need to improve ER homeostasis by increasing for example the ATP necessary for heat shock protein (HSP)-dependent protein folding and by maintaining the intracellular redox homeostasis [83,84].

In keeping with the idea that PKA may control cancer cell survival in glucose starvation, in this study, we have identified UPR, autophagy and glutamine metabolism as processes directly involved in PKA-dependent cell resistance, starting from data recovered using different “omics” approaches (Figs 1 and 2).

Our data demonstrate that PKA modulates glutamine metabolism through coordinated transcriptional regulation of different genes involved in glutamine related metabolic pathways such as *GLS1*, *IDH1* and *GSS* (Figs 3, 5, S8, S11 and S15G). Such a coordinated regulation has been already demonstrated for oncogenes like Myc [85] and K-Ras [86]. For instance, Myc transcription contributes, by up-regulation of some genes indicated beyond, to the high level of cancer cells glutaminolysis as well as addiction to glutamine [85]. Noteworthy our experiments performed by using [U^13^C_6_]glucose and [U^13^C_5_]glutamine showed that in FSK-treated cells under glucose starvation, glutamine contributes in significant manner to TCA cycle fluxes (Figs 5D, 7B, 7C and 9G), substantiating the hypothesis that glutamine is essential for a cell survival especially in FSK-treated cancer cells. Accordingly, inhibition of glutaminolysis by BPTES is more detrimental to FSK-treated cancer cells as well as to *anoikis*-resistant cells than their untreated counterpart (Figs 5C, 7D and S15H).

According to our study, FSK-treated cells did not experience cell detachment, a known effect of glucose depletion. In this regard our transcriptional, proteomic, and functional results, showing a significant pathway enrichment for catabolic processes like autophagy as well as the transcriptional activation of *NAGK*, the kinase that converts GlcNAc recycled from lysosomal degradation of oligosaccharides to GlcNAc-6P [87], suggest that the increased level of GlcNAc-6P observed in NMR-based metabolomic analysis in FSK-treated sample (Fig 2F), leading to increased *N*-glycosylation of membrane glycoproteins (Figs 3F and 6C), could directly participate in preserving cell attachment and ER homeostasis. Noteworthy PKA may directly control the activity of the enzyme controlling the HBP limiting step, that is glutamine:fructose-6-phosphate amidotransferase (GFAT) by direct phosphorylation [88,89]. Importantly, we show that such FSK-dependent UPR attenuation, both at mRNA and protein levels (Figs 1F, 1J, 2A, 3A, 3B, 3C, 3D, 6A, 6B and S15C), strongly depends on autophagy, since its inhibition by CQ treatment, reactivates UPR leading to increased cell death (Figs 4C, 6G and S15F). Therefore, our studies, to our knowledge, reveal for the first time a novel molecular mechanism pointing to a PKA-dependent regulation of UPR through an up-regulation of catabolic processes like autophagy. These findings are in agreement with our previous observations indicating that starvation-dependent cell detachment and death in mouse and human cancer cells could be prevented by medium addition of GlcNAc [12]. Notable, the PKA and UPR relationship has been recently reinforced by a paper published while our work was under submission, showing that upon ER stress both CREB and UPR pathways are activated, highlighting a possible role of CREB in cell fate under extreme cell stress [90].

In the light of our findings, therefore, we propose that glutamine metabolism and autophagy, enhanced by exogenous PKA activation through a transcriptional program, and putatively by post-translational mechanisms not addressed in this report, contribute to the tolerance to nutrient deprivation leading to a selection of resistant cells characterized by a metabolic rewiring, favoring mitochondrial respiration. This latter result is completely in agreement with our previous findings in which we showed that survival of Transformed mouse fibroblasts and MDA-MB-231 cancer cells exposed to mitochondrial complex I inhibitors, under glucose starvation and FSK stimulation, is dramatically affected [91].

In line with these new findings regarding PKA activation and resistance to glucose starvation, we speculate that tolerance to cell death induced by glucose starvation is normally mediated also by PKA in cancer cells, since different authors have shown that PKA may operate and be deregulated in cancer [92,93]. Importantly, we would underline that this role of PKA has been demonstrated throughout the paper applying several options to inhibit PKA activity namely H89, PKI amide as well as siRNA against PKAc either in FSK-treated or FSK-untreated suspended resistant cells, both characterized by high level of PKA activation. Accordingly, as shown in Fig 9G -green arrows-, the subset of resistant cancer cells displays a strong activation of endogenous PKA pathway depending on glucose starvation and matrix detachment (Fig 8A and 8C and panel B in S13 Fig). Notably, such activation is associated with cell survival and specifically with *anoikis-*resistance (Fig 8B and panel A in S13 Fig). In fact, we show that PKA inhibition in these resistant cancer cells induces cell death, demonstrating that PKA is necessary for survival during such stress conditions, namely glucose starvation and cell detachment (Figs 8F, 8G, 8H, S12B, S12C, S12E, S13C and S13D). The observed endogenous activation of PKA in cancer cells under glucose starvation differs from our previous results [21], in which endogenously PKA was not found so activated upon growth under limiting amount of glucose. However, the experimental procedures are different; here observations were made by using all cell culture population, that means adherent and suspended cells, whereas in Palorini et al. [21], PKA activity analysis was performed only by using adherent cells, in which PKA activation appear clearly lower as compared to suspended cells and less modulated (Fig 8A and 8C, panel B in S13 Fig). Regarding the role of PKA activation in resistant cancer cell aggressiveness, several reports have indicated a role of PKA in actin-based cell migration and invasion of cancer cells [94,95,96]. Moreover more recently it has been shown that PKA mediates resistance to cancer therapy in breast cancer cells [97,98] and that high level of CREB1 expression correlates with metastasis and tumor stage in gastric cancer as well as malignant glioma growth [99,100]. In addition, it has been shown that hyperactivation of cAMP/PKA pathway by genetic manipulation or by stress hormones, like epinephrine, favor breast and ovary tumors respectively [70,71]. Notable in both studies, PKA tumorigenic activity is associated to Src activation, as demonstrated by phosphorylation of its catalytic tyrosine (Y416). Accordingly, our *anoikis-*resistant breast cancer cells show a PKA-dependent Y416 phosphorylation, since it appears strongly sensitive to PKA inhibition by H89 (Fig 9A). Otherwise the important role of Src in such *anoikis*-resistant cancer cells it has been further confirmed by the observed increase in cell death upon Saracatinib treatment (Fig 9B). Such findings, given the important role of PKA pathway in mediating effects of hormones and nutrients on energy homeostasis by controlling cell metabolism [101], may shed new light on the relation between *anoikis*-resistance, metastasis and metabolic rewiring of cancer cells.

Importantly, the resistant cells identified in our experiments showed a strengthening of the same metabolic processes observed in FSK-treated cancer cells, namely autophagy, glutamine metabolism and mitochondrial activity, supporting a common mechanism of action upon exogenous and endogenous stimulation. In fact, the treatment with BPTES and CQ clearly affected suspended cell survival (Figs 8I, 8J, S13F, S13G, S15K and S15L). As a result, this was not unexpected since it has been shown that matrix detachment has a profound effect on cellular metabolism [60]. Accordingly, the *anoikis*-resistant cells show a mitochondrial activity strongly sensitive to PKA inhibition, given that both basal and uncoupled respiration were reduced by H89 treatment (Fig 9C). Interestingly, upon inhibition we observed an increase of intracellular levels of ROS. As previously illustrated, ROS are considered critical players in *anoikis*-resistance due to their positive effect on pro-survival cell signaling and tumor metastasis [60]. However excessive and unmitigated ROS increase can result in cell death [102,103,104]. Remarkably, our results indicated that the antioxidant NAC can partially rescue H89-treated detached cells, underscoring the fact the PKA controls an antioxidant response critical for *anoikis*-resistance. Otherwise it has been shown that antioxidants can prevent *anoikis* maintaining high ATP levels and metabolic activity as consequence of an enhanced fatty acid oxidation [75,105], further supporting an important role of mitochondria activity in *anoikis*-resistance as we observed in our suspended cells (Fig 9G).

While cAMP-PKA axis fulfills all basic requirements as a candidate for the intracellular signal that modulates cancer cell survival under glucose starvation, as shown by our experiments using the PKA and Epac inhibitors (panels B, C and D in S10 Fig and panel F in S12 Fig), we still cannot completely exclude that also Epac branch mediates some cellular response. In fact, in order to test this branch we have used the Epac inhibitor ESI-09 [106]. This inhibitor has been shown to work within the 1–10μM range [57], since at higher concentration cell viability may be affected by appearance of cytotoxicity. Accordingly also in our experiments we observed cell death appearance upon few hours of 10μM ESI-09 treatment (panel E in S10 Fig). Therefore our experiments have been performed by using a non-cytotoxic concentration (1μM daily treatment), which is already shown to be able to inhibit the agonist 007-AM-mediated Epac activation [56]. However, this branch warrants further investigation, for instance by a genetic approach, since it has been implicated in various important cellular processes [55].

Importantly we would underline that our data, as compared to other authors testing the effect of glucose starvation on cancer cells, have been obtained for both cancer cells without subsequent media changes, which led to glucose exhaustion over time, and hence giving the cells the opportunity to adapt their metabolism. In fact, in tumors the glucose normally drops slowly, depending on tumor growth and vascularization, from fasting blood glucose levels of 4–6mM to 0.6–0.4mM into the tumors. We believe this is a tempting starting point to study cancer cells under glucose depletion.

In conclusion, the results showed in this report have important implications regarding metabolic cancer therapeutics aimed at restraining the supply or utilization of glucose. In fact such treatments could trigger reprogramming of the metabolic homeostasis of cancer cells, allowing for the efficient utilization of alternative nutrients and metabolic pathways for growth, thus increasing tumor aggressiveness. In this regard, our data demonstrate that PKA is a determinant of metabolic plasticity and suggest that attacking the glycolysis-addiction of tumors would actually be effective in cancer therapy as long as PKA is also inhibited. Further studies are needed to better understand the details of cAMP-PKA axis in resistant cancer cells, but from our findings PKA emerges as a critical pro-survival factor that can be exploited by tumors to support adaptation to metabolic stress.

## Materials and Methods

### Cell culture and treatments

Mouse embryonic fibroblast NIH3T3, MDA-MB-231 and MIA PaCa-2 cells (obtained from ATCC, Manassas, VA, USA) and K-Ras-transformed NIH3T3-derived cell line 226.4.1 [107] were cultured in DMEM containing 4 mM L-glutamine, 100 U/ml penicillin and 100 mg/ml streptomycin and pyruvate free (complete medium), supplemented with 10% newborn calf serum (mouse cells) or 5–10% fetal bovine serum (human cells). All reagents were purchased from Life Technologies (Carlsbad, CA, USA).

For analysis, cells were plated at a density of 3000 cells/cm^2^ (mouse fibroblasts and MDA-MB-231) or 5000 cells/cm^2^ (MIA PaCa-2) in complete medium. After 16h cells were washed with phosphate buffer saline (PBS) and incubated in growth medium (time 0) glucose and pyruvate free, supplemented with 25 mM (HG) or 1 mM (LG) glucose (Sigma-Aldrich Inc., St. Louis, MO, USA). Cells were then collected for analysis as described in figure legends.

For specific treatments, cells were daily treated with 10μM FSK from Coleus Forskohlii (Sigma-Aldrich), starting 24h after medium replacement and then analyzed at time points indicated in figure legends. As indicated, ESI-09 (Biolog Life Science Institute, Bremen, Germany), H89 (AdipoGen Life Sciences, San Diego CA, USA), PKI amide (Enzo Life Sciences, Farmingdale, NY, USA), CQ and BPTES (both from Sigma-Aldrich) were used alone or in combination with FSK (see S1 and S12 Figs for treatments). Saracatinib (AZD0530) was purchased from Selleckchem (Boston, MA, USA), NAC from Sigma-Aldrich.

The analyses with MIA PaCa-2 in suspension were performed seeding cells onto multi-well plates coated with poly-HEMA (poly-2-hydroxyethyl methacrylate, from Sigma-Aldrich). To coat 12-well culture plates, 0.5 mL of a 20mg/ml solution of poly-HEMA in 95% ethanol was applied twice to each well, dried, and extensively washed with PBS. To avoid potential anti-apoptotic effects caused by clumping, cells were seeded also in presence of 0.8% methylcellulose (Sigma-Aldrich).

### Gene Chip analysis

Gene expression levels were processed, normalized and summarized using the Robust Multi-array Average (RMA) method [108] as implemented by the R/Bioconductor package “oligo”. Probeset/gene annotations were retrieved from ENSEMBL. Probes not mapping to an Entrez gene were discarded. The resulting dataset was composed of 22035 probes mapping to 17669 genes. NCBI GEO database accession number GSE68266. The full procedure for Gene Chip analysis, Differential expression analysis and Gene set enrichment analysis may be found at https://github.com/yp/gpa-scripts.

### Proteomic analysis

For protein extraction, 2D-DIGE and for protein identification by matrix-assisted laser desorption/ionization time-of-flight (ToF)/ToF mass spectrometry were performed as previously described [12]. Refer also to S1 Text for further details.

### NMR-based metabolic analysis

The water-methanol extraction was adapted from the protocol for tissues and human cell cultures [109] In particular, 500 μL of methanol (4°C) were added to the frozen cell pellets (1.5x10^7^ cells) on ice and were resuspended after 5 minutes. The sample was frozen in liquid nitrogen for 15 minutes, sonicated for 20 minutes, vortexed until a homogeneous emulsion was obtained and then centrifuged at 14000xg for 5 minutes. The supernatant was collected while the pellet was re-suspended in 500 μL of methanol (4°C) and the steps previously described repeated. The pellet was finally suspended in 300 μL of MilliQ water (4°C) and then frozen in liquid nitrogen for 15 minutes, sonicated for 20 minutes, vortexed until a homogeneous emulsion was obtained and then centrifuged at 14000xg for 5 minutes. The three supernatants were put together and water and methanol were evaporated under argon pressure. Dry extracts were stored at -80°C until the NMR measurements were taken. NMR spectroscopy and metabolite extraction was performed as described in S1 Text.

### Differential expression analysis

Differential expression analysis of the gene and proteomic datasets was separately performed on the two datasets using the linear modeling approach implemented by the R/Bioconductor package “limma” [110,111]. Differential expression results of probes/protein spots mapping to the same gene were filtered according to their minimum p-value.

### Gene set enrichment analysis (GSEA)

Gene set enrichment was performed using as gene sets the 202 KEGG pathways classified as metabolic or signaling. The pathways contain 6885 unique genes of which 6010 were included in our gene *All expressed mRNA list* dataset and 61 in the protein dataset (S4 Table). Nine different gene set enrichments were performed on the two dataset—gene and protein—according to nine different gene set statistics (Namely, Mean, Median, Sum, Maxmean, GSEA PAGE, Reporter features, Wilcoxon rank-sum test, and Tail strength) as implemented by the R/Bioconductor package “Piano” [24]. The same package was used for the integrations of the 9 enrichments within each dataset.

### Protein-protein interaction analysis

Protein-protein associations were computed using the web-based platform of STRING v9.1 [23]. In particular protein–protein interactions, based on automated text mining for co-occurrence were performed by using a medium confidence (0.400) for both differentially expressed genes and proteins (DEGs and DEPs). The same platform was used for KEGG pathway enrichment. The terms were sorted by their enrichment P-values. For DEGs were considered significant only the pathway with a FDR≤0.05. For DEPs were considered significant only the pathway with a P-value≤0.05 without any correction testing.

### Gene expression analysis by qPCR

RNA was extracted from cells using TRIZOL reagent (Life Technologies). 1 μg of total RNA was reverse-transcribed with oligo-dT using QuantiTect Reverse Transcription Kit (QIAGEN, Valencia, CA, USA) according to the manufacturer’s protocol. 0.2 μg of the product of reverse transcription was amplified by qPCR with an Applied Biosystem 7500 standard system (Thermo Fisher Scientific) (Waltham, MA, *USA*) using POWER SYBR GREEN PCR mix for qPCR (Life Technologies). Primers were designed using Primer3Plus software (http://www.bioinformatics.nl/cgi-bin/primer3plus/primer3plus.cgi) and used at 0.25μM. The relative level of expression was calculated by the 2^−ΔΔCT^ method β-actin and rRNA18S were used as endogenous control. The list of primers is shown in S2 Text.

### Western blot analysis

Cells were harvested and disrupted in a buffer containing 50mM HEPES pH 7.5, 150mM NaCl, 1% (v/v) glycerol; 1% (w/v) Triton X-100, 1.5mM MgCl2, 5mM EGTA, protease inhibitor cocktail (Sigma-Aldrich) and phosphatase inhibitors (Sigma-Aldrich). 10 to 30μg of total protein were resolved by SDS-PAGE and transferred to the nitrocellulose membrane, which was incubated overnight with specific antibodies: vinculin, Grp78, CHOP (GADD153) IDH1 and GSS from Santa Cruz Biotechnology Inc. (Santa Cruz, CA, USA); phospho-(Ser/Thr) PKA Substrate, phospho-CREB S133, total CREB, Beclin-1, LC3, Eif2α, cleaved caspase 3, phospho-Src (Y416) and total Src (36D10) from Cell Signaling Technology Inc. (Danvers, MA, USA); O-Linked N-Acetylglucosamine (Clone RL2) from Thermo Scientific (Waltham, MA, *USA*).

### PKA activity

To determine the PKA activity an ELISA assay was performed through the PKA kinase activity kit from Enzo Life Sciences. The analysis was performed following manufacturer’s datasheet using crude samples. For each determination 15ng (mouse fibroblasts) or 50ng (MDA-MB-231) of whole culture cell extract were used. It should be noted that in -/+FSK samples both the ELISA assay of PKA activity and the Western blot analysis of phospho-(Ser/Thr) PKA Substrate and pCREB S133 were always performed 1–2h after the last FSK treatment, because of the transient activation of PKA. At this time of treatment the highest level of activity and PKA-substrates phophorylation can be observed (see also [21]).

### Silencing of PKAc

The siRNA duplex for human PKAcat α (siPKAc) was purchased from Sigma-Aldrich with the sequence as follows: 5’-CCUGCAAGCUGUCAACUUU-3’. The negative control siRNA (siCTRL) was purchased from QIAGEN. The transfection of siRNA oligonucleotides was performed with INTEFERin reagent (Polyplus transfection, New York, NY, USA), as described in [12] with little modifications depending on the well format. The final siRNA concentration was 30nM.

### Flow cytometry analysis of N-linked glycoproteins

To determinate cell surface expression of N-linked glycoproteins, cells were stained with Concanavalin A, Alexa Fluor 594 conjugate lectin (absorption/emission maxima ~590/617 nm) and analyzed by FACSCalibur flow cytometer (Becton-Dickinson, Franklin Lakes, NJ, USA) with CellQuestPro software (Becton-Dickinson). Flow cytometric data were collected using Flowing Software.

### MDC incorporation assay

Cells were plated onto clean glass slides lodged in six-well plates under indicated growth conditions. At indicated time points 50μM MDC was loaded into cells in DMEM at 37°C, 10 minutes. After incubation, cells were washed twice with cold PBS. Cells were immediately analyzed by fluorescence microscopy using Nikon eclipse 90i equipped with a filter covering excitation wavelength 380 nm; emission, 525 nm (Nikon, Tokyo, Japan). The images were acquired with NIS Elements D 4.30.00 64-bit software.

### Immunofluorescence

Cells were plated (see above) onto coverslips lodged in six-well plates under indicated growth conditions. They were fixed with 4% paraformaldehyde for 15 minutes and permeabilized with 0.1% Triton X-100 for 10 minutes. Subsequently, cells were first subjected for 1h at room temperature (RT) to a blocking solution of 10% horse serum in PBS, and then were labeled with an LC3 antibody (1:500), for 1h at RT. LC3 detection was obtained using the Alexa 546-labeled anti-rabbit secondary antibody (1:800) (Life Technologies) at RT, upon twice washes with cold PBS. Coverslips were washed in PBS, stained with DAPI (0.5ng/ml) for 2 minutes at RT and mounted with DABCO (Sigma-Aldrich). For the analyses of PKA and caspase activation on floating cells, cells were collected, stained with fluorescent substrate of the active caspase 3 (NucView Caspase 3 Substrate, Biotium) as indicated by manufacturer’s protocol and then attached to the coverslip using cytospin. Then they were fixed and labeled with the phospho-(Ser/Thr) PKA Substrate antibody (1:200). Cells were visualized by fluorescence microscopy (see above).

### Colony formation assay

Following culture in LG, floating cells were collected and treated for 6h with DMSO or 10μM H89 and then re-plated at low density in HG. After 14 days, cells were washed twice with PBS, fixed in PBS-formaldehyde 5%, and stained with 0.1% crystal violet for 5 minutes. After colorant dissolving by acetic acid 10% the absorbance was analyzed at spectrophotometer.

### Glutamine uptake and glutamate and ammonia secretion

After collection of the culture media before and subsequent to cell cultivation glutamine and glutamate levels were measured with a 2900D YSI biochemistry analyzer (YSI, Yellow Springs, OH, USA). Ammonia was analyzed using a colorimetric assay kits from BioVision (Milpitas, CA, USA) according to the manufacturer’s protocol.

### Stable-isotope labeling experiments

For the labeling experiments the cells were seeded as previously described. After 16h, during the medium change, cells were incubated with unlabeled glucose or glutamine and either 1mM [U^13^C_6_]glucose or 2mM [U^13^C_5_]glutamine (both tracers from Cambridge Isotope Laboratories, Tewksbury, MA, USA). At specified time, cells were quickly rinsed with 0.9% NaCl and quenched with ice-cold methanol. A liquid-liquid extraction method was employed to extract intracellular metabolites. Dried polar metabolites were submitted to a two-step derivatization and subsequent gas chromatography/mass spectrometry (GC/MS) analysis. MIDs of target metabolites were corrected for natural occurring isotopes with the MetaboliteDetector software [112,113]. The extended protocol is presented in S1 Text.

### Measurement of OCR with the Seahorse instrument

Oxygen consumption was determined using the Seahorse XF24 extracellular flux analyzer (Seahorse Bioscience, North Billerica, MA, USA). Briefly, after culture in LG, floating cells were collected and seeded onto wells of a dedicated 24-well XF24 cell culture plate previously coated with Corning Cell-Tak adhesive (Corning, New York, NY, USA), in unbuffered medium without glucose (complete formulation: XF unbuffered DMEM supplemented with 1% dialyzed FBS, 2mM Sodium Pyruvate and 2mM glutamine). After seeding, the cells were incubated for 40 minutes at 37°C without CO_2_ before to run the experiment. H89 and FCCP were injected by the instrument.

### Analysis of intracellular ROS

After culture in LG, floating cells were collected, treated with 10μM H_2_-DCFDA and incubated for 30 minutes at 37°C. After that, without any wash, the cells were also treated with DMSO or H89 for the desired time. The fluorescence was then analyzed by using both FACScan flow cytometer (Becton‐Dickinson, Franklin Lakes, NJ, USA) and Varian Cary Eclipse fluorescence spectrophotometer (Agilent Technologies, Santa Clara, SA, USA). Because phenol red interferes with H_2_-DCFDA, the cells were seeded, collected and analyzed in medium without phenol red.

### Analysis of intracellular ATP

Intracellular ATP levels were measured using a luciferin‐luciferase assay (Promega, Madison, WI, USA) [21].

### Statistics

Data are expressed as means ± standard deviations and represent one of at least three separate experiments undertaken in triplicate, unless stated otherwise. Differences between data sets were determined using Student’s *t-test*. Differences described as significant in the text correspond to P values of *P<0.05; **P<0.01; ***P<0.001.

## Supporting Information

S1 Fig Graphical representation of the experimental workflow -/+FSK. **A, D** For the analyses upon treatment with FSK Normal and Transformed cells (A) as well as MDA-MB-231 (D) were cultured in LG medium (1mM as initial concentration) 16h after seeding. Then, starting from 24h the cells were daily treated with DMSO (vehicle) or 10μM FSK. When required, at 72h (for Transformed cells, A) or 48h (for MDA-MB-231, D), time at which the glucose in the medium is completely exhausted, cells were treated with the specific drugs indicated throughout the manuscript. To investigate their behavior the cells were harvested and analyzed at specific time points, as indicated in the main text, after the last treatment. See each figure for specific details. **B, E** PKA activity after FSK treatment was evaluated by Western blot analysis of p-(Ser/Thr) PKA substrates and pCREB S133 as well as by an ELISA assay in total cellular extracts of Transformed (B) and MDA-MB-231 (E). **C, F** Representative pictures of Transformed (C) and MDA-MB-231 (F) cell population -/+FSK after 96 and 72h of cultivation in LG, respectively.
(PDF)

S2 Fig The genes regulated by FSK in Normal cells show a high degree of connection. In the figure the network of predicted associations for all DEGs-encoded proteins in NF/N comparison is shown. The STRING analysis of the protein-protein interactions was performed to DEGs with fold change ≥2 in the comparison.
(PDF)

S3 Fig The genes regulated by FSK in Transformed cells show a low degree of connection. In the figure the network of predicted associations for all DEGs-encoded proteins in TF/T comparison is shown. The STRING analysis of the protein-protein interactions was performed to DEGs with fold change ≥2 in the comparison.
(PDF)

S4 Fig Network of predicted associations for all the differentially expressed proteins identified by 2-DIGE. The STRING analysis of the protein-protein interactions was performed using proteins with spot variation ≥10% in NF/N (A) and TF/T (B) comparisons.
(PDF)

S5 Fig Analysis of transcriptomic and proteomic data using PIANO method. The heatmap shows the result obtained by applying the PIANO tool to gene (A) and protein (B) datasets separately. In particular, the top 10-ranked pathways associated to each comparison, NF/N and TF/T, are shown. The different color of the heatmap represents the rank position of the pathway in the two different comparisons.
(PDF)

S6 Fig The FSK treatment attenuates UPR in both Normal and Transformed cells. The analysis shown here was performed in cells cultured for 72h in LG and daily treated with DMSO or 10μM FSK. **A-B** mRNA expression of UPR-related genes was analyzed by qPCR for Transformed (A) and Normal (B) cells. mRNA expression levels in FSK-treated cells are reported as change (n-fold) with respect to the amount of relative mRNA expressed in untreated cells, using β-actin mRNA as internal control. **C** Agarose gel electrophoresis was performed to detect the unspliced and spliced forms of Xbp1. All data represent the average of three independent experiments. The error bar indicates the standard deviation while the asterisks indicate statistical significance determined by Student’s t-test (*p<0.05, **p<0.01, ***p<0.001; n.s. not significant).
(PDF)

S7 Fig The induction of the PKA pathway mediates the autophagy activation in Transformed cells in glucose deprivation. **A** PKA activation was evaluated by Western blot analysis of p-(Ser/Thr) PKA substrates and pCREB S133 in Transformed cells daily treated with FSK and/or 2μM H89. **B** The cellular morphology of the cells -/+ FSK and -/+H89 was observed at 96h of culture and representative microscopy images are shown. **C-E** Different analyses were performed to evaluate the autophagy in Transformed cells -/+FSK and -/+H89. **C** Western blot analysis of Beclin1 expression level in cells -/+FSK. **D-E** Evaluation of LC3B-I conversion in LC3B-II by Western blot (D) and staining with 50μM MDC (E). Precisely, in these last analyses (72h of culture) the cells were treated with FSK 1h before the addition of 10μM H89 to -/+FSK samples and then were collected after additional 9h. The cells with MDC were analyzed using fluorescence microscopy at 60X magnification. Scale bar 10μm. All data are representative images of three independent experiments.
(PDF)

S8 Fig The treatment with FSK induces a relevant change in the expression of genes related to the glutamine metabolism. Transcriptional data from microarray analysis regarding glutamine metabolism-related genes in Transformed cells at 72h of culture in LG, daily treated with DMSO or FSK. Data express the ratio in TF/T comparison.
(PDF)

S9 Fig The inhibition of PKA counteracts the protective effects of FSK in MDA-MB-231. MDA-MB-231 cells were analyzed upon daily treatment with FSK and 2μM H89. **A** PKA activation by Western blot analysis of p-(Ser/Thr) PKA substrates and pCREB S133. **B** Microscopy images of the cells were collected at 72h of culture. **C** Western blot analysis of Grp78 and CHOP was performed at 48h. **D-E** To analyze the effects of PKA inhibitor H89 on FSK-dependent induced autophagy, Western blot analysis of LC3B-I conversion in LC3B-II (D) and the staining with 50μM MDC were performed. Precisely, in these last analyses (48h of culture) the cells were treated with FSK 1h before the addition of 10μM H89 to -/+FSK samples and then were collected after additional 9h. The cells with MDC were analyzed using fluorescence microscopy at 60X magnification. Scale bar 10μm. **F-H** Cells were transfected with siRNA (control–siCTRL- or for PKAcat α –siPKAc-) after the medium change (directly in LG medium) and then daily treated with DMSO or FSK for 48h. **F** The expression of PKAcat α was detected by Western blot. PKA activity was evaluated by ELISA assay in total cellular extracts. **G** Microscopy pictures were collected and trypan blue exclusion assay was performed. **H** Western blot analysis of LC3B-I conversion in LC3B-II was performed. All data represent the average of at least three independent experiments. *p<0.05, **p<0.01, ***p<0.001 Student’s t-test.
(PDF)

S10 Fig FSK-protective role is not mediated by Epac. **A** Schematic representation of FSK-mediated activation of PKA and Epac proteins upon intracellular cAMP enhancement. To analyze the effect of Epac inhibition, the cells were daily treated with FSK and/or 1μM Epac inhibitor (ESI-09). **B** Cells -/+FSK and -/+ESI-09 were counted with trypan blue at 72h of culture. **C** To analyze the effects of Epac inhibitor ESI-09 in the FSK-dependent induced autophagy, MDC staining was performed in viable cells at 72h. **D** Protein levels of Grp78 and CHOP were analyzed by Western blot at 48h of culture. **E** Cells were treated with 10μM ESI-09 for 8h and counted with trypan blue. All data represent the average of at least three independent experiments. *p<0.05, **p<0.01, ***p<0.001 Student’s t-test.
(PDF)

S11 Fig FSK-treated MDA-MB-231 cells show a different expression of glutamine metabolism-related genes as compared to untreated cells. The expression of genes related to the glutamine metabolism was performed in MDA-MB-231 cells daily treated with DMSO or FSK after 48h and 72h of cultivation in LG. mRNA expression of glutamine metabolism-related genes was analyzed by qPCR. mRNA expression levels in FSK-treated cells are reported as fold change with respect to the amount of relative mRNA expressed in untreated cells, using β-actin mRNA as internal control. All data represent the average of at least three independent experiments. Student's t-test (*p<0.05; **p<0.01; ***p<0.001; n.s., not significant).
(PDF)

S12 Fig The inhibition of PKA prevents the resistance to glucose deprivation and *anoikis*. **A** Graphical representation of the experimental workflow for analyses in *anoikis*-resistant floating cells. MDA-MB-231 cells were cultured in LG. At 72h, when a significant part of the cell population was detached from the plate, the suspended cells were collected and treated with different drugs (as indicated in the specific figures) or the correspondent vehicle and analyzed 3-6-9-24h later. The same scheme was followed also for Transformed cells but the suspended cells were collected at 96h. See the specific figure for details. **B-D** MDA-MB-231 floating cells were collected and treated with DMSO, 150μM PKI amide or 10μM H89 for 24h. After treatment, trypan blue exclusion assay (B) and a colony formation assay (C) were performed. In the same samples the PKA activity was evaluated by ELISA assay (D). **E** MDA-MB-231 floating cells previously transfected with siRNA (control–siCTRL- or for PKAcat α –siPKAc-) were separated from the adherent cells and counted with trypan blue after 8h. **F** MDA-MB-231 floating cells were collected and treated with DMSO or 1μM Epac inhibitor (ESI-09) for 6h and 24h. After treatment, trypan blue exclusion assay was performed. All data represent the average of at least three independent experiments. *p<0.05, **p<0.01, ***p<0.001 Student’s t-test.
(PDF)

S13 Fig PKA pathway is endogenously activated in Transformed cells cultured in glucose deprivation, mediating their resistance to *anoikis*. **A** At indicated time points of culture in LG, floating cells were stained with trypan blue and counted to verify whether floating cells were resistant to *anoikis*. Percentage of floating and dead cells was reported over total population (floating plus adherent cells). All the values have p<0.01 (Student’s t-test) over total cells. **B** At 96h of culture in LG adherent and suspended cells were separately collected to evaluate PKA pathway activation by Western blot analysis of p-(Ser/Thr) PKA substrates and pCREB S133. **C-G** All analyses were performed in floating cells collected at 96h of culture in LG. **C** Floating cells were collected and treated with DMSO or 10μM H89 for 3-6-9h. After treatment, trypan blue exclusion assay was performed. **D** A colony formation assay was performed with floating cells treated with DMSO or H89 for 6h. The colony formation was evaluated after 14 days of culture in HG as crystal violet absorbance. **E** PKA activity and LC3B-I/LC3B-II expression were evaluated by Western blot in floating cells treated with H89 for 6h. **F-G** Viable count by trypan blue was performed in floating cells treated with 10μM BPTES (F) or CQ (G) for 6h. All data represent the average of at least three independent experiments. *p<0.05, **p<0.01, ***p<0.001 Student’s t-test.
(PDF)

S14 Fig PKA and autophagy activation in suspended MDA-MB-231 cells. **A** MDA-MB-231 cells treated with FSK were collected and stained with fluorescent substrate of the active caspase-3 and then newly attached to the coverslip using cytospin. Then they were fixed and labeled with the antibody for PKA substrates. The images were visualized by using fluorescent microscopy at 60X magnification. Scale bar 10μm. **B** PKA activation and LC3B-I/LC3B-II expression were evaluated by Western blot on floating cells treated with 10μM H89 for 6h. All data are representative images of three independent experiments.
(PDF)

S15 Fig PKA activation protects MIA PaCa-2 pancreatic cancer cells by glucose deprivation-induced cell death. **A-H** For the analyses of -/+FSK MIA PaCa-2 cells were cultured in LG and daily treated with DMSO or FSK. **A** Microscopy images of cell morphology. **B** PKA activity after FSK treatment was evaluated by Western blot analysis of p-(Ser/Thr) PKA substrates and pCREB S133. **C** Protein levels of Grp78 and CHOP were analyzed by Western blot at 72h of culture. **D** MDC staining was performed in viable cells. **E** Trypan blue exclusion assay was performed in cells -/+ FSK, treated or not with 30μM CQ for the last 24h of culture. Data are plotted as fold change over the equivalent control sample (- CQ). **F** Expression level of Grp78 and CHOP proteins was analyzed by Western blot in cells -/+ FSK and -/+ CQ with densitometric values. **G** qPCR was performed in cells -/+ FSK at 72h of culture. **H** -/+ FSK cells were treated with BPTES for 24h and counted at 96h of culture. Percentage of reduction after the treatment is shown. **I-L** All analyses were performed in cells grown in LG in wells coated with poly-HEMA. All treatments were performed at 96h of culture. **I-J** Cells were treated with DMSO or 10μM H89 for 9h. After treatment, PKA activity was evaluated by Western blot (I) and trypan blue exclusion assay was performed (J). **K-L** Viable count by trypan blue was performed on cells treated with 20μM BPTES (K) or 30μM CQ (L) for 9h. All data represent the average of at least three independent experiments. *p<0.05, **p<0.01, ***p<0.001, Student’s t-test.
(PDF)

S16 Fig Glucose depletion induces low *anoikis*-resistance in MIA PaCa-2 when cultivated on standard support. **A** MIA PaCa-2 cells were cultured in LG (1mM glucose as initial concentration). The amount of glucose in the medium was measured using an enzymatic kit. **B-C** MIA PaCa-2 cells cultured in HG and LG were counted at different time points with Trypan blue, separating adherent and suspended cells. The percentage of suspended cells in total population (B) and of alive suspended cells (C) is shown.
(PDF)

S1 Table Highly differentially expressed genes in the two comparisons NF/N and TF/T. The two spreadshits represent the genes with logFC > = 1 in the two comparisons NF/N and TF/T and shown in the heatmap of Fig 1B
(XLS)

S2 Table Proteins identified by mass spectrometry in the two comparisons NF/N and TF/T. The two spreadsheets show the mass spectrometry data, the UniProtKB accession number and percentage of the volume spot variation.
(XLS)

S3 Table Total gene expression analysis. The table show the 17669 transcripts used for PIANO analysis and represented as hetamap in Fig 1C. N: Normal cells; N+F: Normal cells + FSK; T: Transformed cells; T+F: Transformed cells + FSK.
(XLS)

S4 Table Genes and proteins identified in our study and used for the Gene set enrichment analysis. Transcriptional and proteomic data have been mapped in the 202 KEGG pathways (metabolic and signaling).
(XLS)

S5 Table The table reports the rankings obtained by each KEGG pathway (rows) in the two comparisons (N+F vs N and T+F vs T) in the transcript, in the proteomic, and in the integrated/aggregated dataset for the three directionality classes (down- and up-regulation, and non-directional regulation). Pathways in the first 10 positions for the transcript and integrated dataset or in the first 5 positions for the proteomic dataset are highlighted in orange. Furthermore, for each pathway, the table presents the total number of genes/protein (column "Total") in the input data and the number of genes/proteins that where up- or down-regulated (columns "No. of up", "No. of down").
(XLS)

S6 Table Pathways used for Venn diagram. (XLS)

S7 Table NMR identified metabolite in N, N+F, T and T+F cells. Metabolite identified by NMR analysis for three independent biological replicates and mean and standard deviation thereof.
(XLS)

S1 Text Supplementary methods. (PDF)

S2 Text List of primers used for qPCR amplification of target genes. Table A: Normal and Transformed cells. Table B: MDA- MB-231 cells
(PDF)
