# Supplementary material for: Protein Kinase A Activation Promotes Cancer Cell Resistance to Glucose Starvation and Anoikis
Source: PLoS Genet. 2016 Mar 15;12(3):e1005931. doi: 10.1371/journal.pgen.1005931 (PMC4792400; doi:10.1371/journal.pgen.1005931)
Supplement: S1 Fig — A, D For the analyses upon treatment with FSK Normal and Transformed cells (A) as well as MDA-MB-231 (D) were cultured in LG medium (1mM as initial concentration) 16h after seeding. Then, starting from 24h the cells were daily treated with DMSO (vehicle) or 10μM FSK. When required, at 72h (for Transformed cells, A) or 48h (for MDA-MB-231, D), time at which the glucose in the medium is completely exhausted, cells were treated with the specific drugs indicated throughout the manuscript. To investigate their behavior the cells were harvested and analyzed at specific time points, as indicated in the main text, after the last treatment. See each figure for specific details. B, E PKA activity after FSK treatment was evaluated by Western blot analysis of p-(Ser/Thr) PKA substrates and pCREB S133 as well as by an ELISA assay in total cellular extracts of Transformed (B) and MDA-MB-231 (E). C, F Representative pictures of Transformed (C) and MDA-MB-231 (F) cell population -/+FSK after 96 and 72h of cultivation in LG, respectively. (PDF) [file pgen.1005931.s001.pdf]

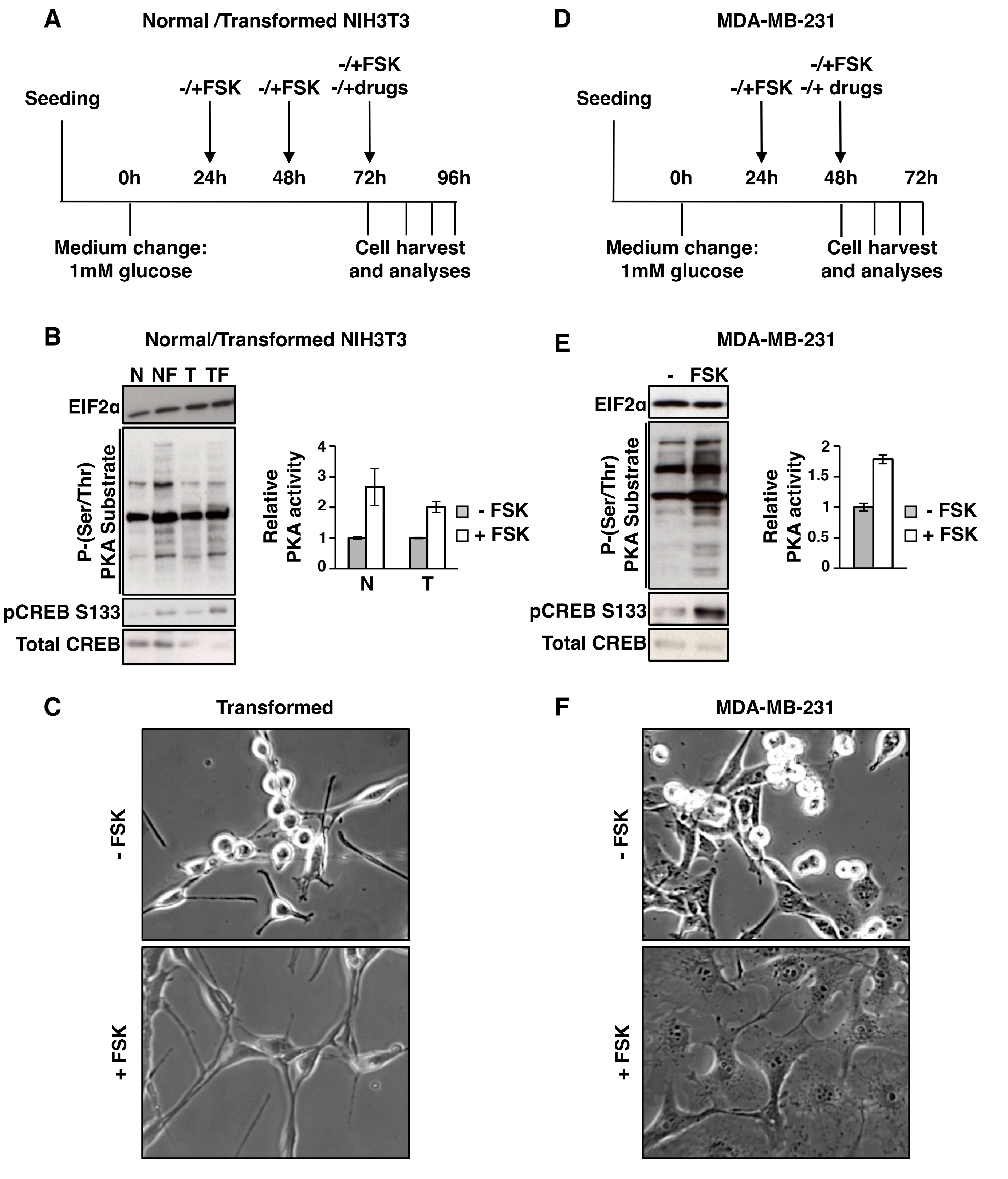

S1 Fig. Graphical representation of the experimental workflow -/+FSK. A, D For the analyses upon treatment with FSK Normal and Transformed cells (A) as well as MDA-MB-231 (D) were cultured in LG medium (1mM as initial concentration) 16h after seeding. Then, starting from 24h the cells were daily treated with DMSO (vehicle) or 10μM FSK. When required, at 72h (for Transformed cells, A) or 48h (for MDA-MB-231, D), time at which the glucose in the medium is completely exhausted, cells were treated with the specific drugs indicated throughout the manuscript. To investigate their behavior the cells were harvested and analyzed at specific time points, as indicated in the main text, after the last treatment. See each figure for specific details. **B, E** PKA activity after FSK treatment was evaluated by Western blot analysis of p-(Ser/Thr) PKA substrates and pCREB S133 as well as by an ELISA assay in total cellular extracts of Transformed (B) and MDA-MB-231 (E). **C, F** Representative pictures of Transformed (C) and MDA-MB-231 (F) cell population -/+FSK after 96 and 72h of cultivation in LG, respectively.
