# Supplementary material for: Protein Kinase A Activation Promotes Cancer Cell Resistance to Glucose Starvation and Anoikis
Source: PLoS Genet. 2016 Mar 15;12(3):e1005931. doi: 10.1371/journal.pgen.1005931 (PMC4792400; doi:10.1371/journal.pgen.1005931)
Supplement: S2 Fig — In the figure the network of predicted associations for all DEGs-encoded proteins in NF/N comparison is shown. The STRING analysis of the protein-protein interactions was performed to DEGs with fold change ≥2 in the comparison. (PDF) [file pgen.1005931.s002.pdf]

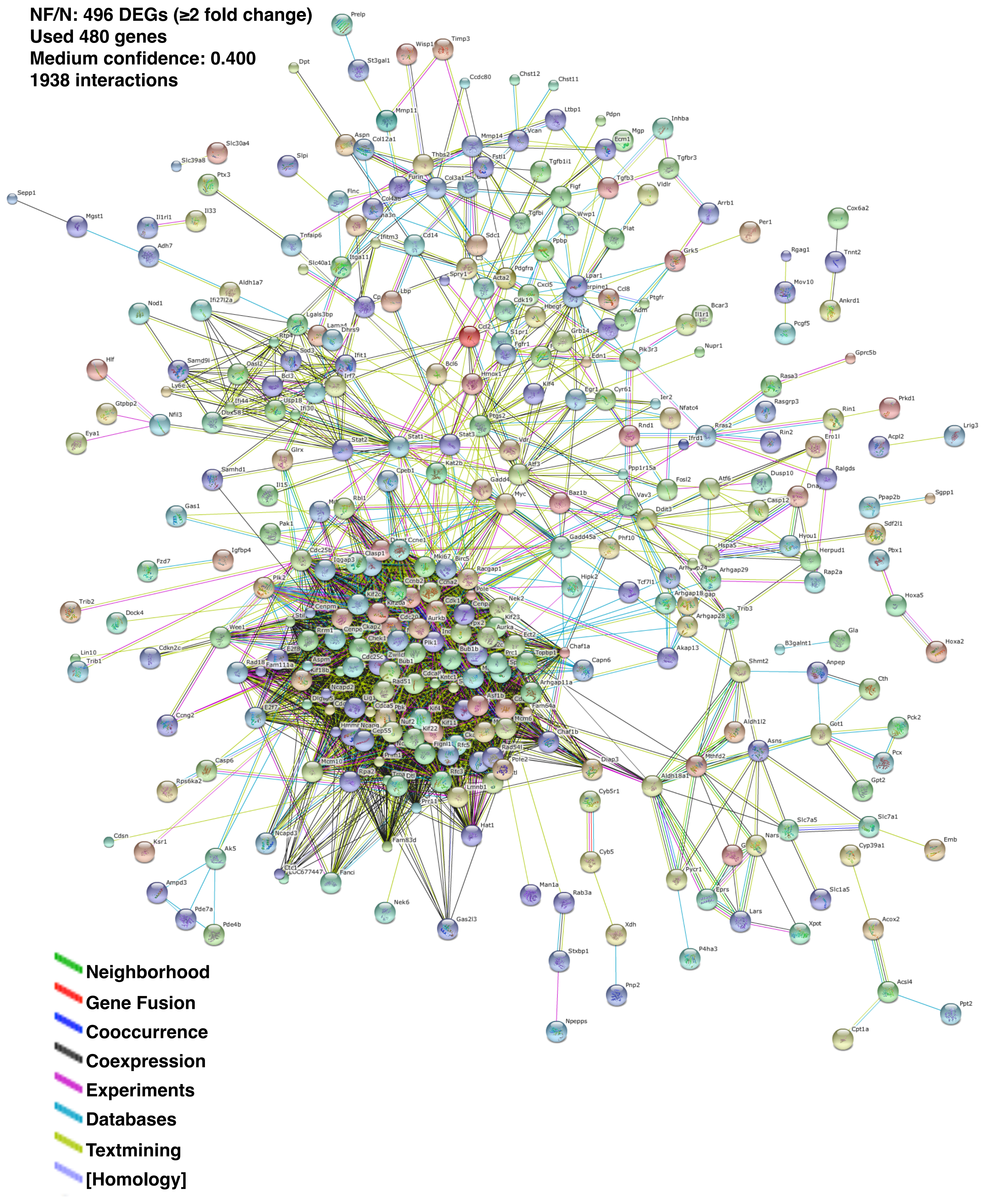

S2 Fig. The genes regulated by FSK in Normal cells show a high degree of connection. In the figure the network of predicted associations for all DEGs-encoded proteins in NF/N comparison is shown. The STRING analysis of the protein-protein interactions was performed to DEGs with fold change ≥2 in the comparison.
