# Supplementary material for: Protein Kinase A Activation Promotes Cancer Cell Resistance to Glucose Starvation and Anoikis
Source: PLoS Genet. 2016 Mar 15;12(3):e1005931. doi: 10.1371/journal.pgen.1005931 (PMC4792400; doi:10.1371/journal.pgen.1005931)
Supplement: S3 Fig — In the figure the network of predicted associations for all DEGs-encoded proteins in TF/T comparison is shown. The STRING analysis of the protein-protein interactions was performed to DEGs with fold change ≥2 in the comparison. (PDF) [file pgen.1005931.s003.pdf]

TF/T: 208 DEGs (≥2 fold change)
Used 200 genes
Medium confidence : 0.400
141 interactions

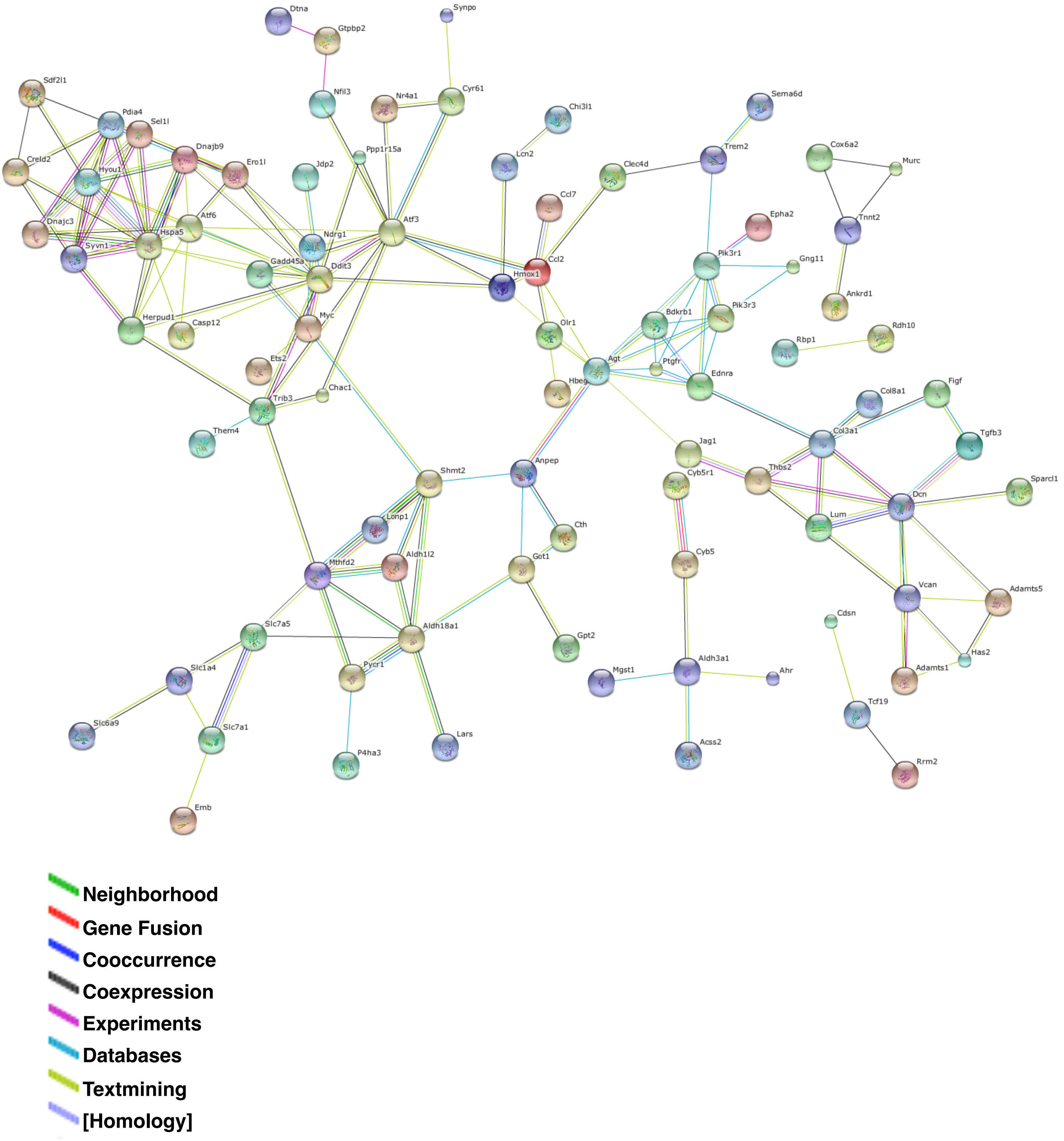

S3 Fig. The genes regulated by FSK in Transformed cells show a low degree of connection. In the figure the network of predicted associations for all DEGs-encoded proteins in TF/T comparison is shown. The STRING analysis of the protein-protein interactions was performed to DEGs with fold change ≥2 in the comparison.
