# Supplementary material for: Protein Kinase A Activation Promotes Cancer Cell Resistance to Glucose Starvation and Anoikis
Source: PLoS Genet. 2016 Mar 15;12(3):e1005931. doi: 10.1371/journal.pgen.1005931 (PMC4792400; doi:10.1371/journal.pgen.1005931)
Supplement: S4 Fig — The STRING analysis of the protein-protein interactions was performed using proteins with spot variation ≥10% in NF/N (A) and TF/T (B) comparisons. (PDF) [file pgen.1005931.s004.pdf]

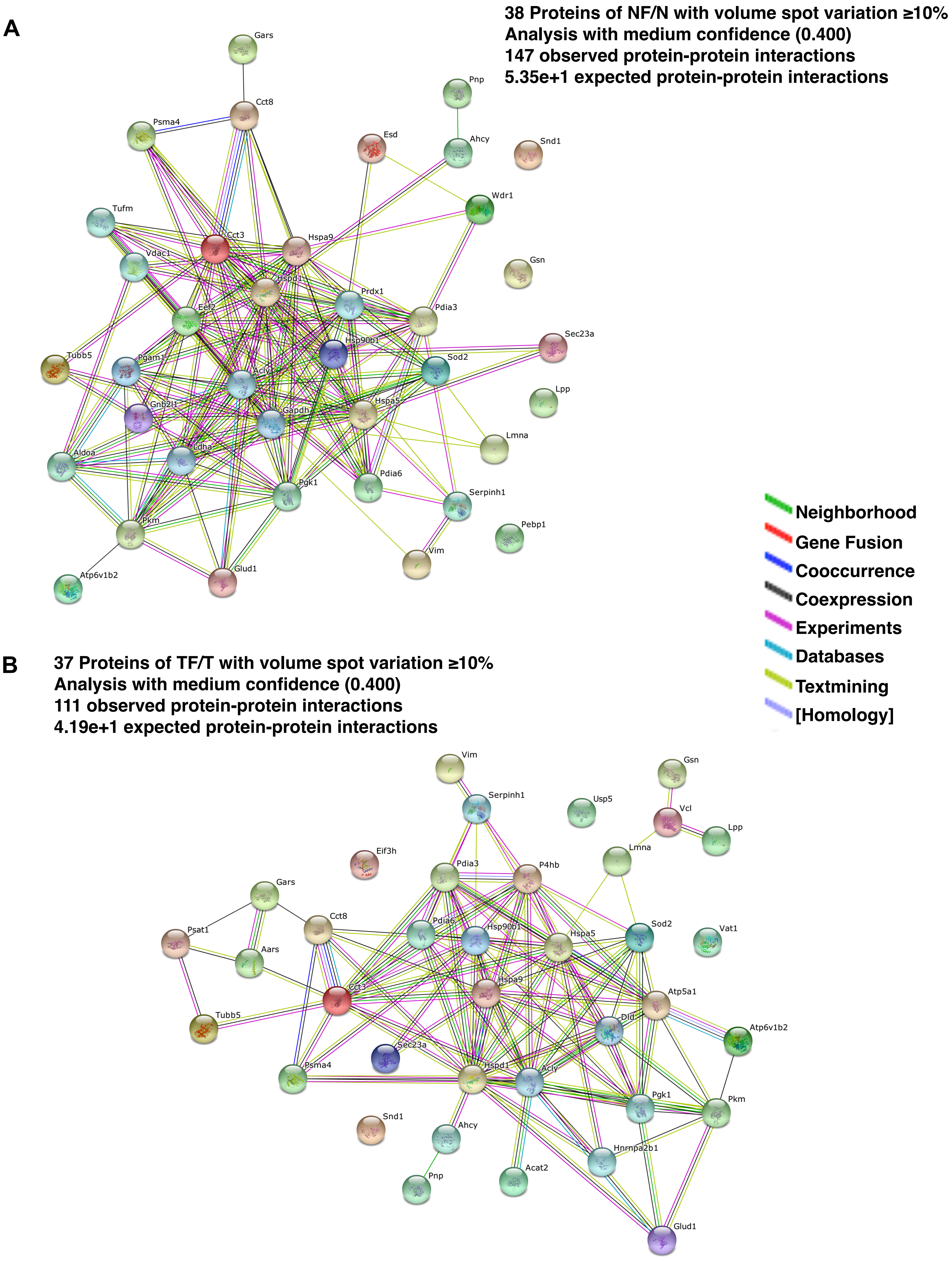

S4 Fig. Network of predicted associations for all the differentially expressed proteins identified by 2-DIGE. The STRING analysis of the protein-protein interactions was performed using proteins with spot variation ≥10% in NF/N (A) and TF/T (B) comparisons.
