# Supplementary material for: Protein Kinase A Activation Promotes Cancer Cell Resistance to Glucose Starvation and Anoikis
Source: PLoS Genet. 2016 Mar 15;12(3):e1005931. doi: 10.1371/journal.pgen.1005931 (PMC4792400; doi:10.1371/journal.pgen.1005931)
Supplement: S5 Fig — The heatmap shows the result obtained by applying the PIANO tool to gene (A) and protein (B) datasets separately. In particular, the top 10-ranked pathways associated to each comparison, NF/N and TF/T, are shown. The different color of the heatmap represents the rank position of the pathway in the two different comparisons. (PDF) [file pgen.1005931.s005.pdf]

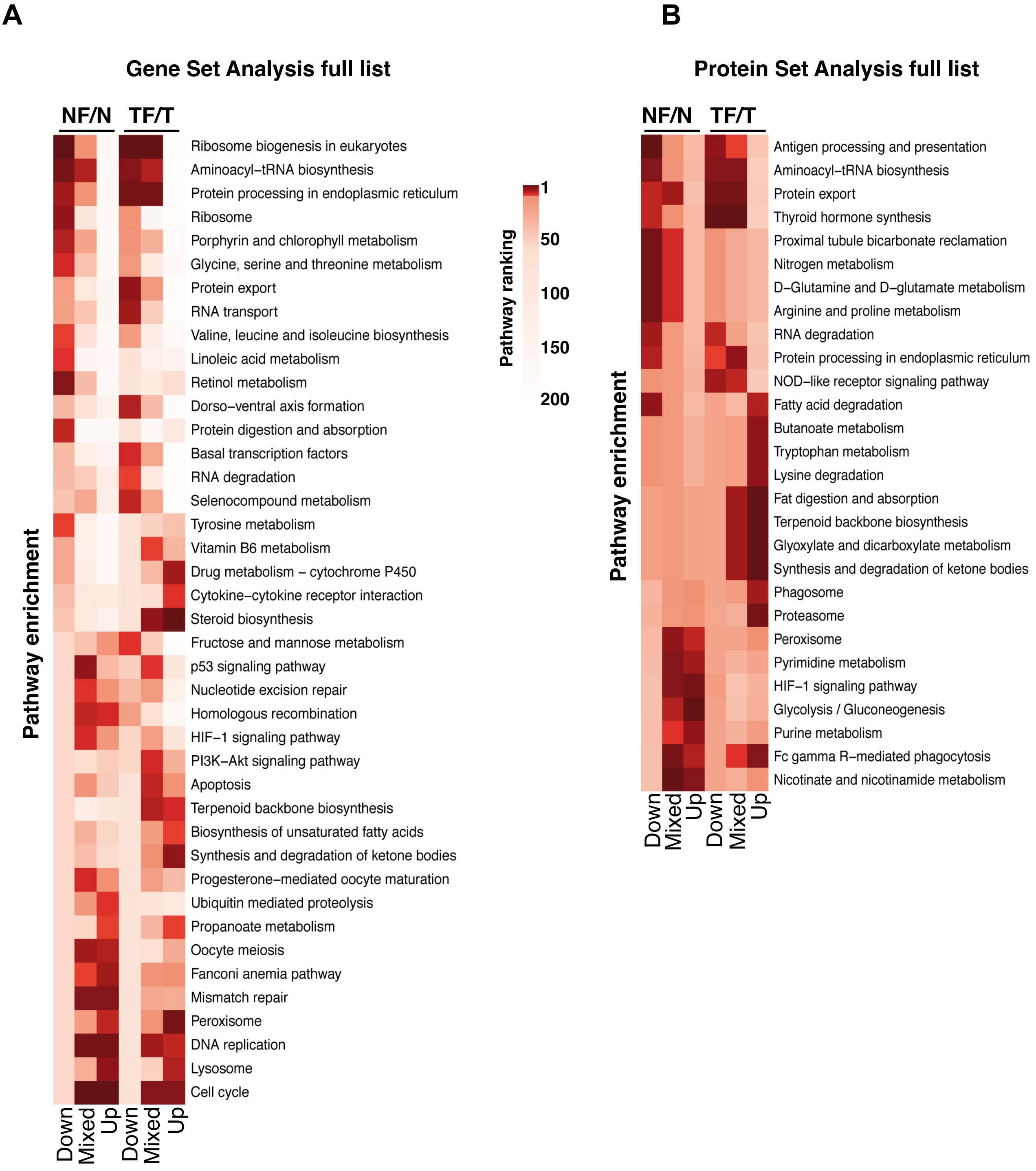

S5 Fig. Analysis of transcriptomic and proteomic data using PIANO method. The heatmap shows the result obtained by applying the PIANO tool to gene (A) and protein (B) datasets separately. In particular, the top 10-ranked pathways associated to each comparison, NF/N and TF/T, are shown. The different color of the heatmap represents the rank position of the pathway in the two different comparisons.
