# Supplementary material for: Protein Kinase A Activation Promotes Cancer Cell Resistance to Glucose Starvation and Anoikis
Source: PLoS Genet. 2016 Mar 15;12(3):e1005931. doi: 10.1371/journal.pgen.1005931 (PMC4792400; doi:10.1371/journal.pgen.1005931)
Supplement: S6 Fig — The analysis shown here was performed in cells cultured for 72h in LG and daily treated with DMSO or 10μM FSK. A-B mRNA expression of UPR-related genes was analyzed by qPCR for Transformed (A) and Normal (B) cells. mRNA expression levels in FSK-treated cells are reported as change (n-fold) with respect to the amount of relative mRNA expressed in untreated cells, using β-actin mRNA as internal control. C Agarose gel electrophoresis was performed to detect the unspliced and spliced forms of Xbp1. All data represent the average of three independent experiments. The error bar indicates the standard deviation while the asterisks indicate statistical significance determined by Student’s t-test (*p<0.05, **p<0.01, ***p<0.001; n.s. not significant). (PDF) [file pgen.1005931.s006.pdf]

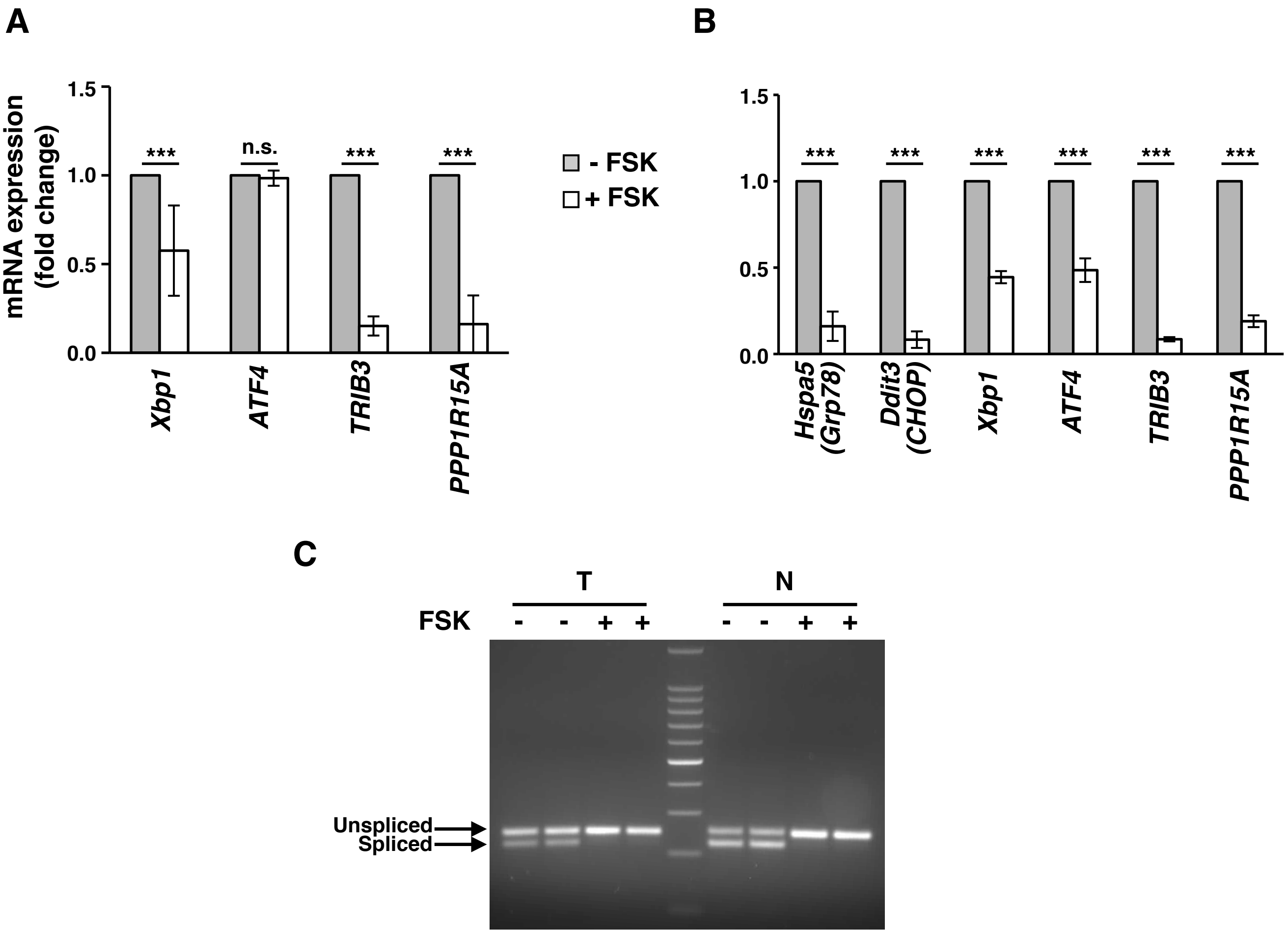

S6 Fig. The FSK treatment attenuates UPR in both Normal and Transformed cells. The analysis shown here was performed in cells cultured for 72h in LG and daily treated with DMSO or 10μM FSK. **A-B** mRNA expression of UPR-related genes was analyzed by qPCR for Transformed (A) and Normal (B) cells. mRNA expression levels in FSK-treated cells are reported as change (n-fold) with respect to the amount of relative mRNA expressed in untreated cells, using β-actin mRNA as internal control. **C** Agarose gel electrophoresis was performed to detect the unspliced and spliced forms of Xbp1. All data represent the average of three independent experiments. The error bar indicates the standard deviation while the asterisks indicate statistical significance determined by Student's t-test (*p<0.05; **p<0.01; ***p<0.001; n.s., not significant).
