# Supplementary material for: Protein Kinase A Activation Promotes Cancer Cell Resistance to Glucose Starvation and Anoikis
Source: PLoS Genet. 2016 Mar 15;12(3):e1005931. doi: 10.1371/journal.pgen.1005931 (PMC4792400; doi:10.1371/journal.pgen.1005931)
Supplement: S7 Fig — A PKA activation was evaluated by Western blot analysis of p-(Ser/Thr) PKA substrates and pCREB S133 in Transformed cells daily treated with FSK and/or 2μM H89. B The cellular morphology of the cells -/+ FSK and -/+H89 was observed at 96h of culture and representative microscopy images are shown. C-E Different analyses were performed to evaluate the autophagy in Transformed cells -/+FSK and -/+H89. C Western blot analysis of Beclin1 expression level in cells -/+FSK. D-E Evaluation of LC3B-I conversion in LC3B-II by Western blot (D) and staining with 50μM MDC (E). Precisely, in these last analyses (72h of culture) the cells were treated with FSK 1h before the addition of 10μM H89 to -/+FSK samples and then were collected after additional 9h. The cells with MDC were analyzed using fluorescence microscopy at 60X magnification. Scale bar 10μm. All data are representative images of three independent experiments. (PDF) [file pgen.1005931.s007.pdf]

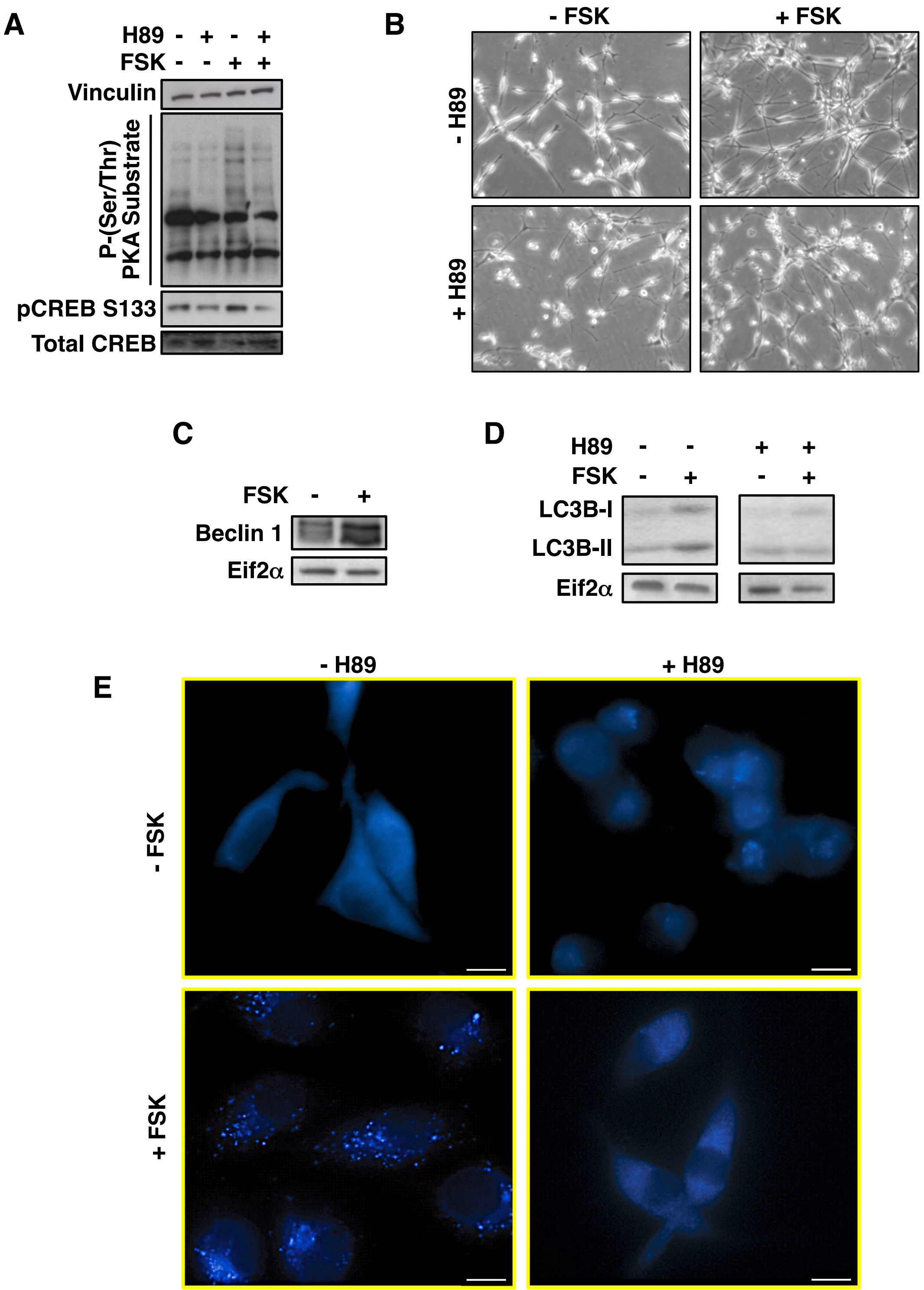


**S7 Fig. The induction of the PKA pathway mediates the autophagy activation in Transformed cells in glucose deprivation. A** PKA activation was evaluated by Western blot analysis of p-(Ser/Thr) PKA substrates and pCREB S133 in Transformed cells daily treated with FSK and/or 2µM H89. **B** The cellular morphology of the cells -/+ FSK and -/+H89 was observed at 96h of culture and representative microscopy images are shown. **C-E** Different analyses were performed to evaluate the autophagy in Transformed cells -/+FSK and -/+H89. **C** Western blot analysis of Beclin1 expression level in cells -/+FSK. **D-E** Evaluation of LC3B-I conversion in LC3B-II by Western blot (D) and staining with 50µM MDC (E). Precisely, in these last analyses (72h of culture) the cells were treated with FSK 1h before the addition of 10µM H89 to -/+FSK samples and then were collected after additional 9h. The cells with MDC were analyzed using fluorescence microscopy at 60X magnification. Scale bar 10µm. All data are representative images of three independent experiments.
