# Supplementary material for: Protein Kinase A Activation Promotes Cancer Cell Resistance to Glucose Starvation and Anoikis
Source: PLoS Genet. 2016 Mar 15;12(3):e1005931. doi: 10.1371/journal.pgen.1005931 (PMC4792400; doi:10.1371/journal.pgen.1005931)
Supplement: S8 Fig — Transcriptional data from microarray analysis regarding glutamine metabolism-related genes in Transformed cells at 72h of culture in LG, daily treated with DMSO or FSK. Data express the ratio in TF/T comparison. (PDF) [file pgen.1005931.s008.pdf]

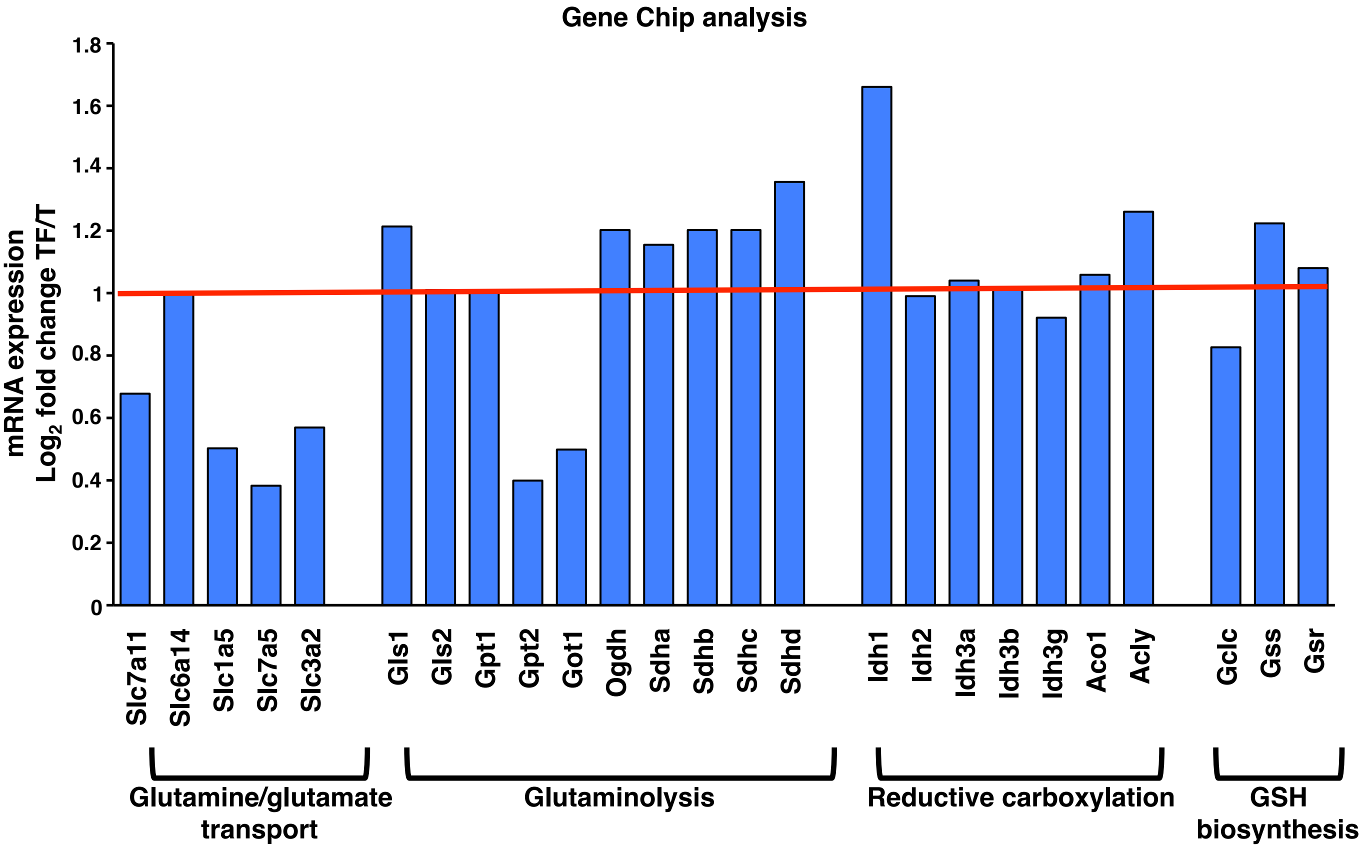

S8 Fig. The treatment with FSK induces a relevant change in the expression of genes related to the glutamine metabolism. Transcriptional data from microarray analysis regarding glutamine metabolism-related genes in Transformed cells at 72h of culture in LG, daily treated with DMSO or FSK. Data express the ratio in TF/T comparison.
