# Supplementary material for: Protein Kinase A Activation Promotes Cancer Cell Resistance to Glucose Starvation and Anoikis
Source: PLoS Genet. 2016 Mar 15;12(3):e1005931. doi: 10.1371/journal.pgen.1005931 (PMC4792400; doi:10.1371/journal.pgen.1005931)
Supplement: S9 Fig — MDA-MB-231 cells were analyzed upon daily treatment with FSK and 2μM H89. A PKA activation by Western blot analysis of p-(Ser/Thr) PKA substrates and pCREB S133. B Microscopy images of the cells were collected at 72h of culture. C Western blot analysis of Grp78 and CHOP was performed at 48h. D-E To analyze the effects of PKA inhibitor H89 on FSK-dependent induced autophagy, Western blot analysis of LC3B-I conversion in LC3B-II (D) and the staining with 50μM MDC were performed. Precisely, in these last analyses (48h of culture) the cells were treated with FSK 1h before the addition of 10μM H89 to -/+FSK samples and then were collected after additional 9h. The cells with MDC were analyzed using fluorescence microscopy at 60X magnification. Scale bar 10μm. F-H Cells were transfected with siRNA (control–siCTRL- or for PKAcat α –siPKAc-) after the medium change (directly in LG medium) and then daily treated with DMSO or FSK for 48h. F The expression of PKAcat α was detected by Western blot. PKA activity was evaluated by ELISA assay in total cellular extracts. G Microscopy pictures were collected and trypan blue exclusion assay was performed. H Western blot analysis of LC3B-I conversion in LC3B-II was performed. All data represent the average of at least three independent experiments. *p<0.05, **p<0.01, ***p<0.001 Student’s t-test. (PDF) [file pgen.1005931.s009.pdf]

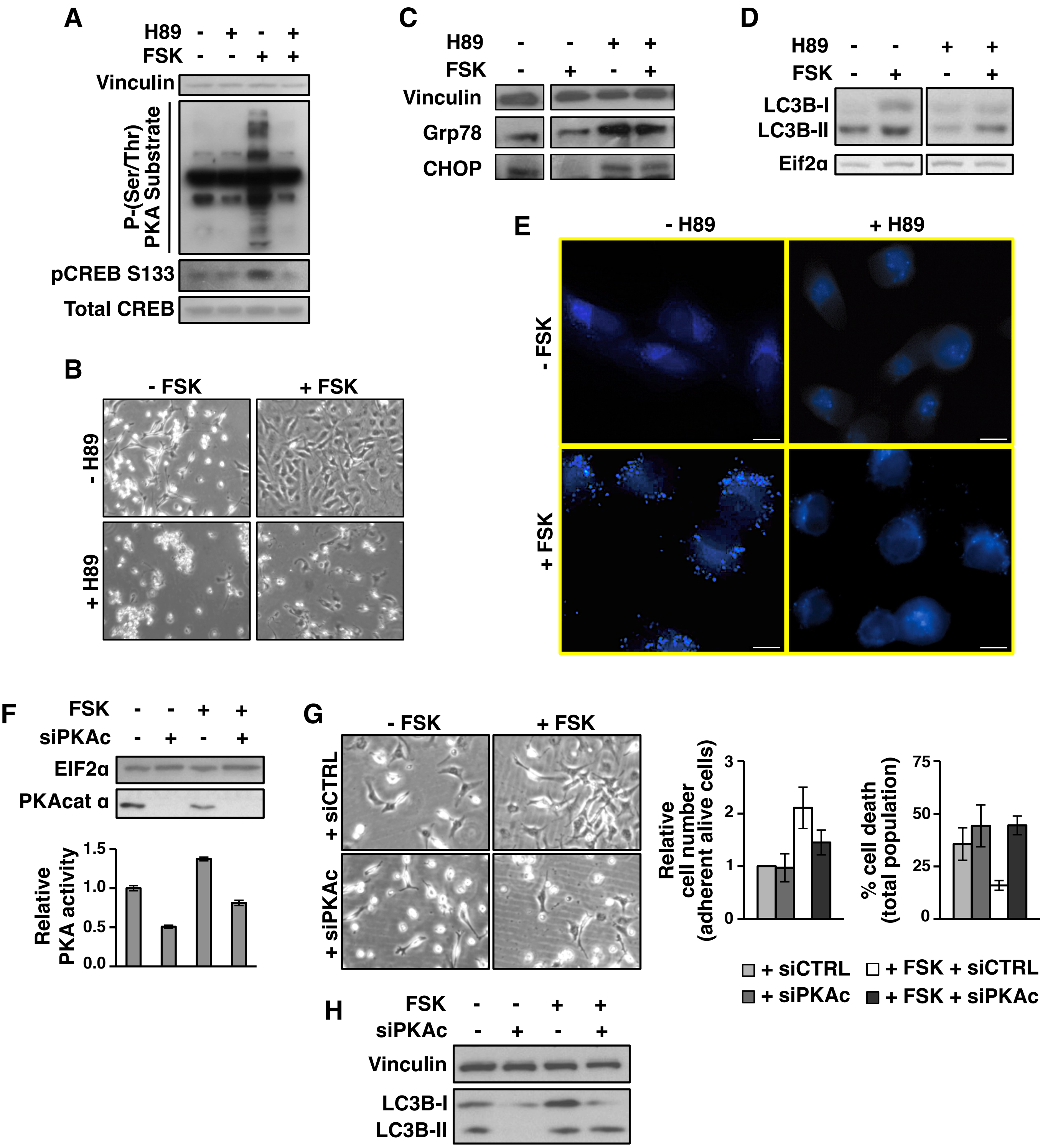

S9 Fig. The inhibition of PKA counteracts the protective effects of FSK in MDA-MB-231. MDA-MB-231 cells were analyzed upon daily treatment with FSK and 2µM H89. **A** PKA activation by Western blot analysis of p-(Ser/Thr) PKA substrates and pCREB S133. **B** Microscopy images of the cells were collected at 72h of culture. **C** Western blot analysis of Grp78 and CHOP was performed at 48h. **D-E** To analyze the effects of PKA inhibitor H89 on FSK-dependent induced autophagy, Western blot analysis of LC3B-I conversion in LC3B-II (D) and the staining with 50µM MDC were performed. Precisely, in these last analyses (48h of culture) the cells were treated with FSK 1h before the addition of 10µM H89 to -/+FSK samples and then were collected after additional 9h. The cells with MDC were analyzed using fluorescence microscopy at 60X magnification. Scale bar 10µm. **F-H** Cells were transfected with siRNA (control –siCTRL- or for PKAcat α –siPKAc-) after the medium change (directly in LG medium) and then daily treated with DMSO or FSK for 48h. **F** The expression of PKAcat α was detected by Western blot. PKA activity was evaluated by ELISA assay in total cellular extracts. **G** Microscopy pictures were collected and trypan blue exclusion assay was performed. **H** Western blot analysis of LC3B-I conversion in LC3B-II was performed. All data represent the average of at least three independent experiments. $^{*}p<0.05$, $^{**}p<0.01$, $^{***}p<0.001$ Student's t-test.
