# Supplementary material for: Protein Kinase A Activation Promotes Cancer Cell Resistance to Glucose Starvation and Anoikis
Source: PLoS Genet. 2016 Mar 15;12(3):e1005931. doi: 10.1371/journal.pgen.1005931 (PMC4792400; doi:10.1371/journal.pgen.1005931)
Supplement: S10 Fig — A Schematic representation of FSK-mediated activation of PKA and Epac proteins upon intracellular cAMP enhancement. To analyze the effect of Epac inhibition, the cells were daily treated with FSK and/or 1μM Epac inhibitor (ESI-09). B Cells -/+FSK and -/+ESI-09 were counted with trypan blue at 72h of culture. C To analyze the effects of Epac inhibitor ESI-09 in the FSK-dependent induced autophagy, MDC staining was performed in viable cells at 72h. D Protein levels of Grp78 and CHOP were analyzed by Western blot at 48h of culture. E Cells were treated with 10μM ESI-09 for 8h and counted with trypan blue. All data represent the average of at least three independent experiments. *p<0.05, **p<0.01, ***p<0.001 Student’s t-test. (PDF) [file pgen.1005931.s010.pdf]

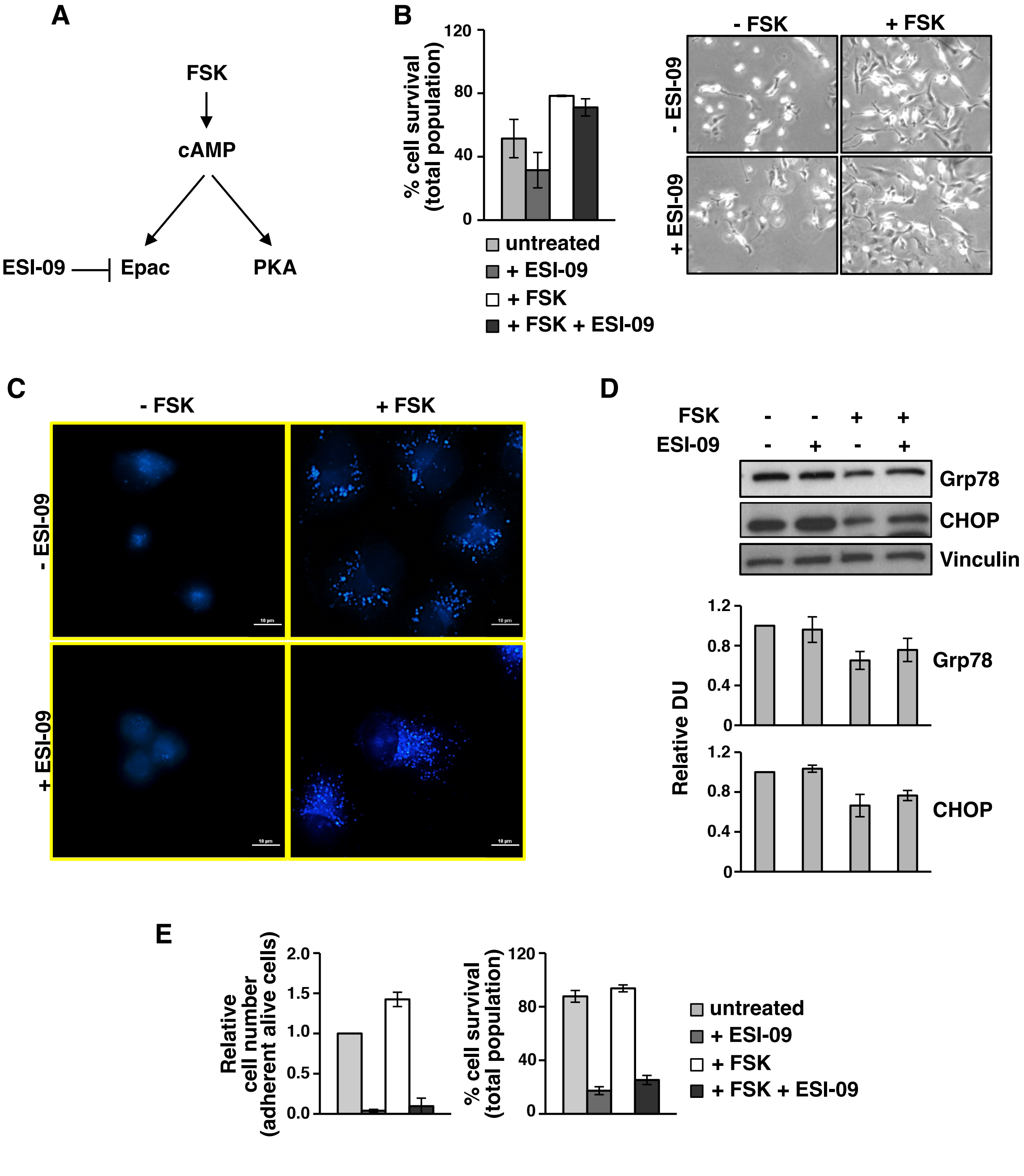

S10 Fig. FSK-protective role is not mediated by Epac. A Schematic representation of FSK-mediated activation of PKA and Epac proteins upon intracellular cAMP enhancement. To analyze the effect of Epac inhibition, the cells were daily treated with FSK and/or 1µM Epac inhibitor (ESI-09). **B** Cells -/+FSK and -/+ESI-09 were counted with trypan blue at 72h of culture. **C** To analyze the effects of Epac inhibitor ESI-09 in the FSK-dependent induced autophagy, MDC staining was performed in viable cells at 72h. **D** Protein levels of Grp78 and CHOP were analyzed by Western blot at 48h of culture. **E** Cells were treated with 10µM ESI-09 for 8h and counted with trypan blue. All data represent the average of at least three independent experiments. *p<0.05, **p<0.01, ***p<0.001 Student's t-test.
