# Supplementary material for: Protein Kinase A Activation Promotes Cancer Cell Resistance to Glucose Starvation and Anoikis
Source: PLoS Genet. 2016 Mar 15;12(3):e1005931. doi: 10.1371/journal.pgen.1005931 (PMC4792400; doi:10.1371/journal.pgen.1005931)
Supplement: S11 Fig — The expression of genes related to the glutamine metabolism was performed in MDA-MB-231 cells daily treated with DMSO or FSK after 48h and 72h of cultivation in LG. mRNA expression of glutamine metabolism-related genes was analyzed by qPCR. mRNA expression levels in FSK-treated cells are reported as fold change with respect to the amount of relative mRNA expressed in untreated cells, using β-actin mRNA as internal control. All data represent the average of at least three independent experiments. Student's t-test (*p<0.05; **p<0.01; ***p<0.001; n.s., not significant). (PDF) [file pgen.1005931.s011.pdf]

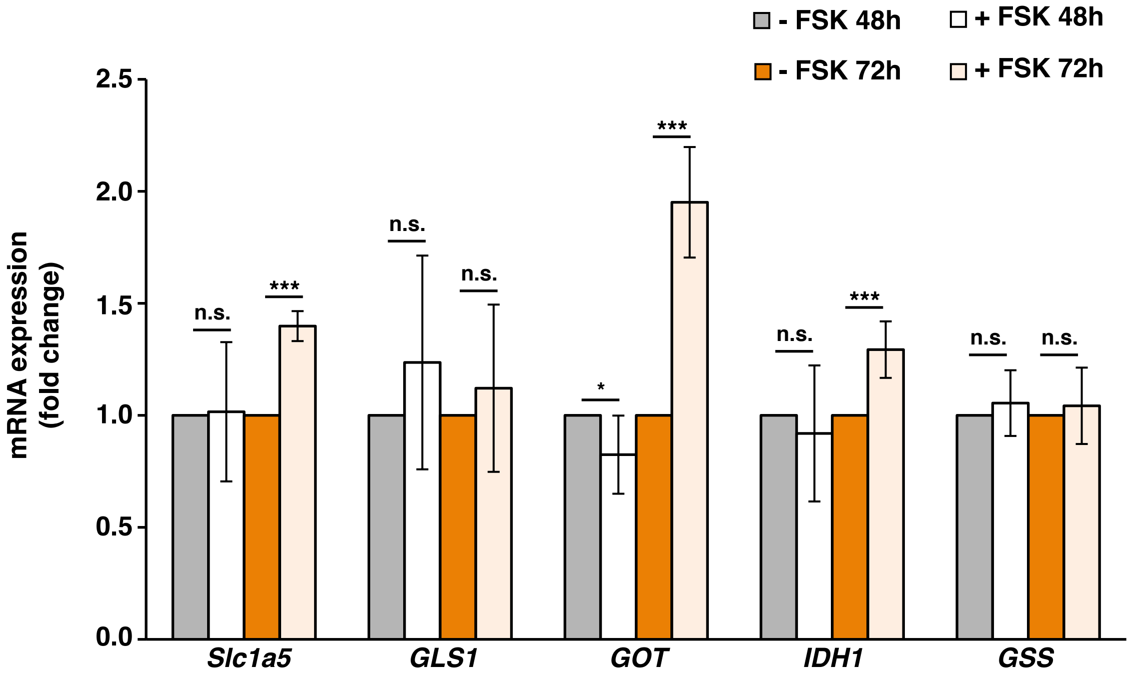

S11 Fig. FSK-treated MDA-MB-231 cells show a different expression of glutamine metabolism-related genes as compared to untreated cells. The expression of genes related to the glutamine metabolism was performed in MDA-MB-231 cells daily treated with DMSO or FSK after 48h and 72h of cultivation in LG. mRNA expression of glutamine metabolism-related genes was analyzed by qPCR. mRNA expression levels in FSK-treated cells are reported as fold change with respect to the amount of relative mRNA expressed in untreated cells, using β-actin mRNA as internal control. All data represent the average of at least three independent experiments. Student's t-test (*$p<0.05$; **$p<0.01$; ***$p<0.001$; n.s., not significant).
