# Supplementary material for: Protein Kinase A Activation Promotes Cancer Cell Resistance to Glucose Starvation and Anoikis
Source: PLoS Genet. 2016 Mar 15;12(3):e1005931. doi: 10.1371/journal.pgen.1005931 (PMC4792400; doi:10.1371/journal.pgen.1005931)
Supplement: S12 Fig — A Graphical representation of the experimental workflow for analyses in anoikis-resistant floating cells. MDA-MB-231 cells were cultured in LG. At 72h, when a significant part of the cell population was detached from the plate, the suspended cells were collected and treated with different drugs (as indicated in the specific figures) or the correspondent vehicle and analyzed 3-6-9-24h later. The same scheme was followed also for Transformed cells but the suspended cells were collected at 96h. See the specific figure for details. B-D MDA-MB-231 floating cells were collected and treated with DMSO, 150μM PKI amide or 10μM H89 for 24h. After treatment, trypan blue exclusion assay (B) and a colony formation assay (C) were performed. In the same samples the PKA activity was evaluated by ELISA assay (D). E MDA-MB-231 floating cells previously transfected with siRNA (control–siCTRL- or for PKAcat α –siPKAc-) were separated from the adherent cells and counted with trypan blue after 8h. F MDA-MB-231 floating cells were collected and treated with DMSO or 1μM Epac inhibitor (ESI-09) for 6h and 24h. After treatment, trypan blue exclusion assay was performed. All data represent the average of at least three independent experiments. *p<0.05, **p<0.01, ***p<0.001 Student’s t-test. (PDF) [file pgen.1005931.s012.pdf]

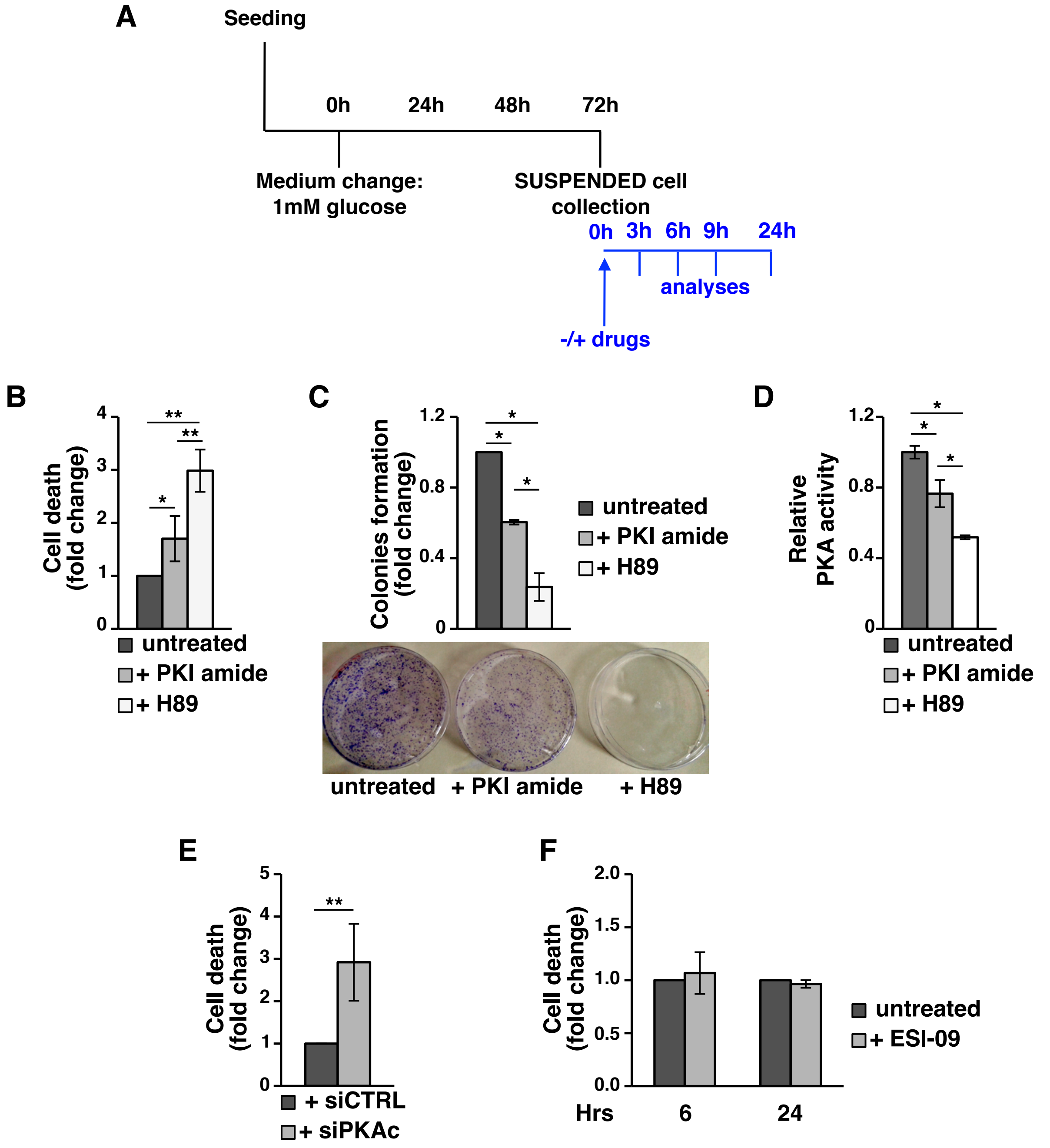

S12 Fig. The inhibition of PKA prevents the resistance to glucose deprivation and *anoikis*. A Graphical representation of the experimental workflow for analyses in *anoikis*-resistant floating cells. MDA-MB-231 cells were cultured in LG. At 72h, when a significant part of the cell population was detached from the plate, the suspended cells were collected and treated with different drugs (as indicated in the specific figures) or the correspondent vehicle and analyzed 3-6-9-24h later. The same scheme was followed also for Transformed cells but the suspended cells were collected at 96h. See the specific figure for details. **B-D** MDA-MB-231 floating cells were collected and treated with DMSO, 150μM PKI amide or 10μM H89 for 24h. After treatment, trypan blue exclusion assay (B) and a colony formation assay (C) were performed. In the same samples the PKA activity was evaluated by ELISA assay (D). **E** MDA-MB-231 floating cells previously transfected with siRNA (control –siCTRL- or for PKAcat α –siPKAc-) were separated from the adherent cells and counted with trypan blue after 8h. **F** MDA-MB-231 floating cells were collected and treated with DMSO or 1μM Epac inhibitor (ESI-09) for 6h and 24h. After treatment, trypan blue exclusion assay was performed. All data represent the average of at least three independent experiments. *p<0.05, **p<0.01, ***p<0.001 Student's t-test.
