# Supplementary material for: Protein Kinase A Activation Promotes Cancer Cell Resistance to Glucose Starvation and Anoikis
Source: PLoS Genet. 2016 Mar 15;12(3):e1005931. doi: 10.1371/journal.pgen.1005931 (PMC4792400; doi:10.1371/journal.pgen.1005931)
Supplement: S13 Fig — A At indicated time points of culture in LG, floating cells were stained with trypan blue and counted to verify whether floating cells were resistant to anoikis. Percentage of floating and dead cells was reported over total population (floating plus adherent cells). All the values have p<0.01 (Student’s t-test) over total cells. B At 96h of culture in LG adherent and suspended cells were separately collected to evaluate PKA pathway activation by Western blot analysis of p-(Ser/Thr) PKA substrates and pCREB S133. C-G All analyses were performed in floating cells collected at 96h of culture in LG. C Floating cells were collected and treated with DMSO or 10μM H89 for 3-6-9h. After treatment, trypan blue exclusion assay was performed. D A colony formation assay was performed with floating cells treated with DMSO or H89 for 6h. The colony formation was evaluated after 14 days of culture in HG as crystal violet absorbance. E PKA activity and LC3B-I/LC3B-II expression were evaluated by Western blot in floating cells treated with H89 for 6h. F-G Viable count by trypan blue was performed in floating cells treated with 10μM BPTES (F) or CQ (G) for 6h. All data represent the average of at least three independent experiments. *p<0.05, **p<0.01, ***p<0.001 Student’s t-test. (PDF) [file pgen.1005931.s013.pdf]

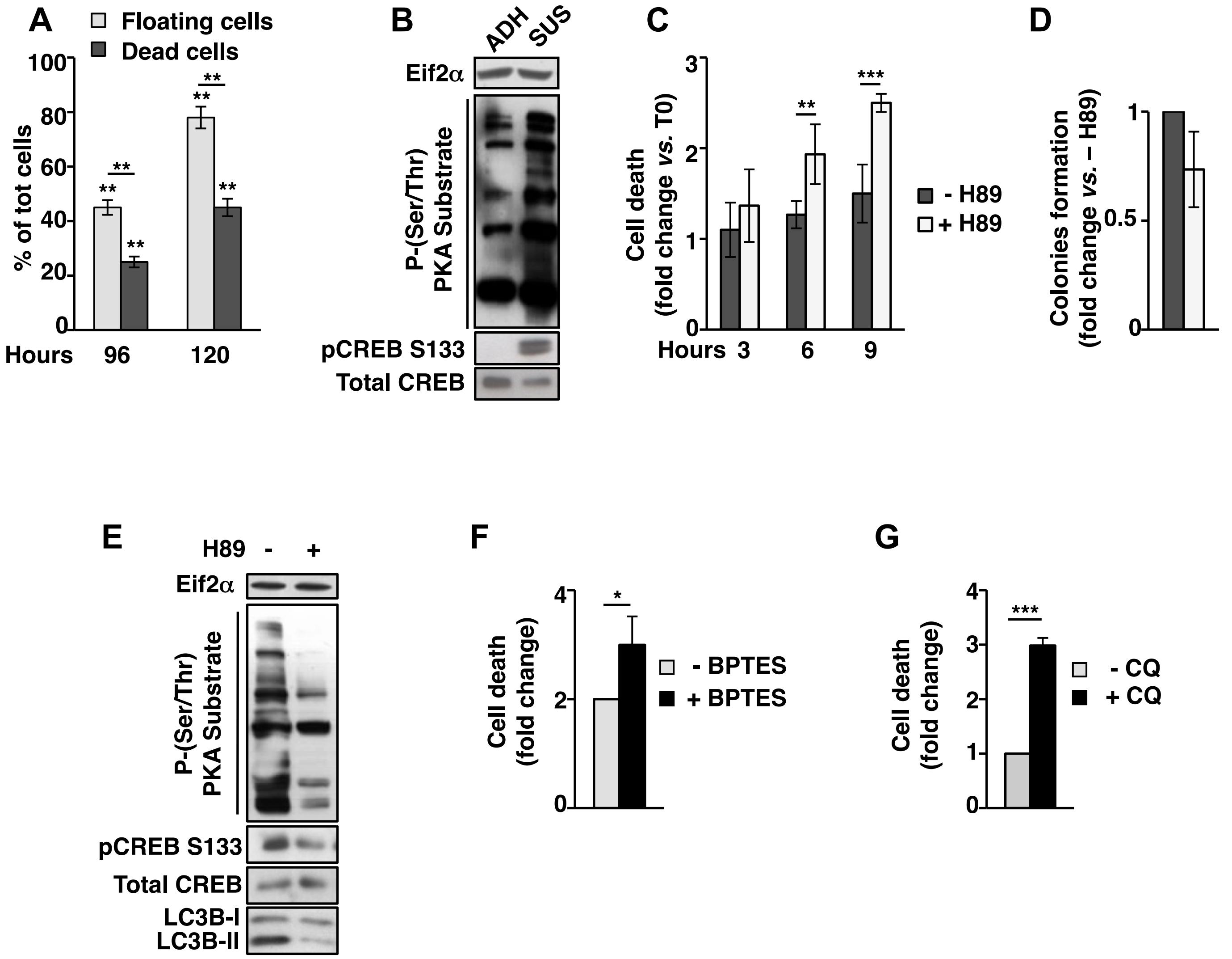

S13 Fig. PKA pathway is endogenously activated in Transformed cells cultured in glucose deprivation, mediating their resistance to *anoikis*. **A** At indicated time points of culture in LG, floating cells were stained with trypan blue and counted to verify whether floating cells were resistant to *anoikis*. Percentage of floating and dead cells was reported over total population (floating plus adherent cells). All the values have $p<0.01$ (Student's t-test) over total cells. **B** At 96h of culture in LG adherent and suspended cells were separately collected to evaluate PKA pathway activation by Western blot analysis of p-(Ser/Thr) PKA substrates and pCREB S133. **C-G** All analyses were performed in floating cells collected at 96h of culture in LG. **C** Floating cells were collected and treated with DMSO or 10μM H89 for 3-6-9h. After treatment, trypan blue exclusion assay was performed. **D** A colony formation assay was performed with floating cells treated with DMSO or H89 for 6h. The colony formation was evaluated after 14 days of culture in HG as crystal violet absorbance. **E** PKA activity and LC3B-I/LC3B-II expression were evaluated by Western blot in floating cells treated with H89 for 6h. **F-G** Viable count by trypan blue was performed in floating cells treated with 10μM BPTES (F) or CQ (G) for 6h. All data represent the average of at least three independent experiments. $^*p<0.05$, $^{**}p<0.01$, $^{***}p<0.001$ Student's t-test.
