# Supplementary material for: Protein Kinase A Activation Promotes Cancer Cell Resistance to Glucose Starvation and Anoikis
Source: PLoS Genet. 2016 Mar 15;12(3):e1005931. doi: 10.1371/journal.pgen.1005931 (PMC4792400; doi:10.1371/journal.pgen.1005931)
Supplement: S14 Fig — A MDA-MB-231 cells treated with FSK were collected and stained with fluorescent substrate of the active caspase-3 and then newly attached to the coverslip using cytospin. Then they were fixed and labeled with the antibody for PKA substrates. The images were visualized by using fluorescent microscopy at 60X magnification. Scale bar 10μm. B PKA activation and LC3B-I/LC3B-II expression were evaluated by Western blot on floating cells treated with 10μM H89 for 6h. All data are representative images of three independent experiments. (PDF) [file pgen.1005931.s014.pdf]

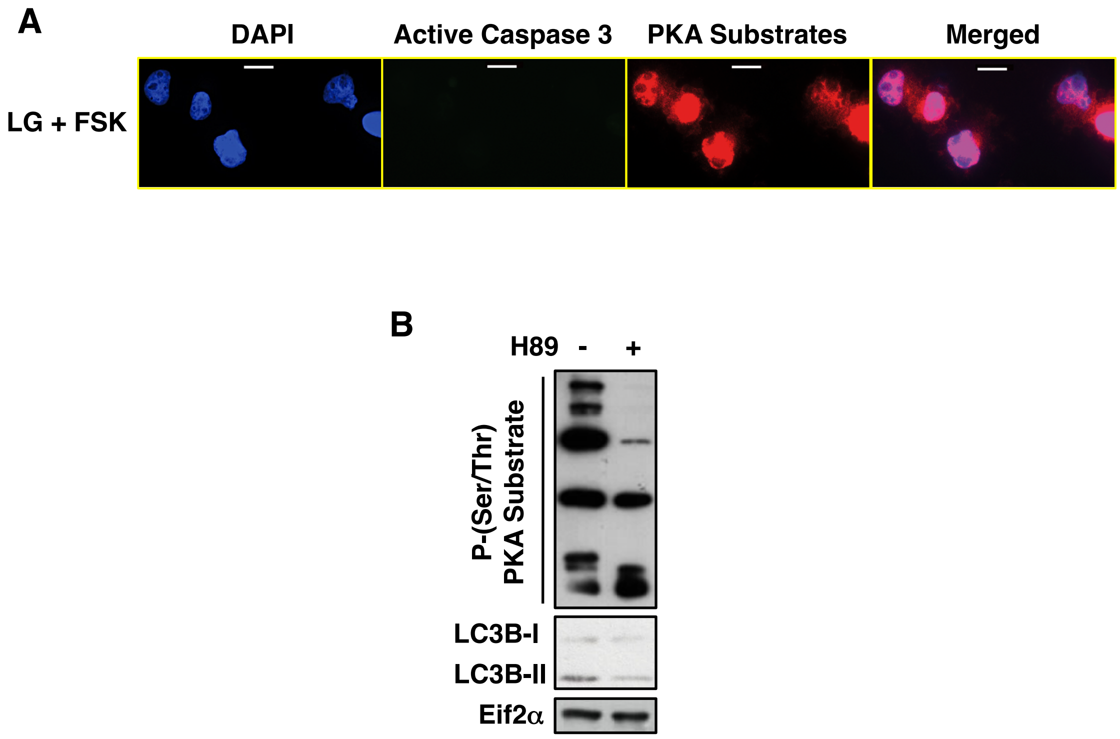

S14 Fig. A MDA-MB-231 cells treated with FSK were collected and stained with fluorescent substrate of the active caspase-3 and then newly attached to the coverslip using cytospin. Then they were fixed and labeled with the antibody for PKA substrates. The images were visualized by using fluorescent microscopy at 60X magnification. Scale bar 10μm. **B** PKA activation and LC3B-I/LC3B-II expression were evaluated by Western blot in floating cells treated with 10μM H89 for 6h. All data are representative images of three independent experiments.
