# Supplementary material for: Protein Kinase A Activation Promotes Cancer Cell Resistance to Glucose Starvation and Anoikis
Source: PLoS Genet. 2016 Mar 15;12(3):e1005931. doi: 10.1371/journal.pgen.1005931 (PMC4792400; doi:10.1371/journal.pgen.1005931)
Supplement: S15 Fig — A-H For the analyses of -/+FSK MIA PaCa-2 cells were cultured in LG and daily treated with DMSO or FSK. A Microscopy images of cell morphology. B PKA activity after FSK treatment was evaluated by Western blot analysis of p-(Ser/Thr) PKA substrates and pCREB S133. C Protein levels of Grp78 and CHOP were analyzed by Western blot at 72h of culture. D MDC staining was performed in viable cells. E Trypan blue exclusion assay was performed in cells -/+ FSK, treated or not with 30μM CQ for the last 24h of culture. Data are plotted as fold change over the equivalent control sample (- CQ). F Expression level of Grp78 and CHOP proteins was analyzed by Western blot in cells -/+ FSK and -/+ CQ with densitometric values. G qPCR was performed in cells -/+ FSK at 72h of culture. H -/+ FSK cells were treated with BPTES for 24h and counted at 96h of culture. Percentage of reduction after the treatment is shown. I-L All analyses were performed in cells grown in LG in wells coated with poly-HEMA. All treatments were performed at 96h of culture. I-J Cells were treated with DMSO or 10μM H89 for 9h. After treatment, PKA activity was evaluated by Western blot (I) and trypan blue exclusion assay was performed (J). K-L Viable count by trypan blue was performed on cells treated with 20μM BPTES (K) or 30μM CQ (L) for 9h. All data represent the average of at least three independent experiments. *p<0.05, **p<0.01, ***p<0.001, Student’s t-test. (PDF) [file pgen.1005931.s015.pdf]

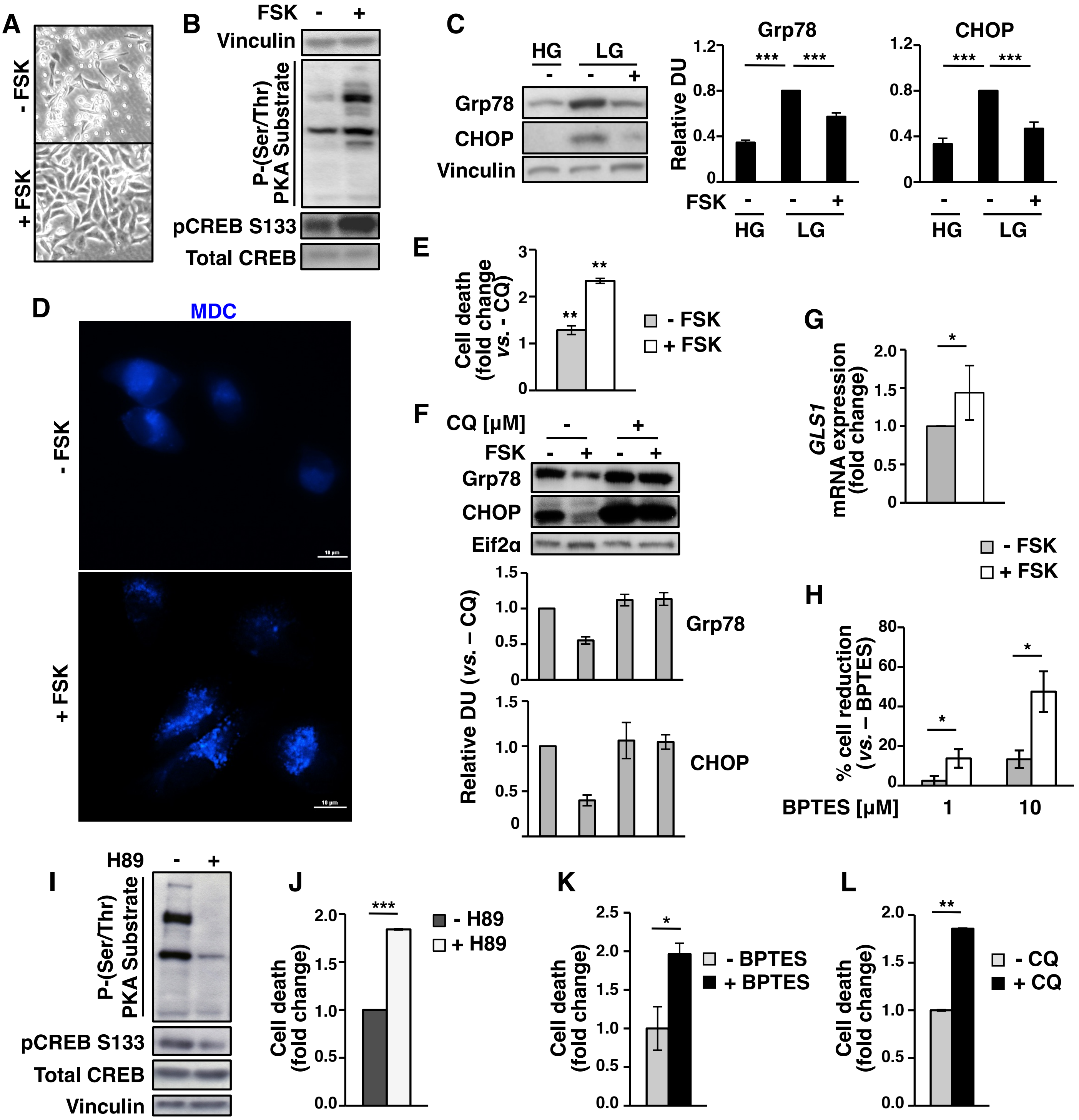

S15 Fig. PKA activation protects MIA PaCa-2 pancreatic cancer cells by glucose deprivation-induced cell death. A-H For the analyses of -/+FSK MIA PaCa-2 cells were cultured in LG and daily treated with DMSO or FSK. **A** Microscopy images of cell morphology. **B** PKA activity after FSK treatment was evaluated by Western blot analysis of p-(Ser/Thr) PKA substrates and pCREB S133. **C** Protein levels of Grp78 and CHOP were analyzed by Western blot at 72h of culture. **D** MDC staining was performed in viable cells. **E** Trypan blue exclusion assay was performed in cells -/+ FSK, treated or not with 30µM CQ for the last 24h of culture. Data are plotted as fold change over the equivalent control sample (- CQ). **F** Expression level of Grp78 and CHOP proteins was analyzed by Western blot in cells -/+ FSK and -/+ CQ with densitometric values. **G** qPCR was performed in cells -/+ FSK at 72h of culture. **H** -/+ FSK cells were treated with BPTES for 24h and counted at 96h of culture. Percentage of reduction after the treatment is shown. **I-L** All analyses were performed in cells grown in LG in wells coated with poly-HEMA. All treatments were performed at 96h of culture. **I-J** Cells were treated with DMSO or 10µM H89 for 9h. After treatment, PKA activity was evaluated by Western blot (I) and trypan blue exclusion assay was performed (J). **K-L** Viable count by trypan blue was performed on cells treated with 20µM BPTES (K) or 30µM CQ (L) for 9h. All data represent the average of at least three independent experiments. *p<0.05, **p<0.01, ***p<0.001 Student's t-test.
