# Supplementary material for: Protein Kinase A Activation Promotes Cancer Cell Resistance to Glucose Starvation and Anoikis
Source: PLoS Genet. 2016 Mar 15;12(3):e1005931. doi: 10.1371/journal.pgen.1005931 (PMC4792400; doi:10.1371/journal.pgen.1005931)
Supplement: S16 Fig — A MIA PaCa-2 cells were cultured in LG (1mM glucose as initial concentration). The amount of glucose in the medium was measured using an enzymatic kit. B-C MIA PaCa-2 cells cultured in HG and LG were counted at different time points with Trypan blue, separating adherent and suspended cells. The percentage of suspended cells in total population (B) and of alive suspended cells (C) is shown. (PDF) [file pgen.1005931.s016.pdf]

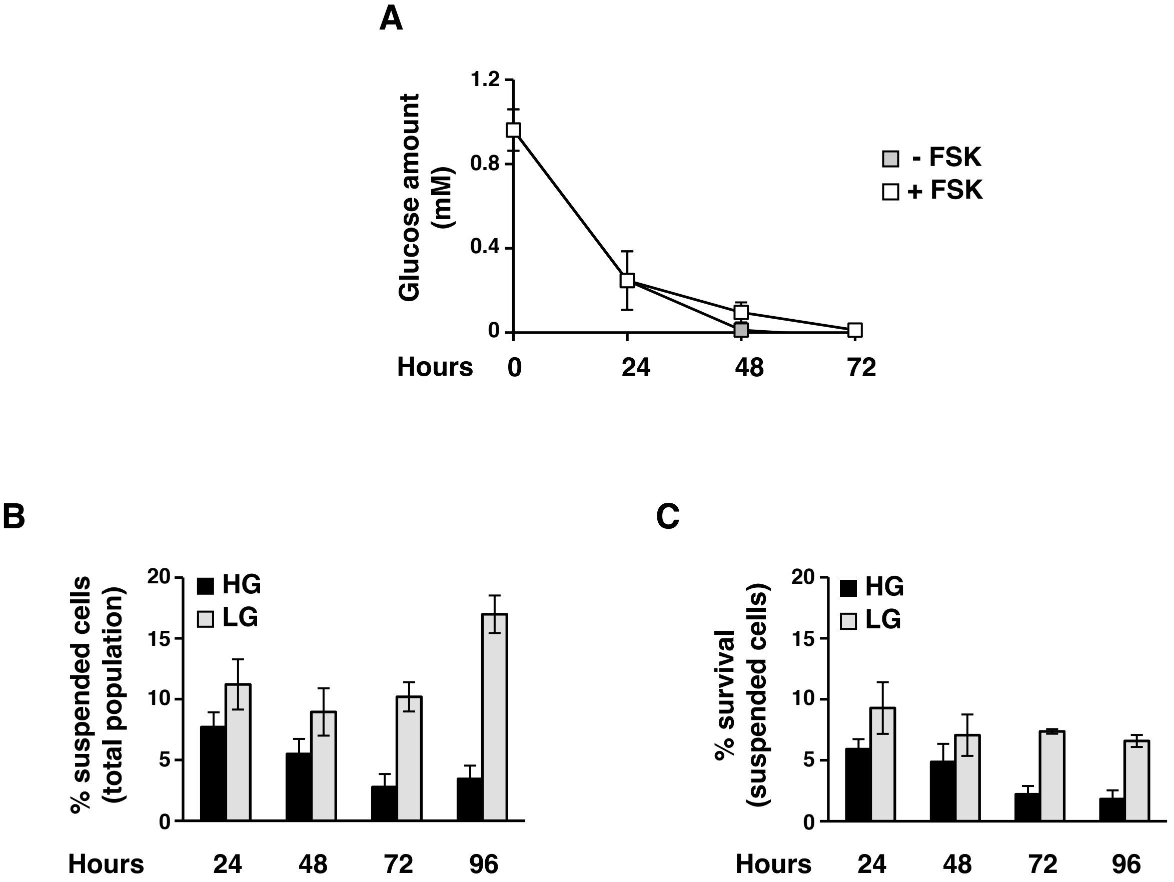

S16 Fig. Glucose depletion induces low *anoikis* resistance in MIA PaCa-2 when cultivated on standard support. A MIA PaCa-2 cells were cultured in LG (1mM glucose as initial concentration). The amount of glucose in the medium was measured using an enzymatic kit. **B-C** MIA PaCa-2 cells cultured in HG and LG were counted at different time points with trypan blue, separating adherent and suspended cells. The percentage of suspended cells in total population (B) and of alive suspended cells (C) is shown.
