# Supplementary material for: Protein Kinase A Activation Promotes Cancer Cell Resistance to Glucose Starvation and Anoikis
Source: PLoS Genet. 2016 Mar 15;12(3):e1005931. doi: 10.1371/journal.pgen.1005931 (PMC4792400; doi:10.1371/journal.pgen.1005931)
Supplement: S1 Text — (PDF) [file pgen.1005931.s024.pdf]

**S1 Text**

**Supplementary methods**

# Protein Kinase A Activation Promotes Cancer Cell Resistance to Glucose Starvation and *Anoikis*

Roberta Palorini[1,2,3]¶, Giuseppina Votta[1,2]¶, Yuri Pirola[4], Humberto De Vitto[1], Sara De Palma[5], Cristina Airoldi[1], Michele Vasso[5], Francesca Ricciardiello[1], Pietro Paolo Lombardi[1], Claudia Cirulli[1], Raffaella Rizzi[4], Francesco Nicotra[1], Karsten Hiller[3], Cecilia Gelfi[5,6], Lilia Alberghina[1,2], Ferdinando Chiaradonna[1,2]*

**Table of contents:**

**- NMR spectroscopy**

**- Metabolite identification**

**- MIAPE-GE Gel Electrophoresis**

**- MIAPE MS Supplementary Information**

**- Stable-isotope labeling experiments: extended protocol**

## NMR spectroscopy

The dried aqueous extracts were rehydrated in 560 ml $D_2O$, buffered in 20 mM sodium phosphate (pD 7.4) containing 0.02 mM 4,4-dimethyl-4-silapentane-1-sulfonic acid (DSS) as an internal standard for both chemical shift and concentration. The NMR spectra of the extracts were recorded at 25 °C on a Bruker Avance III-600 MHz using a 5-mm QCI cryoprobe. Sample temperature was equilibrated inside the spectrometer for 5 min before data acquisition. For each sample *noesygppr1d* and Carr-Purcell-Meliboom-Gill T2 filter *cpmgpr1d* Bruker pulse sequences were acquired with 128 free induction decays (FIDs) and 64 k data points over a spectral width of 20 ppm. A 5-s relaxation delay was incorporated between FIDs. The FID values were multiplied by an exponential function with a 0.3 Hz line broadening factor. Spectra were automatically phased, corrected for baseline and referenced using the library Topspin AU program apk0.noe. Metabolite extraction was performed as described in supplementary information.

## Metabolite identification

Metabolite identification and assignment were performed with the support of 2D NMR experiments, the Biological Magnetic Resonance Data Bank [http://www.bmrb.wisc.edu/] and the Human Metabolome Database [http://www.hmdb.ca/]. Total correlation spectroscopy (TOCSY) and heteronuclear single quantum correlation (HSQC) were performed for representative samples. For all these experiments, 256–512 t1 increments were used and 40-96 transients were collected. The relaxation delays were set at 1.5 s, and the experiments were acquired in the phase sensitive mode. The TOCSY spectra were recorded with a mixing time (spin-lock) of 60 ms.

$^1$H NMR spectra were normalized to the sum of total spectrum integral to minimize the effect of the differences in sample concentration. The regions corresponding to water and DSS were excluded from normalization.

For metabolites quantification, each region of the NMR spectrum defined in Tables S8 (Electronic supplementary material) was then integrated into MestReNova (v 9.0, 2013 Mestrelab Research S.L.) by exploiting the algorithm called GSD (global spectrum deconvolution) (Cobas, C.: Seoane, F.; Domínguez, S.; Sykora, S. A new approach to improving automated analysis of proton NMR spectra through Global Spectral Deconvolution (GSD). *Spectroscopy Europe,* **2010***, 23* vol 23 (1), 2010). Overlapping regions were deconvoluted and absolute quantification performed also for metabolites with resonances in crowded spectral areas (Garcia-Manteiga *et al*, 2011). For each compound, the mean value of the different assigned signals was determined. Six to nine independent samples from at least two different cell cultures were obtained per sample condition.

For the estimation of metabolites whose missing in one or more conditions we applied the same strategy adopted by the portal MetaboAnalyst [www.metaboanalyst.ca/MetaboAnalyst/faces/Home.jsp]: all the missing values were replaced with a small values (the half of the minimum positive values in the original data) assuming to be the detection limit. The assumption of this approach is that most missing values are caused by low abundance metabolites (i.e. below the detection limit) (Xia J, Mandal R, Sinelnikov IV, Broadhurst D, Wishart DS (2012) MetaboAnalyst 2.0--a comprehensive server for metabolomic data analysis. Nucleic Acids Res 40: W127-133).

## MIAPE-GE Gel Electrophoresis

### *General Features*

- Experiments conducted at the CNR –IBFM (Instituto di Bioimmagini e Fisiologia Molecolare) Segrate, MI, Italy
- Electrophoresis type: 2-D DIGE.
- Images: available upon request.
- Image analysis software: DeCyder version 6.5 (GE Healthcare).

- Statistical analysis software: EDA module version 1.0 (GE Healthcare).

*Gel analysis design*

- Normal cells -/+ FSK and Transformed cells -/+ FSK.
- Experimental groups: 4
- Internal standard: Cy3-labelled pool of equal protein amounts from all samples run on each analytic gel.

*Image pre-processing*

- Type: Images were scanned on the Typhoon 9200 Imager (GE Healthcare).
- Software: ImageQuant TL (GE Healthcare).
- Settings:
    - Analytic (quantitative) gels: laser voltages were optimized for each fluorophore prior to scanning to avoid signal saturation. Identical laser settings were then used to scan each gel.
    - Preparative (picking/protein identification) gel: fixed with ethanol/acetic acid and post-stained with Deep Purple, scanned independently from analytic gels.
    - All gels were scanned at 100μm resolution.

*Data extraction process*

- Input images: available upon request.
- Feature detection: DeCyder version 6.5, DIA module (GE Healthcare)
    - Automatic detection with estimation of 5000 protein spots
    - Exclusion filter applied to spots with the following features
        - Slope > 1.2
        - Area < 200

        - Volume < 10000
    - DIA workspaces were manually edited to eliminate gel artefacts and to reinclude any incorrectly excluded spots.
- Matching algorithm: DeCyder version 6.5, BVA module (GE Healthcare)
    - Automatic matching with 50 manually identified landmark protein spots per gel
    - Spot matches were manually edited
    - Match on 90% of gels required.
- Feature quantitation: Decyder version 6.5
    - Background subtraction: DeCyder version 6.5
    - Normalization: DeCyder version 6.5.

***Data analysis***

- PCA: DeCyder EDA module version 1.0.
- Statistical analysis: DeCyder EDA module version 1.0.
- Type: paired Student's T-test ($p<0.05$); input data: log standardized abundance.
- Power analysis: Lenth power tool; input data: biological variance calculated from standardized spots volumes.

**MIAPE MS Supplementary Information**

***General features***

- Instrument used: MALDI ULTRAFLEX III (Bruker Daltonics).
- Software used: FlexAnalysis v. 3.0, BioTools v. 3.2, Proteinscape 3.0(Bruker Daltonics); MASCOT v 2.2 database

***Input data and parameters***

- Type of MS data: MS and MS/MS spectra.
- Databases queried: MASCOT v2.2, NCBInr 20100918 database(11833178 sequences; 4040378175 residues).
- Taxonomical restrictions: Rodentia (Rodents) (133347 sequences)
- Cleavage agent: Trypsin.
- Allowed number of missed cleavages: 1.
- Permissible amino acid modifications: oxidation (variable) and carbamidomethylation (fixed).
- Mass exclusion tolerance: 30 ppm (PMF), 0,5 Da MS/MS tolerance (LIFT-CID Ultraflex III).
-

***The output from the procedure***

- Data for identified proteins: See Supporting Information Supplementary Table 2

**Stable-isotope labeling experiment: extended protocol**

***Metabolite extraction***

For the intracellular metabolite extraction cells were quickly rinsed with 0.9% NaCl and quenched with a volume of ice-cold methanol. An equal volume of water was added and cells were collected with a cell scraper and transferred in tubes with a volume of chloroform. The extracts were shaken at 1400rpm for 20 minutes at 4ºC. Samples were then centrifuged at 13000rpm for 5 minutes and the aqueous phase was collected and transferred into a GC/MS vial for speed vacuum evaporation at -4ºC using a refrigerated CentriVap concentrator.

***GC/MS analysis***

Metabolite derivatization was performed using an Agilent autosampler. Dried polar metabolites were dissolved in 15uL of 2% methoxyamine hydrochloride in pyridine at 45 °C for 60min. An equal volume of *N*-*tert*-Butyldimethylsilyl-*N*-methyltrifluoroacetamide (MTBSTFA) with 1% *tert*-Butyldimethylchlorosilane (TBDMSCl) was added and held for 60 min at 45 °C. All derivatization reagents were purchased from Sigma-Aldrich. GC/MS analysis was performed using an Agilent 6890GC equipped with a 30-m DB-35MS capillary column. The GC was connected to an Agilent 5975C MS operating under electron impact ionization at 70 eV. The MS source was held at 230 °C and the quadrupole at 150 °C. 1 µL of derivatized sample was injected in splitless mode. Helium was used as carrier gas at a flow rate of 1 mL $min^{-1}$. The GC oven temperature was held at 80 °C for 6 min and increased to 300 °C at 6 °C $min^{-1}$. After 10 min, the temperature was increased to 325 °C at 10 °C $min^{-1}$ for 4 min. Sample data were acquired both in scan (1-600 m/z) and selected ion monitoring (SIM) modes. All metabolites used in this study were previously validated using authentic standards (all standards from Sigma-Aldrich) to confirm mass spectra and retention times.

***Determination of mass isotopomer distributions (MIDs)***

MIDs were determined using the MetaboliteDetector software package (Hiller K. *et al.* 2009 MetaboliteDetector: comprehensive analysis tool for targeted and nontargeted GC/MS based metabolome analysis. Anal Chem. 81, 3429–3439; Wegner A., Sapcariu S. C., Weindl D. & Hiller K. 2013 Isotope cluster-based compound matching in gas chromatography/mass spectrometry for non-targeted metabolomics. Anal Chem 85, 4030–4037). Chemical formulas of respective fragment ions were taken from Wegner A. *et al.* 2014 Fragment formula calculator (FFC): determination of chemical formulas for fragment ions in mass spectrometric data. Anal Chem. 86, 2221–2228.
