# Supplementary material for: Protein Kinase A Activation Promotes Cancer Cell Resistance to Glucose Starvation and Anoikis
Source: PLoS Genet. 2016 Mar 15;12(3):e1005931. doi: 10.1371/journal.pgen.1005931 (PMC4792400; doi:10.1371/journal.pgen.1005931)
Supplement: S2 Text — Table A: Normal and Transformed cells. Table B: MDA- MB-231 cells (PDF) [file pgen.1005931.s025.pdf]

S2 Text

List of primers used for qPCR amplification of target genes

Protein Kinase A Activation Promotes Cancer Cell Resistance to Glucose Starvation and *Anoikis*

Roberta Palorini[1,2,3]¶, Giuseppina Votta[1,2]¶, Yuri Pirola[4], Humberto De Vitto[1], Sara De Palma[5], Cristina Airoldi[1], Michele Vasso[5], Francesca Ricciardiello[1], Pietro Paolo Lombardi[1], Claudia Cirulli[1], Raffaella Rizzi[4], Francesco Nicotra[1], Karsten Hiller[3], Cecilia Gelfi[5,6], Lilia Alberghina[1,2], Ferdinando Chiaradonna[1,2]*

Table A: List of primers used for qPCR of target genes in Normal and Transformed cells

Primer	Forward (5'→3')	Reverse (5'→3')
18S	ttgacggaagggcaccacca	gcaccaccacccacggaatcg
HSPA5	agtggtggccactaatggag	caatccttgcttgatgctga
DDIT3	catacaccaccacacctgaaag	ccgtttcctagttcttccttgc
Xbp1	ctgacgaggttccagaggtg	agcagactctggggaaggac
ATF4	tcgatgctctgtttcgaatg	ggcaacctggtcgactttta
TRIB3	gatgccaagtgtccagtcct	cttgctctcgttccaaaagg
PPP1R15A	aggaccccgagattcctcta	cctggaatcaggggtaaggt
Slc1a5	gactctgttgtagacccctg	cagagaagctggaccatggt
GLS	gctgtgctctattgaagtgaca	ttgggcagaaaccaccatta
GOT1	ccatctttgtcctccatgcct	agcccgaagttcttggagaagg
IDH1	gctggcctttgtatctcagc	ctgactgcacatccccatcgt
GSS	ggttctctggaccaaaaccga	caccttcttagtcccagcca

Table B: List of primers used for qPCR of target genes in MDA-MB-231 and MIA PaCa-2 cells

Primer	Forward (5'→3')	Reverse (5'→3')
ACTIN	tcatgaagtgtgacgttgaca	cctagaagcatttgcggtgcac
HSPA5	cggtctactatgaagcccgt	catctgggtttatgccacgg
DDIT3	ccactcttgaccctgcttct	tggttctcccttggtcttcc
Slc1a5	tgcgtggaggagaataatgg	ggatgatggccagagtgagga
GLS	tggtggcctcaggtgaaaat	ccaagctaggtaacagaccctgttt
GOT1	ggagcagtggaagcagattgc	agcccgaagttcttggagaagg
IDH1	tggctctgtctaagggttgg	accatgtcgtcgatgagcct
GSS	ggaacatccatgtgatccgacga	ccttcttagtcccagccagct
